# Magnetic Hysteresis in a Dysprosium Bis(amide) Complex

**DOI:** 10.1021/jacs.4c08137

**Published:** 2025-02-27

**Authors:** Florian Benner, Rashmi Jena, Aaron L. Odom, Selvan Demir

**Affiliations:** Department of Chemistry, Michigan State University, 578 South Shaw Lane, East Lansing, Michigan 48824, United States

## Abstract

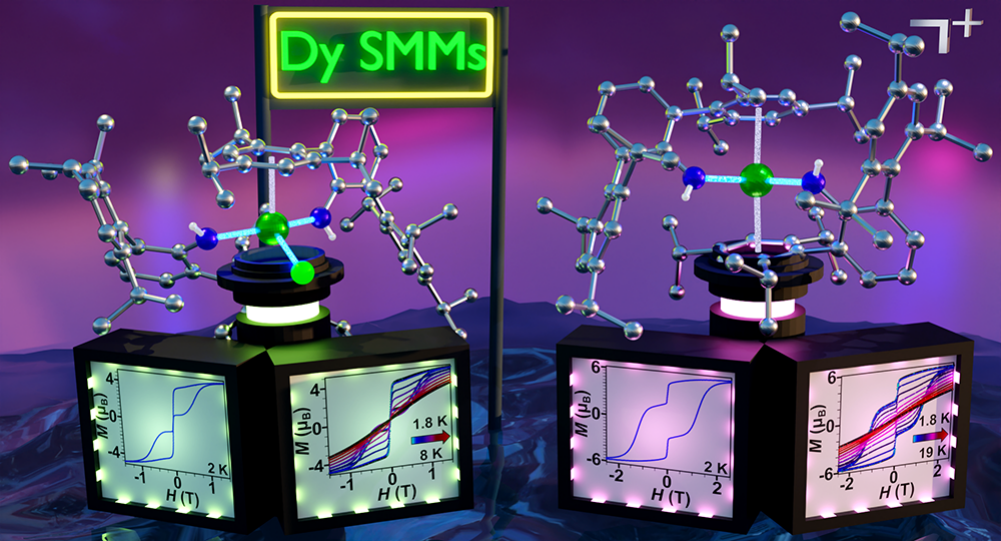

Here, we present the synthesis and characterization of two mononuclear dysprosium molecules. The first complex is neutral and contains two triarylamide ligands coordinating to a Dy^III^ ion that is additionally ligated to a chloride anion, in the form of (NHAr*)_2_DyCl (**1**). Treatment of **1** with Tl[BArF_24_] prompted the removal of the chloride as TlCl from the first coordination sphere to afford the mononuclear Dy^III^ complex, [(NHAr*)_2_Dy][BArF_24_] (**2**), with a cationic [(NHAr*)_2_Dy]^+^ core. **1** and **2** were investigated through single-crystal X-ray diffraction analysis, UV–vis spectroscopy, and SQUID magnetometry. Both compounds are single-molecule magnets with magnetic hysteresis. The determined effective spin-reversal barriers and preattempt times for **1** and **2** are *U*_eff_ = 601(2) cm^–1^ and 598(2) cm^–1^, and τ_0_ = 4.2(1) × 10^–10^ s and 3.1(2) × 10^–10^ s, respectively. *Ab initio* calculations were conducted on both molecules which uncovered the energy of the crystal field states of Dy^III^ and affirmed the effective energy barrier height. Notably, the extrusion of the halide ion has huge ramifications on the magnetic relaxation: While **1** features butterfly hysteresis loops up to 8 K that are closed at zero field at all temperatures probed, **2** exhibits a much higher magnetic blocking temperature of *T*_B_ = 19.0 K and substantial coercivity of *H*_C_ = 1.03 T. Remarkably, both the *T*_B_ and *H*_C_ observed for **2** constitute a record for mononuclear single-molecule magnets where the metal is either sandwiched by two arene ligands or stabilized by amide functionalities, respectively.

## Introduction

Slow magnetic relaxation of molecular origin stems from the presence of a bistable magnetic ground state with an effective energy barrier to spin inversion (*U*_eff_) in paramagnetic complexes.^1^ In the absence of fast quantum tunneling, such single-molecule magnets (SMMs) exhibit magnetic memory effect, which is experimentally captured as open magnetic hysteresis loops, a property highly coveted for potential high-end applications.^2−5^ After the discovery of this physics phenomenon in Mn_12_O_12_,^6−8^ various synthetic approaches led to new types of transition metal-based SMMs.^9,10^ A tremendous breakthrough has been achieved by introducing paramagnetic lanthanide ions as particularly well-suited candidates for SMM design owing to their highly magnetically anisotropic nature originating from large unquenched orbital angular momentum and strong spin–orbit coupling.^11,12^ Hence, lanthanides have been successfully employed to construct mono- and multinuclear SMMs.^13−23^

**Figure 1:**
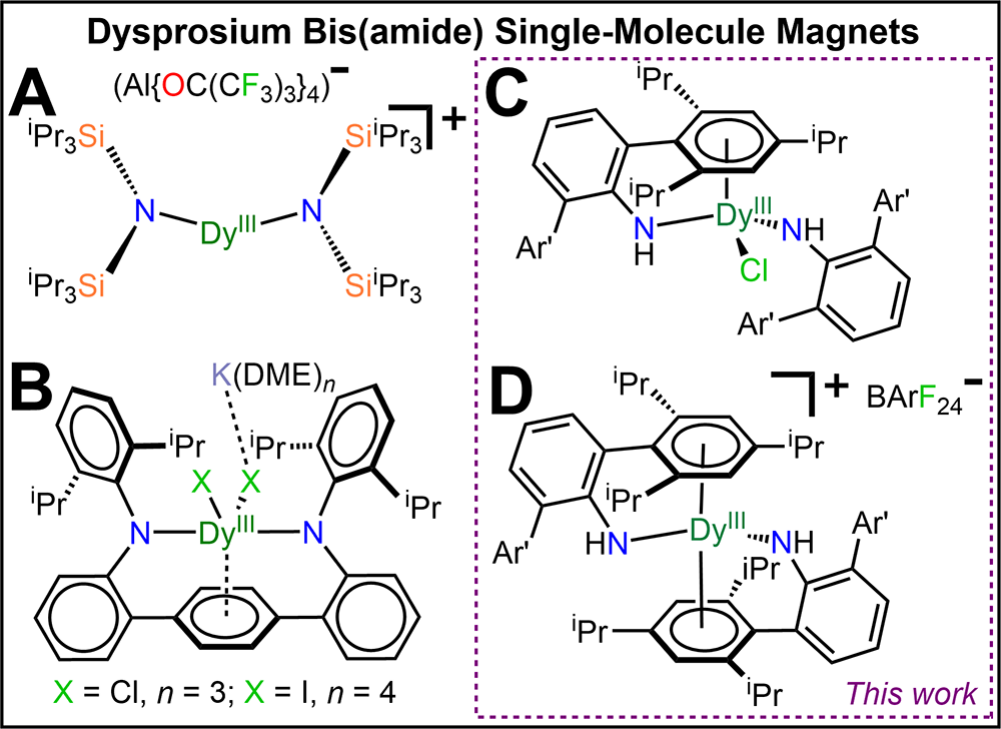
Overview of dysprosium bis(amide) SMMs.

Among the lanthanides, dysprosium in its trivalent state, in fact a Kramers ion, has led to the arguably best SMMs in terms of all relevant, experimentally accessible, metrical parameters, *U*_eff_, *T*_B_ (blocking temperature), and *H*_C_ (coercivity). A Dy^III^ ion is oblate-shaped which corresponds to an electron density that is equatorially expanded. Hence, sandwiching the oblate Dy^III^ ion in between two strongly axial donor ligands increases its single-ion magnetic anisotropy tremendously, typically accompanied by a well-separated ground *M*_J_ = 15/2 state from excited states.^24,25^ As a result, the arising SMM properties are often more stellar compared to other crystal fields around that ion.

The combination of bulky cyclopentadienyl (Cp) ligands with a Dy^III^ ion yielded a plethora of sandwich compounds with notable SMM behavior, some of which feature high *T*_B_.^26−28^ Capitalizing on this success, two anionic five-membered heterocycles such as borolyls, and phosphoyls were ligated to Dy^III^ and afforded SMMs with open hysteresis.^29−31^ Notably, sandwich Dy compounds composed of larger six-membered arene ligands are unknown in the realm of SMMs. However, half sandwich Dy complexes comprising one arene ligand are SMMs but with blocking temperatures with more than a magnitude lower than in the bis(Cp) compounds (Figure S1).^32^

A recent development explores synthetically challenging linear, low-coordinate amide complexes with predicted enhancements in performance characteristics of SMMs, additionally caused by a substantial reduction of undesirable vibrational modes.^33^ Recently, a Dy bis(amide) SMM was achieved where its bent structure engenders highly mixed *M*_J_ states resulting in rapid quantum tunneling which is reflected in hysteresis closed at zero field, Figure 1A.^34^ Dy complexes comprising two or more amide groups and one arene function are rare and exhibit butterfly hysteresis loops, Figure S1.^32,35^ The hitherto best system features a seesaw geometry utilizing a bisanilide terphenyl ligand [LAr]^2–^ with open hysteresis loops up to 5.8 K, Figure 1B.^36^ Taken together the recent advances, we sought to investigate unexplored ligand platforms that are composed of both amide and arene functions, to generate new types of SMMs.

Here, we report the synthesis and characterization of two mononuclear dysprosium(III) compounds bearing triarylamide ligands, (NHAr*)_2_DyCl (**1**), where Ar* = 2,6-(2,4,6-(^i^Pr)_3_C_6_H_2_)C_6_H_3_, and [(NHAr*)_2_Dy][BArF_24_] (**2**). **1** is a neutral molecule with a chloride anion ligated to Dy in the equatorial plane. Treatment of **1** with Tl[BArF_24_] yielded **2** composed of the cationic complex [(NHAr*)_2_Dy]^+^ and the weakly coordinating [BArF_24_]^−^ anion. Both compounds are SMMs with significant magnetic hysteresis under conventional sweep rates. **1** shows butterfly hysteresis below 8 K, whereas **2** exhibits open magnetic hysteresis loops of up to 19.0 K with substantial coercivity of 1.03 T at low temperatures. Notably, both a blocking temperature of *T*_B_ = 19.0 K and a coercive field of *H*_C_ = 1.03 T set a record for both amide- and arene-stabilized SMMs.

The observation of SMM behavior in **1** and **2** is extraordinary considering the classical design principles of maximizing single-ion magnetic anisotropies of oblate-shaped lanthanide ions for mononuclear SMMs. These traditional approaches (a) encapsulate a Dy^III^ ion within as axial as possible coordination sphere and (b) reduce equatorial coordination/interactions to an absolute minimum, both with the aim to maximize the single-ion magnetic anisotropy of the Kramers Dy^III^ ion.^27,28^*Ab initio* calculations on **1** and **2** afforded metrical parameters that match the experimental spin-relaxation barriers and revealed the orientation of the anisotropy axis to be governed by the nitrogen donors. Accordingly, **2** is a rare example of a Dy complex with a crystal field dominated by the amide ligands, resulting in stronger interactions of the metal with the nitrogen donors than with the arene moieties of the triarylamide ligands. This finding will have important ramifications for the development of future design principles that help to discover better performing mononuclear SMMs.

## Results and Discussion

### Synthesis

**Figure 2:**
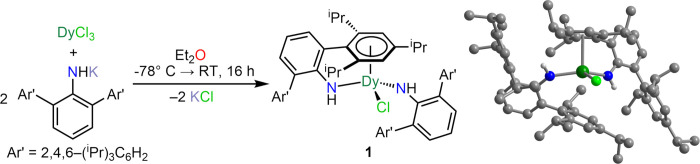
Synthetic scheme and structure for **1**. Dark green, light green, blue, and gray spheres represent Dy, Cl, N, and C atoms, respectively. Pale gray spheres represent H atoms. H atoms bound to all carbon atoms have been omitted for clarity. A depiction of the structure with ellipsoids is shown in Figure S14. Selected interatomic distances (Å) and angles (deg): Dy–N = 2.258(3), 2.235(3); mean Dy–C = 2.905(4); Dy–Cnt = 2.542; Dy–Cl = 2.515(1); Cnt–Dy–N = 98.2, 94.3; Cnt–Dy–Cl = 107.9; N–Dy–N = 140.9(1). Crystallographic data and structural refinement information are provided in Table S2.

**Figure 3:**
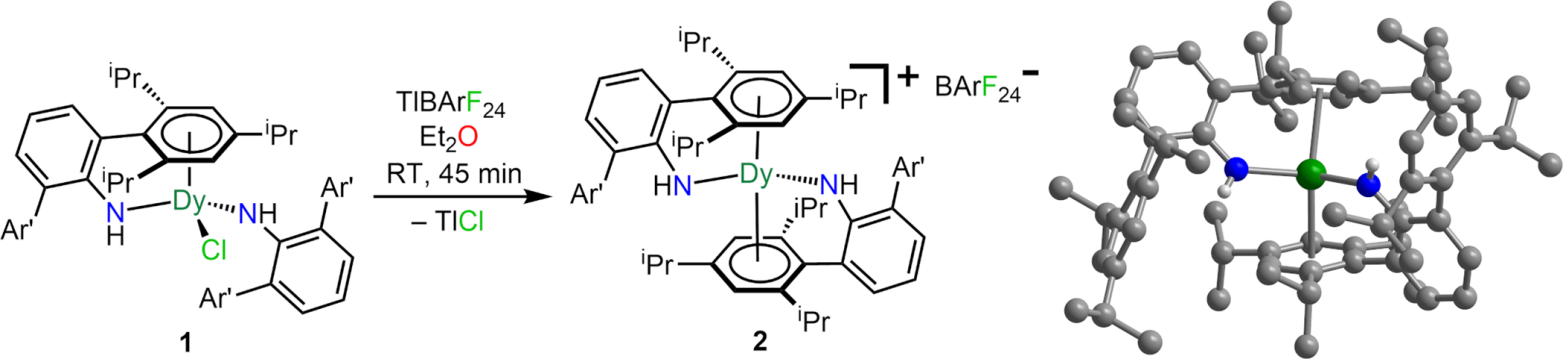
Synthetic scheme for **2**. Structure of the cation [(NHAr*)_2_Dy]^+^ in a crystal of **2**. Green, blue, and gray spheres represent Dy, N, and C atoms, respectively. Pale gray spheres represent H atoms. H atoms bound to all carbon atoms and the [BArF_24_]^−^ anion have been omitted for clarity. A depiction of the structure with ellipsoids is shown in Figure S15. Selected interatomic distances (Å) and angles (deg): Dy–N = 2.222(2); mean Dy–C = 2.864(2); Dy–Cnt = 2.496; Cnt–Dy–N = 98.2, 94.3; Cnt–Dy–Cnt = 152.6; N–Dy–N = 121.7(1). Crystallographic data and structural refinement information are provided in Table S2.

First, the unsolvated potassium triarylamide compound was synthesized in 80% yield from the reaction of KCH_2_SiMe_3_ with H_2_NAr* in *n*-hexane.^37−40^ Second, a salt metathesis reaction of DyCl_3_ with 2 equiv of KNHAr* in diethyl ether afforded the dysprosium chloride complex (NHAr*)_2_DyCl (**1**) and KCl as the byproduct, Figure 2. Storing concentrated *n*-hexane solutions of **1** at–35 °C yielded overnight yellow crystals in 58% yield, suitable for single-crystal X-ray diffraction analysis. Subsequently, a slow dropwise addition of 1 equiv of Tl[BArF_24_] to the chloride complex **1** in diethyl ether resulted in the formation of [(NHAr*)_2_Dy][BArF_24_] (**2**) and TlCl as the byproduct, Figure 3. Orange crystals of **2** suitable for single-crystal X-ray diffraction analysis were obtained overnight from concentrated diethyl ether solutions at–35 °C in 22% yield.

### Structures

#### Compound **1**

Structural analysis revealed a mononuclear dysprosium complex for **1** comprising two triarylamide ligands and one chloride ion ligated to the metal center (Figure 2, Table S1). One of the triarylamide ligands is η^6^-coordinated to the dysprosium ion through the ortho-arene moiety leading to a half sandwich compound. The Dy–Cnt (Cnt = centroid of the arene ring) distance is 2.542 Å, and the closest Dy–C_Ar_ distance amounts to 2.785(4) Å. This agrees well with Dy–Cnt distances of other aryl compounds such as 2.56 Å and 2.476 Å found in [K(DME)_n_][LDyCl_2_] (L = {C_6_H_4_[(2,6-^i^PrC_6_H_3_)NC_6_H_4_]_2_}^2–^) and [(C_7_H_8_)Dy(AlCl_4_)_3_].^36,41^ Compared to the isostructural yttrium complex,^40^ the Dy–Cnt and Dy–C_Ar_ distances in **1** are marginally longer which is ascribed to the slightly larger ionic size of Dy^III^ relative to Y^III^ (Shannon radii for six-coordinate Dy^III^ and Y^III^ ions are 0.912 and 0.900 Å, respectively).^42^ Despite the structural discrepancy in coordination to the metal in solution, it is expected that the arene rings are equal and exhibit fast exchange between aromatic groups, much like observed for the yttrium congener on the timescale of NMR spectroscopic experiments.^40^ The paramagnetic nature of Dy, however, precludes such analogous studies to the same precision (Figure S2).

The Dy–N distances in **1** are 2.258(3) and 2.235(3) Å which are in accordance with corresponding values found in mononuclear Dy compounds, such as 2.379(9)-2.416(8) Å, 2.21(2)/2.20(6) Å, and 2.266(4)/2.288(4) Å, in [K(DME)_n_][LDyCl_2_], (NN^TBS^)DyI(THF)_2_ (NN^TBS^ = (C_5_H_4_{NHSi^t^BuMe_2_})_2_Fe), and [Dy{N(Si^i^Pr_3_)_2_}_2_(BH_4_)], respectively.^32,34,36^ The Dy–N distances fall shorter than the respective distances in dinuclear systems with bridging nitrogen ligands, for instance 2.353/2.368 Å and 2.351(6)/2.388(7) Å in (Cp_2_Dy{μ-NH_2_})_2_ and (Tp_2_Y{μ-NH_2_})_2_ (Tp = hydrotris(1-pyrazolyl)borate), respectively.^43,44^ The Dy–Cl distance in **1** is 2.515(1) Å which is a typical bond length observed in chloride-containing Dy complexes.^26,36,45^

The Cnt–Dy–N angles of 98.2° and 94.3° show less discrepancy to one another than the ones in the yttrium congener (94.4° and 101.8°).^40^ The N–Dy–N angle is 140.9(1)° highlighting substantially bent nitrogen coordination to the metal center perpendicular to the Dy–Cnt axis.

#### Compound **2**

Single-crystal X-ray diffraction studies on **2** uncovered a mononuclear Dy complex that resides on a crystallographic inversion center such that the two nitrogen atoms and coordinating aryl moieties of the triarylamide ligands are equivalent by symmetry (Figure 3, Table S1). Each triarylamide ligand coordinates through an aryl ring in an η^6^ fashion to the metal center, while concurrently being ligated through the amide nitrogen atoms, constituting the cationic part [(NHAr*)_2_Dy]^+^ of **2**. Charge balance is reached through the bulky, weakly coordinating [BArF_24_]^−^ anion. The Dy–Cnt distance is with 2.496 Å approximately 0.05 Å shorter than the corresponding distance in **1**, which can be ascribed to less equatorial steric hindrance due to lack of chloride and additional strain caused by the second coordinating phenyl ring, which was noncoordinating in **1**. Thus, the mean Dy–C distances are also shorter in **2** compared to **1**. A similar impact could be present in the Dy–N distance which is 2.222(2) Å and hence, slightly shorter relative to **1**.

By contrast, the Cnt–Dy–N angles are with 98.2° and 94.3° quite similar to those in **1**. The Cnt–Dy–Cnt angle is 152.6° varying significantly from a 180° linear coordination further attesting that the interaction of Dy^III^ with the carbon atoms of the aryl ring of the triarylamide ligand is disparate. Intriguingly, the N–Dy–N angle in **2** is 121.7(1)° and corresponds to a sizable reduction of 19.2(1)° with respect to **1**. The shortened angle originates from two coordinating aryl rings requiring more space paired with the nitrogen donors positioned on sp^2^ hybridized ipso carbon atoms, rendering them less flexible to rotation. Albeit constituting the largest discrepancy in any angle involving the metal center in view of **1**, this structural disparity will impact the physical properties of **2** as discussed below.

### UV–Visible–NIR Spectroscopy

**Figure 4:**
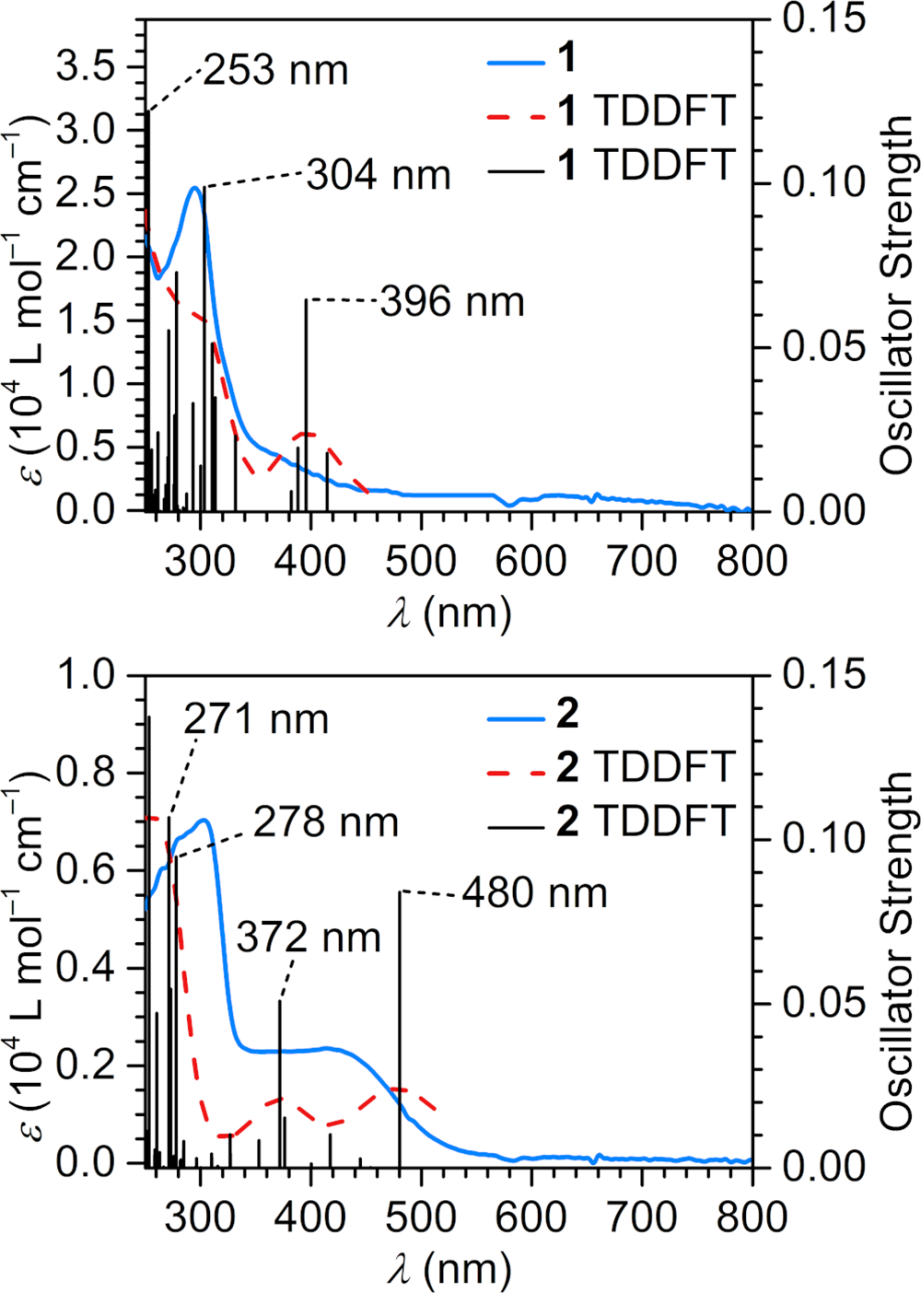
TDDFT-calculated transitions for (NHAr*)_2_DyCl (**1**, top) and [(NHAr*)_2_Dy][BArF_24_] (**2**, bottom) superimposed with experimental spectra. Black bars represent calculated transitions, and dashed red lines represent simulated UV–vis spectra after application of a Gaussian line broadening (0.5 eV full width at half-maximum (FWHM)). See Tables S13 and S14 for full assignment.

The absorption spectra of **1** and **2** were investigated in the UV–vis-NIR region for diluted diethyl ether solutions of **1** (25 μmol/L) and **2** (280 μmol/L) and transitions were analyzed via time-dependent density functional theory (TDDFT) calculations, Figures 4 and S7–S12, Tables S13 and S14. The spectra of **1** feature a single sharp signal at ∼295 nm before steadily decreasing toward higher wavelengths without any additional discernible features in the visible region. This transition aligns well with a calculated transition at 304 nm, which can be associated with a ligand-based transition from the central anilide-based orbital into a π* orbital of the peripheral Ph rings. **2** exhibits a comparably sharp albeit slightly low energy shifted feature at ∼304 nm which decreases in intensity before plateauing between ∼352 nm and ∼383 nm. The higher energy band coincides with two calculated metal-to-ligand charge-transfer transitions at 271/278 nm from anilide-based π orbitals into Dy-based vacant *d* orbitals. The primary acceptor orbital for these transitions also exhibits considerable π* character on the anilide Ph ring. A maximum is found at ∼419 nm, with no further discernible features in the visible region. This band aligns with two calculated intense π → π* transitions at ∼372 nm and ∼480 nm from an anilide-based orbital into π* orbitals of the coordinating Ph rings. Such broad features in the high-energy region of the visible spectrum are found in other arene-capped bis(amide) complexes such as [K(DME)_n_][LDyX_2_] (L = {C_6_H_4_[(2,6-^i^PrC_6_H_3_)NC_6_H_4_]_2_}^2–^, X = Cl, I) (Figure 1B),^36^ and may arise here from ligand-based π → π* transitions as well.

### Magnetism

#### Static Magnetic Susceptibility

**Figure 5:**
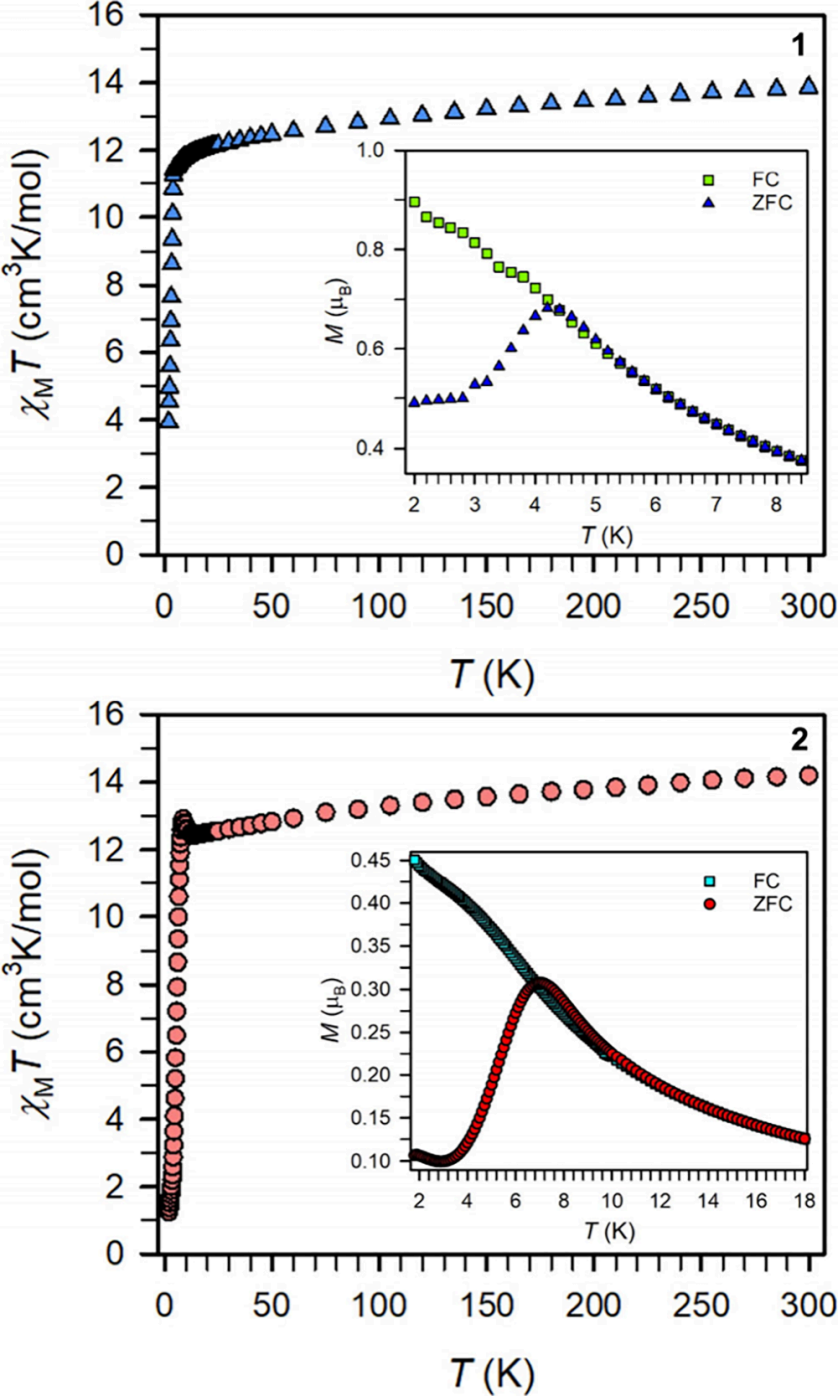
Temperature dependence of the χ_M_*T* product for polycrystalline samples of **1** (top) and **2** (bottom) under a 1000 Oe applied dc field. Insets: Plot of magnetization vs temperature for **1** and **2** during field-cooled (green and turquoise squares, respectively) and zero-field-cooled (blue triangles and red circles, respectively) measurements (**1** was measured under a 1500 Oe dc field, and **2** was measured under a 1000 Oe dc field).

Static magnetic susceptibility measurements were carried out on polycrystalline samples of **1** and **2** between 2 and 300 K under 0.1, 0.5, and 1 T direct current (dc) fields (Figures 5 and S16–S25). Here, the data collected under 0.1 T field will be primarily discussed. At 300 K, the product of molar magnetic susceptibility and temperature (χ_M_*T*) is 13.86 cm^3^ K/mol for **1** and 14.19 cm^3^ K/mol for **2** where each is in excellent agreement with the expected value of 14.17 cm^3^ K/mol for the respective free Dy^III^ ion (Figure 5).

As the temperature is lowered, the χ_M_*T* values decline progressively for **1** reaching 11.48 cm^3^ K/mol at 4.8 K, followed by a precipitous drop in χ_M_*T* to yield a value of 3.94 cm^3^ K/mol at 2 K. By comparison, lowering the temperature for **2**, initiates first a gradual decline until 14.0 K reaching χ_M_*T* of 12.44 cm^3^ K/mol, followed first by a slight surge to 12.91 cm^3^ K/mol at 8.6 K, and then below that temperature a significant drop of χ_M_*T* approaching a value of 1.25 cm^3^ K/mol at 2 K. Noteworthy, the dip in χ_M_*T* at low temperatures is markedly more distinct for **2** relative to **1**.

Such precipitous plummeting behavior of χ_M_*T* at low temperatures is ascribed to magnetic blocking, which refers to the magnetic moments being pinned by the strong magnetic anisotropy, incapable of following an external magnetic field. Consequently, the presence of magnetic blocking signifies the possibility of the molecules showing a magnetic memory effect. This interpretation is corroborated by the divergence of field-cooled (fc) and zero-field-cooled (zfc) magnetic susceptibility measurements which display thermoremanent magnetization (Figure 5, insets). For **1**, the zfc and fc data intersect first at 4.4 K and fully overlap at 5.4 K. For **2**, the zfc and fc data intersect first at 8 K and then fully overlay at 12 K.

#### Dynamic Magnetic Susceptibility

**Figure 6:**
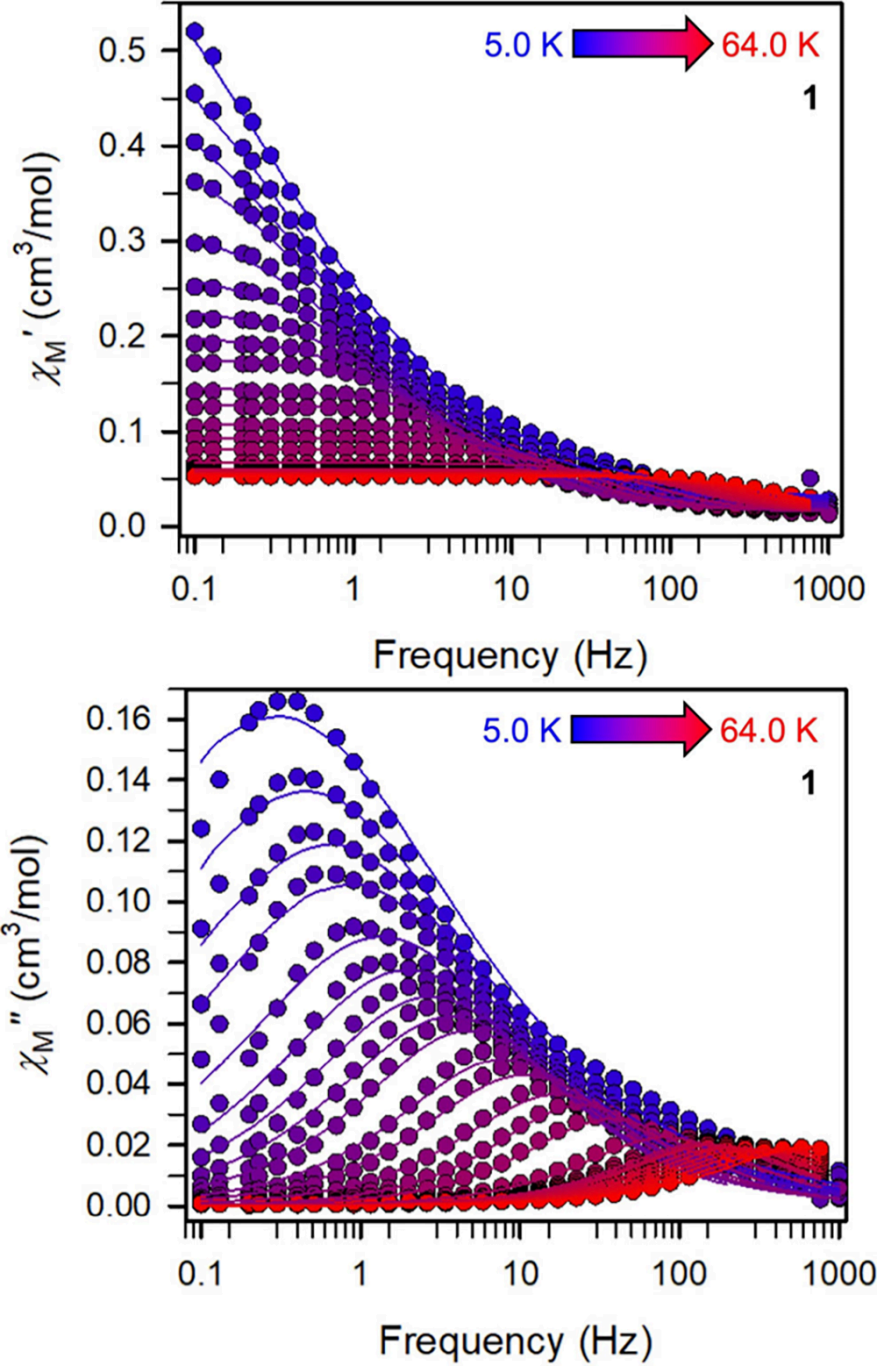
Dynamic magnetic susceptibility data. Variable-temperature, variable-frequency in-phase (χ_M_′) and out-of-phase (χ_M_″) ac magnetic susceptibility data collected under a zero applied dc field for **1** from 5.0 to 64.0 K. Fits of a generalized Debye function to the χ_M_′ and χ_M_″ data afforded the relaxation times, τ. Solid lines represent fits to the data. Data from 2.0 and 64.0 K are shown in Figure S26.

**Figure 7:**
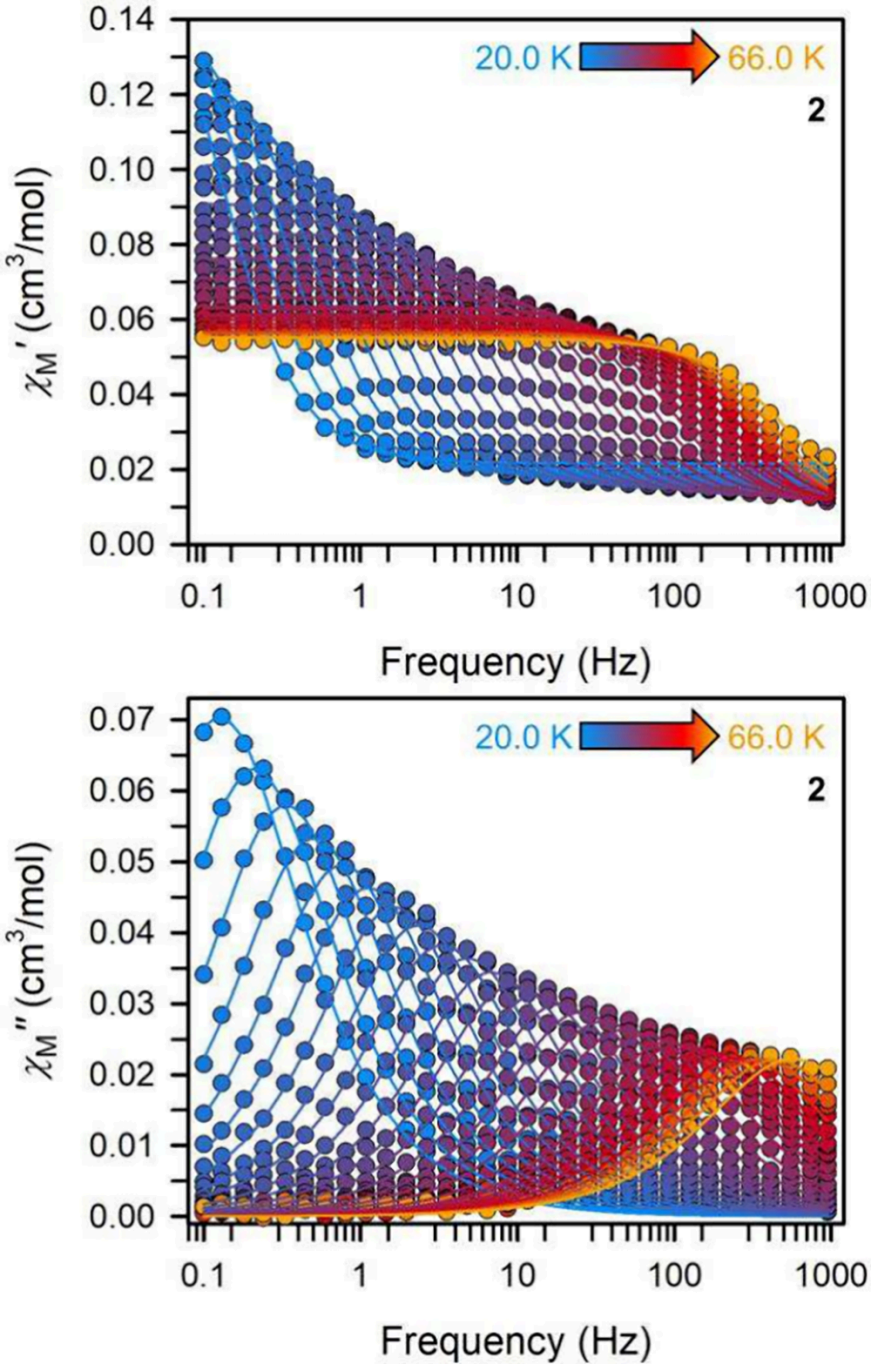
Dynamic magnetic susceptibility data. Variable-temperature, variable-frequency in-phase (χ_M_′) and out-of-phase (χ_M_″) ac magnetic susceptibility data collected under a zero applied dc field for **2** from 20.0 to 66.0 K. Fits of a generalized Debye function to the χ_M_′ and χ_M_″ data afforded the relaxation times, τ Solid lines represent fits to the data.

The magnetization relaxation dynamics of **1** and **2** were probed through variable-temperature, variable-frequency alternating current (ac) magnetic susceptibility measurements. For both compounds, peaks in the out-of-phase magnetic susceptibility (χ_M_″) were observed under zero dc field, suggestive of long magnetic relaxation times (Figures 6, 7, and S26). At 2 K, under ac frequencies spanning from 0.1 to 1000 Hz, **1** exhibited an out-of-phase (χ_M_″) peak maximum at 0.23 Hz, which decreased in intensity with rising temperatures while simultaneously remained steady with respect to frequency (Figure 6). At temperatures above 5 K, the χ_M_″ signal starts to shift to higher frequencies as the temperature is increased, until it eventually moves beyond the frequency limit of the SQUID magnetometer at 64.0 K. By contrast, exposing **2** to an ac magnetic field of 3 Oe at oscillating frequencies of 0.1 to 1000 Hz at temperatures between 20 and 66 K, initiated the maximum of the χ_M_″ signal to change frequency over the entire investigated temperature range (Figure 7).

Notably, the known mononuclear dysprosium SMMs with a crystal field composed of six-membered arene ligands coordinating to the metal ion, such as [(C_6_Me_6_)Dy(AlCl_4_)_3_] and [(C_7_H_8_)Dy(AlX_4_)_3_] (X = Cl, Br), exhibit faster magnetic relaxation dynamics on the timescale of ac magnetic susceptibility measurements.^41,46^ The highest reported *U*_eff_ for a bent Dy bis(amide) complex is 591 cm^–1^, but features substantial quantum tunneling at the lowest temperatures with invariant out-of-phase (χ_M_″) peaks with respect to frequency between 2 to 10 K.^34^

**Figure 8:**
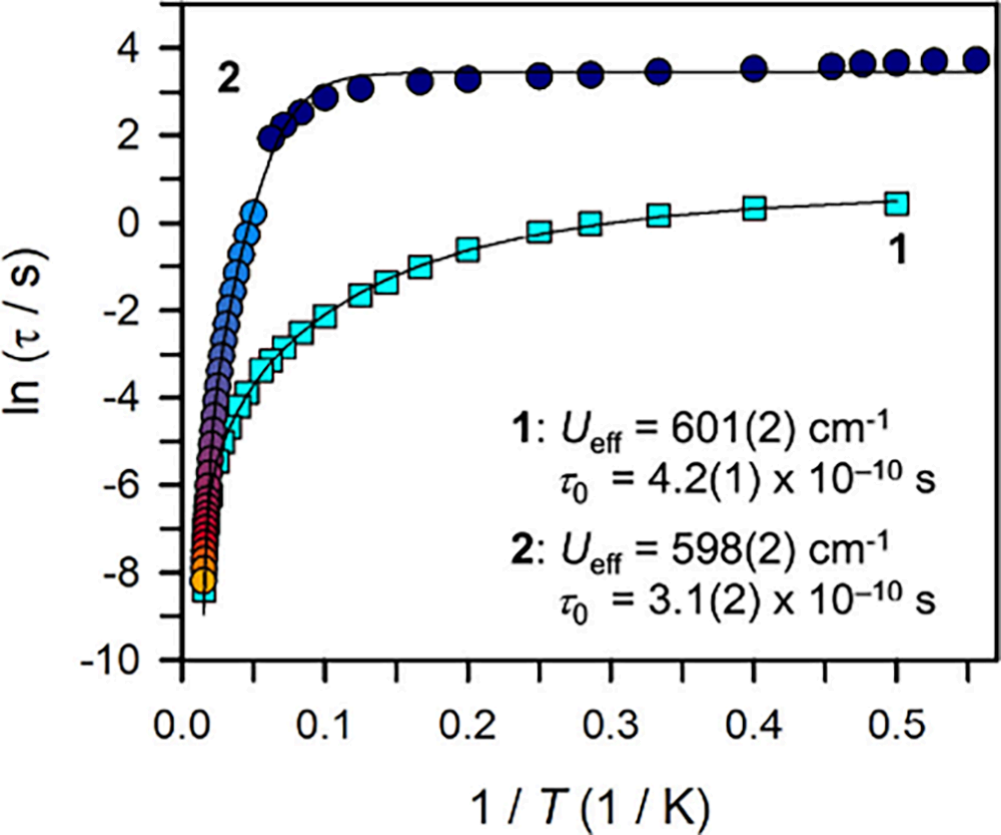
Plot of natural log of the relaxation time versus the inverse temperature for **1** (temperature range 2 to 64 K) and for **2** (temperature range 1.8 to 66 K). For **1**: Turquoise squares represent data extracted from ac magnetic susceptibility measurements. For **2**: Pale blue to orange-red circles represent data extracted from ac magnetic susceptibility measurements, and dark blue circles represent data extracted from dc relaxation experiments (temperature range 1.8 to 16 K). Each black line represents a fit to a Orbach relaxation process, a Raman relaxation process, and a quantum tunneling pathway, as described in the main text. Individual contributions to these fits are shown in the Supporting Information.

A quantitative assessment of magnetic relaxation times (τ) for both compounds as a function of temperature was carried out via the generation of Cole–Cole plots for each temperature, followed by fitting of the plots to a generalized Debye model (Figures S27–S29). The dependence of τ on temperature reveals the type of magnetic relaxation processes operative at certain temperatures for a respective system. The Arrhenius plots feature curved τ for **1** and **2**, implying that the moments have entry to multiple relaxation pathways for spin relaxation, ultimately eliciting relaxation times τ with differing temperature dependences. Hence, τ were fit to multiple relaxation processes (Figures 8, S30, and S31), to determine their contributions to the observed relaxation rates. In fact, we probed several fits employing eq 1 which contains the most common magnetic relaxation pathways, affording the best-fit parameters listed in Table 1.
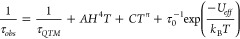
1Here, the first term is for the temperature independent quantum tunneling of the magnetization (abbreviated as QTM) process, the second term is for the direct process (∝*T*),^47^ the third term is for the Raman relaxation process (∝*T*^*n*^, *n* typically varies in value but does not exceed 9),^47^ and the fourth term models the Orbach relaxation process (∝exp(*U*_eff_/k_B_*T*)).^48−50^ Importantly, for successful modeling of the Arrhenius plots for **1** and **2** each term was not necessary, and hinging on the circumstances of the measurement some were judiciously excluded from the fits. Most notably, the ac magnetic susceptibility measurements on **1** and **2** were conducted at zero dc field, and thus, that data were not modeled with the direct process since the corresponding contribution is nullified in the absence of a dc field.

**Table 1:** Best-Fit Parameters for the Arrhenius Plots of **1** and **2** under a 0 Oe Applied dc Field

Compound	τ_QTM_ (s)	*C* (s^–1^ K^–n^)	*n*	τ_0_ (× 10^–10^ s)	*U*_eff_ (cm^–1^)
**1**^a^	2.2350	0.02952	2.41	4.2(1)	601(2)
**2**^a^	8.0822	1.0 × 10^–8^	6.01	8.6(2)	600(1)
**2**^a^		1.74 × 10^–8^	5.85	6.8(2)	591(3)
**2**^b^	31.8317	1.65 × 10^–8^	5.85	3.1(2)	598(2)

a Data extracted from ac susceptibility measurements.

b Data extracted from ac and dc susceptibility measurements.

For **1**, the curved τ values between 2 and 64 K were successfully modeled by considering QTM, Raman, and Orbach relaxation processes, affording an effective spin-reversal barrier *U*_eff_ of 601(2) cm^–1^ and an attempt time τ_0_ of 4.2(1) × 10^–10^ s. The τ_0_ magnitude is in line with values found for other mononuclear Dy SMMs.^26,28,30,31,51,52^ Notably, the exclusion of a QTM process yielded unsatisfactory fits, revealing that the magnetic relaxation occurs through a quantum tunneling pathway at the lowest temperatures. This interpretation is in line with the χ_M_″ peak position between 2 and 5 K remaining largely invariant with respect to frequency (Figure S26).

By contrast, successful modeling of τ for **2** between 20 and 66 K required application of Raman and Orbach relaxation processes, yielding *U*_eff_ = 591(3) cm^–1^ and τ_0_ = 6.8(2) × 10^–10^ s (Figure S32). The magnitude for τ_0_ in **2** agrees well with values of other mononuclear dysprosium SMMs.^26,28,30,31,51,52^ An inclusion of a QTM process in addition to the Orbach and Raman processes slightly improved the quality of the fit and afforded values of *U*_eff_ = 600(1) cm^–1^ and τ_0_ = 8.6(2) × 10^–10^ s, with a tiny quantum tunneling term of τ_QTM_ = 8 s, further attesting a miniscule presence of QTM in the probed temperature range (Figure S31). This is expected and in accordance with the results from the static magnetic experiments revealing magnetic blocking to occur below 20 K.

#### Dc Relaxation Experiments

**Figure 9:**
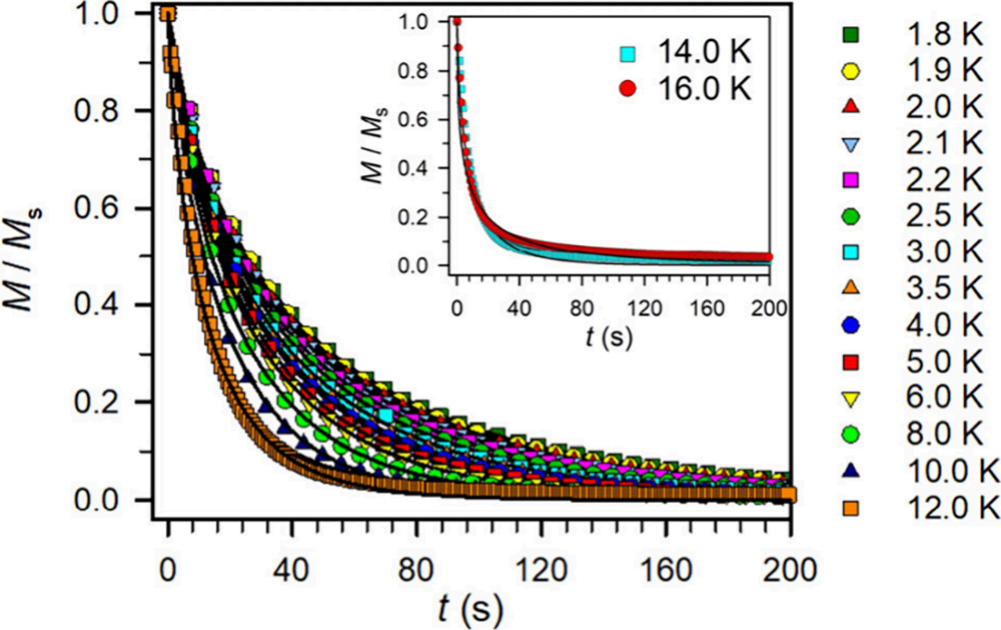
Plot of magnetization (normalized) vs time used to derive relaxation times for **2** at different temperatures from 1.8 to 12 K (inset: 14 and 16 K, shown separately for clarity). The data were fit to a function of the form *y* = *a*·exp(−((*t*/τ)*^b^*)) where *b* is a stretch factor (black line). Decay of the magnetization vs time for **2** was obtained by applying a magnetic field of 4 T to the sample at a given temperature for 5 min and then by quickly removing the magnetic field. The full data (unnormalized) recorded at each temperature are shown in Figures S34–S49.

To gain a full understanding of all operative relaxation processes, including those inaccessible at low temperatures due to the limits of ac magnetic susceptibility techniques, elaborate dc relaxation experiments were performed at several temperatures within the range of 1.8 to 16 K (Figure 9). In this approach, at a given temperature, first, a high dc magnetic field is applied to the sample to attain magnetic saturation. Second, the dc field is removed as quickly as possible. Third, after reaching zero field, the magnetic relaxation is recorded which will obey an exponential dependence. Accordingly, τ at 16 temperatures spanning from 1.8 to 16 K were extracted through dc relaxation measurements and added to the Arrhenius plot (Figures 8, S34–S53 and Table S4). Taking into account the dc relaxation and ac magnetic susceptibility experiments, the τ values expanded to a range from 1.8 to 66 K and required for successful modeling the inclusion of a QTM process, in addition to Orbach and Raman relaxation processes (Figures S52 and S53). A satisfactory fit afforded *U*_eff_ = 598(2) cm^–1^ and τ_0_ = 3.1(2) × 10^–10^ s.

Consideration of 1σ uncertainty ranges via a log-normal model for τ values through the CCFIT2 program obtained from ac measurements, as well as α- and β- distributions for ac- and dc-derived τ values,^53,54^ yielded slightly higher *U*_eff_ values of 626(31) cm^–1^ for **1** and 625(2) cm^–1^ for **2** (Figures S33 and S54, Tables S3, S5, and S6).

#### Magnetic Hysteresis

Variable-field magnetization data were collected for polycrystalline samples of **1** and **2** between ±7 T using an average sweep rate of 0.01 T s^–1^ (Figures 10 and S55–S74). The hysteresis loops measured for **1** between 1.8 and 8.0 K are all waist-constricted and thus, closed at zero field, Figures 10 and S55–S62. At higher fields, the hysteresis loops open to a butterfly shape below 8 K. At 8 K, the hysteresis curves are fully closed. This hysteretic behavior suggests the presence of strong QTM, which may originate from the equatorially coordinated chloride ion introducing transverse magnetic anisotropy.

**Figure 10:**
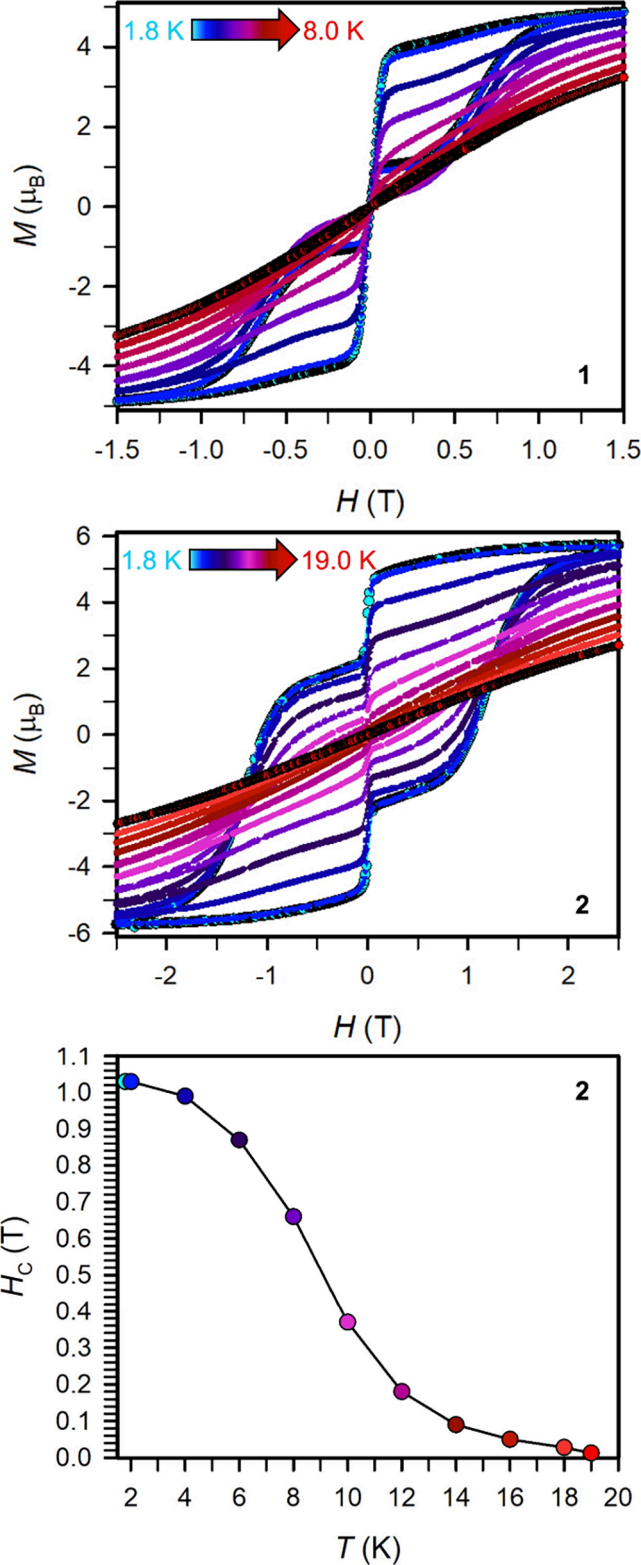
Variable-field magnetization (*M*) data collected for **1** (top) and **2** (middle) at a sweep rate of 100 Oe/s, respectively. Plot of coercive field vs temperature for **2** (bottom). Solid lines are guides for the eye.

By contrast, the hysteresis loops measured for **2** are open at zero field below 19.0 K (Figures 10, S63–S74). At 1.8 K, the coercive field is *H*_C_ = 1.03 T which is the largest found for a mononuclear dysprosium complex with arene or amide ligands in the first coordination sphere (Figure S63). By the same token, the magnetic blocking temperature of 19.0 K is also the highest observed for an arene- and amide-stabilized Dy^III^ cation, respectively. The value for *H*_C_ starts slowly decreasing starting at 4 K (*H*_C_ = 0.99 T at 4 K). When the temperature is further raised, a gradual decrease of *H*_C_ occurs which culminates in full closure of the hysteresis loop at 19.0 K (Figure S74). The steady decrease of the coercive field, *H*_C_, as a function of increasing temperature is depicted in Figure 10.

Noteworthy, the hysteresis loops display distinct steps at *H*_C_ = 0 Oe, suggestive of operative quantum tunneling pathways in **2**. Such pronounced steps have been ascribed to QTM in many cases.^27,35,55^ From the shape of the hysteresis, it is apparent that slower relaxation processes take over once an applied field disrupts the tunneling, which opens the loops. Evidence of temperature-independent relaxation through QTM is not seen in **2** through the ac magnetic susceptibility measurements that are carried out on much faster timescales.

**2** features the highest blocking temperature of *T*_B_ = 19.0 K for a mononuclear Dy complex that is stabilized by two amide ligands. Importantly, the fact that the hysteresis observed for **2** is open, outperforms all mononuclear Dy SMMs with amide or arene ligands, or a combination of both, which generally exhibit butterfly hysteresis that are closed at zero field (equaling to *H*_C_ = 0) and open at higher fields.^18,34−36,41,46,56^ The maximum coercive field of *H*_C_ = 1.03 T in **2** is massive within the realm of amide-stabilized Dy complexes, and resides among the best coercive fields observed for mononuclear nonmetallocene Dy SMMs such as *H*_c_ of 0.73 T observed for the dysprosium complex DyL_2_I (HL = 2-({2,6-dibenzhydryl-4-iso-propylphenylimino}methyl)-4,6-di*tert*-butylphenol).^13^ The *H*_c_ for **2** is also exceeding systems that exhibit maximum axiality and are innate to a weak equatorial coordination sphere for the metal ion such as [Dy(L^N6^)(Ph_3_SiO)_2_][BPh_4_] (where L^N6^ = (1*R*,2*R*)-1,2-bis(2,4,6-trifluorophenyl)-ethane-1,2-diamine), for which *H*_c_ was measured to be 0.62 T at 2 K.^57^ We note that these literature examples used faster sweep rates, precluding a direct comparison to **1** and **2**.

Field-dependent magnetization measurements (*M* vs *H*) on **1** and **2** were performed between 0–7 T and 2 and 10 K (Figures S75 and S77). The *M* vs *H* curves of both **1** and **2** exhibit characteristics of magnetic blocking at low temperatures. For **1**, at low fields, the magnetization rises to a value of 0.39 μ_B_ at 600 Oe, and subsequently increases slower to 0.78 μ_B_ at 1900 Oe before accelerating again and quickly climbing to 4.99 μ_B_ at 1 T. Subsequently, the magnetization gradually increases to the maximum value of 5.90 μ_B_ at 7 T without reaching full saturation. This maximum *M* value and shape of the low temperature *M* vs *H* curves are in excellent agreement with other axial Dy^III^ complexes such as 5.60 μ_B_ in [Dy(L^S^)(4-Me-PhO)_2_](BPh_4_) (L^S^ is a chiral macrocycle derived from 2,6-diformyl-pyridine and (1*R*,2*R*)/(1*S*,2*S*)-1,2-diphenylethylenediamine), where the S-shape is associated with magnetic blocking at low temperatures.^55^ Above 4 K, **1** exhibits a continuous *M* vs *H* curve.

For **2**, a similar to **1** but more pronounced S-shape is observed: The initial sharp rise to 0.16 μ_B_ at 400 Oe is followed by a slower increase to 1.25 μ_B_ at 5900 Oe, which then transitions into a second sharp ascent to 5.31 μ_B_ at 1.41 T. The *M* is not saturated even at the highest applied fields, reaching the maximum *M* value of 6.08 μ_B_ at 7 T. This value compares well to **1** and the only other cationic, solvent-free Dy bis(amide) complex [{N(Si^i^Pr_3_)_2_}_2_Dy][Al{OC(CF_3_)_3_}_4_], which exhibits *M*_sat_ = 5.42 μ_B_ at 2 K.^34^ In **2**, the S-shape of the magnetization curves persists up to 8 K, eluding to the considerably augmented magnetic anisotropy achieved through the chloride removal of **1**. This boost in anisotropy is also reflected in the reduced magnetization curves (*M* vs *H*/*T*): While the curves for both **1** and **2** are nonsuperimposable at low temperatures, they are essentially overlapping at 4 K for **1** (Figure S76). By contrast, for **2** these curves remain nonsuperimposable up to 8 K (Figure S78).

### *Ab Initio* Calculations

**1** and **2** exhibit remarkable uniaxiality which prompted us to study their electronic structures via *ab initio* calculations using a complete active space self-consistent field (CASSCF)/N-electron valence perturbation theory (NEVPT2) approach with the Orca 5.0.4 software suite,^58,59^ accounting for the multiconfigurational nature of the Dy^III^ ion (Tables S7–S12).

**Figure 11:**
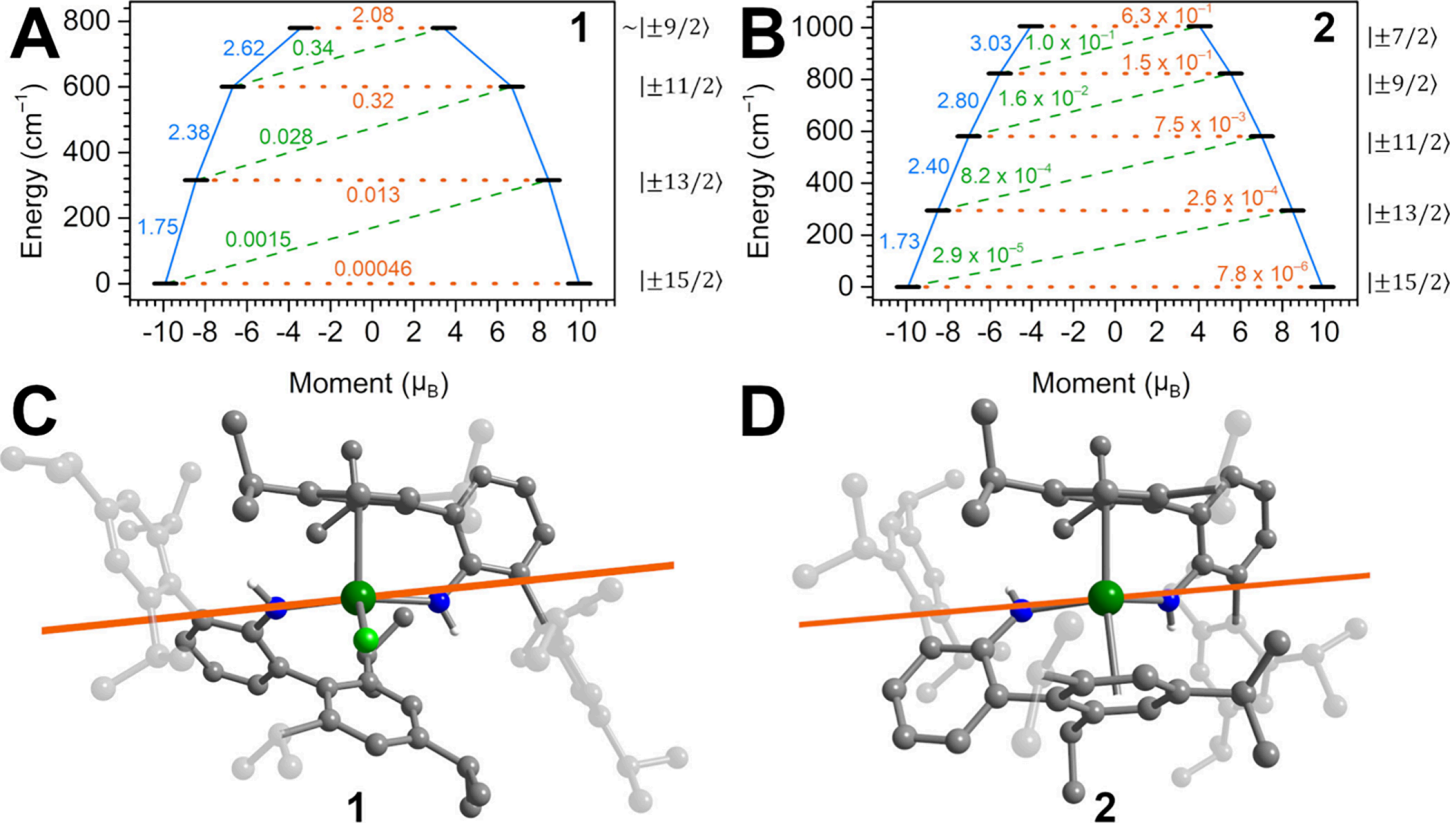
(Top) Estimated relaxation barrier comprising the five lowest-lying Kramers doublets with relaxation pathways shown for (NHAr*)_2_DyCl (**1**) and the [(NHAr*)_2_Dy]^+^ cation in **2**. Solid blue and dashed green lines indicate Orbach and/or Raman processes. Orange dotted lines indicate quantum tunneling (QTM) or thermally activated QTM pathways. Values next to the arrows correspond to the respective transition magnetic moment matrix elements. The numbers on the right are given for the primary *M*_J_ state comprising the wave function for each state. (Bottom) Plot of the *g*_*z*_-tensor component calculated for the |±15/2⟩ ground state Kramers doublet of **1** and the [(NHAr*)_2_Dy]^+^ cation in **2** with 19.8237 (*g*_*z*_) (**1**) and 19.8212 (*g*_*z*_) (**2**), respectively. See Figures S79 and S80 for the full relaxation barriers and Tables S11 and S12 for average transition dipole moments.

For **1**, the ground Kramers doublet (KD) exhibits a strong axial character (Figure 11, Table S7). Importantly, the uniaxiality of this KD is determined through the monoanionic terminal amide ligands. Disruptions to uniaxiality become non-negligible with access of the third KD at 601 cm^–1^, where the maximum equatorial *g*-tensor contribution is 9% of the *g*_*z*_ value. Consequently, the calculated transition magnetic dipole moments connecting the KD ± 3 states are close to 0.1, which is typically regarded as the critical value for quantum tunneling processes to shortcut a given energy barrier to spin reversal (Table S11).^60,61^ Notably, the calculated KD3 energy matches perfectly with the experimentally determined *U*_eff_ of 601(2) cm^–1^.

The low-lying energy spectrum of **1** can be contextualized through comparison to the only other dysprosium complex featuring a phenyl-capped bis(amide) coordination sphere, [K(DME)_n_][LDyCl_2_] (L = {C_6_H_4_[(2,6-^i^PrC_6_H_3_)NC_6_H_4_]_2_}^2–^, four independent molecules), featuring a seesaw coordination geometry including two equatorially coordinated chloride ligands (Figure 1).^36^ In this complex, substantial equatorial *g* components were also found in the second excited states, which span from 567 to 630 cm^–1^, and the experimental *U*_eff_ value is correlated to the energetically higher third excited state. These KD3 energies encase the calculated value for **1**, which is remarkable for two reasons compared to the reference compound: first, **1** exhibits only one chloride ligand and second, the N–Dy–N angle in **1** is less axial with 140.9(1)° (vs 156.2° for the smallest angle among the four independent molecules).^36^

At first glance, the removal of the chloride ligand from **1** and associated ligation of a second ortho aryl ring of the NHAr* ligand to afford **2**, affects only slightly the electronic structure of the Dy^III^ ion as the ground state KD is strongly axial and determined by the amide N atoms (Figure 11, Table S8). However, the excited KDs in **2** are significantly less mixed relative to **1** and remain essentially pure until the third excited state KD4 at 723 cm^–1^. Here, the admixture |±5/2⟩ into the predominant |±9/2⟩ state induces considerable transverse magnetic fields as indicated by non-negligible *g*_*x*_/*g*_*y*_ components. Remarkably, the KD4 energies of **1** and **2** are nearly identical (**1**: 780 cm^–1^), hinting at a compensatory effect of the change in coordination sphere upon chloride removal and phenyl ring coordination. This is especially striking with respect to the 12.2° reduction in the N–Dy–N angle in **2** (121.7(1)°) compared to **1** (140.9(1)°). While our calculations suggest an increase of ∼120 cm^–1^ in *U*_eff_ upon chloride removal from **1** to **2**, the experimentally extracted *U*_eff_ values for both compounds are similar.

By contrast, field-dependent magnetization experiments clearly show that the chloride removal and aryl coordination mitigate QTM resulting in open hysteresis loops below 19.0 K and a remarkable coercivity of 1.03 T for **2**. Such discrepancies between calculated and measured relaxation barriers have been observed for other dysprosium bis(amide) complexes before.^32,34,36^ More elaborate calculations on **2** implementing spin-phonon couplings may aid uncover a more holistic description of all contributions of the operative relaxation mechanisms.^62−65^

A comparison of the [(NHAr*)_2_Dy]^+^ cation in **2** to the [{N(Si^i^Pr_3_)_2_}_2_Dy]^+^ cation in the only reported “pure” dysprosium bis(amide) complex, [{N(Si^i^Pr_3_)_2_}_2_Dy][Al{OC(CF_3_)_3_}_4_] (Figure 1),^34^ reveals a very similar N–Dy–N angle of 128.7(2)° relative to **2** (121.7(1)°). The Dy–N distance of 2.222(2) Å in **2** is approximately equivalent to the 2.206(5) Å found in the [{N(Si^i^Pr_3_)_2_}_2_Dy]^+^ cation. While the Dy^III^ ion in **2** interacts with aryl groups of the triarylamide (NAr*) ligands in the equatorial plane, the Dy^III^ ion in [{N(Si^i^Pr_3_)_2_}_2_Dy]^+^ exhibits multiple interactions with the isopropyl groups. The weaker Dy–^i^Pr interactions in [{N(Si^i^Pr_3_)_2_}_2_Dy]^+^ engender a larger splitting of the low-lying energy spectrum relative to **2**, where the second excited KD3 at 823.5 cm^–1^ exhibits a considerable state admixture. Despite its reduced axiality and lower excited state manifold splitting, **2** shows open hysteresis below 19 K with substantial coercivity contrary to the butterfly hysteresis observed in [{N(Si^i^Pr_3_)_2_}_2_Dy][Al{OC(CF_3_)_3_}_4_].^34^ It can be hypothesized that the increased rigidity of the arene rings over methyl groups in close proximity to the Dy^III^ ion in **2** prevents unfavorable low-energy molecular vibrations that could speed up magnetic relaxation.

Lastly, to validate our calculations, we predicted the χ_M_*T* vs *T* curves at 0.1 T of **1** and **2** (Figures S81 and S82). For **1**, starting at room temperature, the calculated χ_M_*T* vs *T* values decline gradually upon decreasing temperatures and closely follow the experimental zfc dc data before dropping precipitously at around ∼6 K. Similarly, the calculated χ_M_*T* vs *T* values for **2** decline gradually upon decreasing temperatures and closely follow the experimental profile, but start deviating below 20 K due to pronounced magnetic blocking and above-mentioned limitations of the theoretical approach chosen in this study.

## Conclusions

Two new mononuclear dysprosium(III) complexes were synthesized and in-depth characterized involving crystallography, spectroscopy, magnetometry, and *ab initio* calculations. First, the half-sandwich complex (NHAr*)_2_DyCl (**1**) was synthesized from DyCl_3_ and KNHAr*. Second, **1** was treated with Tl[BArF_24_] to give the sandwich complex [(NHAr*)_2_Dy][BArF_24_] (**2**) under extrusion of the byproduct TlCl. The cationic part of **2** is notable as it comprises the first isolated Dy^III^ ion that is sandwiched by two anionic amide ligands where the coordination occurs through the nitrogen donors concurrent with the interaction to two aryl moieties. The magnetic properties of **1** and **2** are majorly governed by the orientation of the magnetic anisotropy axis which runs through the nitrogens of the triarylamide ligands. This may originate from the largest anionic charge residing on the nitrogen atoms rather than on the coordinating phenyl moieties, which may be augmented by the nitrogen atoms being more electronegative. The chloride removal and bis(aryl) coordination to the metal ion suppresses substantially QTM leading to open hysteresis loops with a maximum coercive field of 1.03 T for **2**. The magnetic hysteresis for **2** constitutes a record in two ways for amide- or arene-stabilized SMMs: 1. Magnetic hysteresis is observed until 19.0 K which is more than twice as high as the previous record; 2. For the first time, the magnetic hysteresis loops are open at zero field. Intriguingly, the effective barriers to spin relaxation for both complexes are of similar magnitude. Thus, moving the coordinating aryl rings further away from the Kramers Dy^III^ ion through judicious ligand design, may afford better performing SMMs. In sum, the foregoing results have huge ramifications for a unique synthetic access to high-blocking mononuclear SMMs implementing new types of ligands.

## Experimental Section

All experimental procedures were shown in Supporting Information, including synthesis methods, crystallographic measurements, spectroscopic measurements, magnetic measurements, and computational methodology.

## Supporting Information Available

Experimental Synthetic details; crystallographic details; molecular structures and bond lengths/angles; UV–vis–NIR spectra; magnetic measurements; calculation details (PDF)
