# Supplementary material for: Magnetic Hysteresis in a Dysprosium Bis(amide) Complex
Source: J Am Chem Soc. 2025 Feb 27;147(10):8156–67. doi: 10.1021/jacs.4c08137 (PMC11912338; doi:10.1021/jacs.4c08137)
Supplement: Supplementary file 1 — ja4c08137_si_001.pdf [file ja4c08137_si_001.pdf]

Supporting Information

for

Magnetic Hysteresis in a Dysprosium Bis(amide) Complex

Florian Benner, Rashmi Jena, Aaron L. Odom*, Selvan Demir*

Department of Chemistry, Michigan State University, 578 South Shaw Lane, East Lansing, MI 48824, USA.

*Email: odom@chemistry.msu.edu; sdemir@chemistry.msu.edu

J. Am. Chem. Soc.

Table Of Contents

**1 General Considerations**	**S6**
**2 Literature Examples of Dy Complexes**	**S10**
**Figure S1.** Literature examples of arene and amide complexes used for SMM studies.	**S10**
**3 NMR Spectroscopy**	**S11**
**Figure S2.** ^{1}H NMR spectrum of $(NHAr^*)_2DyCl$ (**1**) in C_6D_6 at room temperature.	**S11**
**Figure S3.** ^{1}H NMR spectrum of $[(NHAr^*)_2Dy][BArF_{24}]$ (**2**) in THF-d_8 at room temperature	**S12**
**Figure S4.** ^{1}H NMR spectrum (zoomed in) of $[(NHAr^*)_2Dy][BArF_{24}]$ (**2**) in THF-d_8 at room temperature.	**S13**
**Figure S5.** ^{13}C NMR spectrum of $[(NHAr^*)_2Dy][BArF_{24}]$ (**2**) in THF-d_8 at room temperature.	**S14**
**Figure S6.** ^{19}F NMR spectrum of $[(NHAr^*)_2Dy][BArF_{24}]$ (**2**) in THF-d_8 at room temperature.	**S15**
**4 UV-vis-NIR Spectroscopy**	**S16**
**Figure S7.** Vis-NIR spectrum of $(NHAr^*)_2DyCl$ (**1**) obtained from 25 μM solution in diethyl ether.	**S16**
**Figure S8.** UV-vis spectrum of $(NHAr^*)_2DyCl$ (**1**) obtained from serial dilution of 100 μM solution.	**S16**
**Figure S9.** Concentration vs absorbance plot to calculate molar extinction coefficient at 297 nm for $(NHAr^*)_2DyCl$ (**1**) in diethyl ether.	**S17**
**Figure S10.** Vis-NIR spectrum of $[(NHAr^*)_2Dy][BArF_{24}]$ (**2**) obtained from 25 μM solution in diethyl ether.	**S17**
**Figure S11.** UV-vis spectrum of $[(NHAr^*)_2Dy][BArF_{24}]$ (**2**) obtained from serial dilution of 570 μM solution.	**S18**
**Figure S12.** Concentration vs absorbance plot to calculate molar extinction coefficient at 415 nm for $[(NHAr^*)_2Dy][BArF_{24}]$ (**2**) in diethyl ether.	**S18**
**5 FT-IR Spectroscopy**	**S19**
**Figure S13.** IR spectra of $(NHAr^*)_2DyCl$ (**1**) and $[(NHAr^*)_2Dy][BArF_{24}]$ (**2**) recorded on polycrystalline solids	**S19**
**6 Single Crystal X-ray Diffraction**	**S20**
**Figure S14.** Structure of $(NHAr^*)_2DyCl$ (**1**).	**S20**
**Figure S15.** Structure of the cation $[(NHAr^*)_2Dy]^+$ in a crystal of **2**.	**S21**
**Table S1.** Metric data from the crystal structures of $(NHAr^*)_2DyCl$ (**1**) and $[(NHAr^*)_2Dy][BArF_{24}]$ (**2**).	**S22**
**Table S2.** Crystallographic data and structural refinement of $(NHAr^*)_2DyCl$ (**1**) and $[(NHAr^*)_2Dy][BArF_{24}]$ (**2**).	**S23**

7 Magnetic Data S25

Figure S16. Temperature dependence of the $\chi_M T$ product for polycrystalline samples of **1** and **2** under a 0.1 T applied dc field. S25

Figure S17. Temperature dependence of the $\chi_M T$ product for polycrystalline samples of **1** and **2** under a 0.1 T applied dc field. S26

Figure S18. Variable-temperature dc magnetic susceptibility data for a restrained polycrystalline sample of **1** collected under a 0.1 T applied dc field. S26

Figure S19. Variable-temperature dc magnetic susceptibility data for a restrained polycrystalline sample of **1** collected under a 0.5 T applied dc field. S27

Figure S20. Variable-temperature dc magnetic susceptibility data for a restrained polycrystalline sample of **1** collected under a 1 T applied dc field. S27

Figure S21. Variable-temperature dc magnetic susceptibility data for a restrained polycrystalline sample of **1** collected under 0.1 T, 0.5 T, and 1.0 T applied dc fields. S28

Figure S22. Variable-temperature dc magnetic susceptibility data for a restrained polycrystalline sample of **2** collected under a 0.1 T applied dc field. S28

Figure S23. Variable-temperature dc magnetic susceptibility data for a restrained polycrystalline sample of **2** collected under a 0.5 T applied dc field. S29

Figure S24. Variable-temperature dc magnetic susceptibility data for a restrained polycrystalline sample of **2** collected under a 1.0 T applied dc field. S29

Figure S25. Variable-temperature dc magnetic susceptibility data for a restrained polycrystalline sample of **2** collected under 0.1 T, 0.5 T, and 1.0 T applied dc fields. S30

Figure S26. Variable-temperature, variable-frequency in-phase (χ_M') and out-of-phase (χ_M") ac magnetic susceptibility data collected for **1** under a zero applied dc field from 2.0 to 64.0 K. S31

Figure S27. Cole-Cole (Argand) plots for ac susceptibility collected from 2.0 to 64.0 K under a zero applied dc field for **1**. S32

Figure S28. Cole-Cole (Argand) plots for ac susceptibility collected from 5.0 to 64.0 K under a zero applied dc field for **1**. S33

Figure S29. Cole-Cole (Argand) plots for ac susceptibility collected from 20.0 to 66.0 K under a zero applied dc field for **2**. S34

Figure S30. Individual contributions of the multiple magnetic relaxation pathways to the Arrhenius plot of **1** at 0 Oe. S35

Figure S31. Individual contributions of the multiple magnetic relaxation pathways (QTM, Raman, Orbach) to the Arrhenius plot of **2** at 0 Oe and plot of natural log of the relaxation time versus the inverse temperature (temperature range 20 to 66 K) for **2**. S36

Figure S32. Individual contributions of the multiple magnetic relaxation pathways (Raman, Orbach) to the Arrhenius plot of **2** at 0 Oe and plot of natural log of the relaxation time versus the inverse temperature (temperature range 20 to 66 K) for **2**. S37

Table S3. Summary of relaxations times τ of $(NHAr^*)_2DyCl$ (**1**) with upper- and lower τ bounds calculated according to a 1σ model. S38

Figure S33. Individual contributions of the multiple magnetic relaxation pathways to the Arrhenius plot of $(NHAr^*)_2DyCl$ (**1**) at 0 Oe with errors to a 1σ limit. **S39**
Figure S34. Plot of magnetization vs. time used to derive relaxation times for **2** at 1.8 K. **S40**
Figure S35. Plot of magnetization vs. time used to derive relaxation times for **2** at 1.9 K. **S41**
Figure S36. Plot of magnetization vs. time used to derive relaxation times for **2** at 2.0 K. **S42**
Figure S37. Plot of magnetization vs. time used to derive relaxation times for **2** at 2.1 K. **S43**
Figure S38. Plot of magnetization vs. time used to derive relaxation times for **2** at 2.2 K. **S44**
Figure S39. Plot of magnetization vs. time used to derive relaxation times for **2** at 2.5 K. **S45**
Figure S40. Plot of magnetization vs. time used to derive relaxation times for **2** at 3.0 K. **S46**
Figure S41. Plot of magnetization vs. time used to derive relaxation times for **2** at 3.5 K. **S47**
Figure S42. Plot of magnetization vs. time used to derive relaxation times for **2** at 4.0 K. **S48**
Figure S43. Plot of magnetization vs. time used to derive relaxation times for **2** at 5.0 K. **S49**
Figure S44. Plot of magnetization vs. time used to derive relaxation times for **2** at 6.0 K. **S50**
Figure S45. Plot of magnetization vs. time used to derive relaxation times for **2** at 8.0 K. **S51**
Figure S46. Plot of magnetization vs. time used to derive relaxation times for **2** at 10.0 K. **S52**
Figure S47. Plot of magnetization vs. time used to derive relaxation times for **2** at 12.0 K. **S53**
Figure S48. Plot of magnetization vs. time used to derive relaxation times for **2** at 14.0 K. **S54**
Figure S49. Plot of magnetization vs. time used to derive relaxation times for **2** at 16.0 K. **S55**
Figure S50. Plot of magnetization (normalized) vs. time used to derive relaxation times for **2** at 14 and 16 K. **S56**
Figure S51. Plot of magnetization (normalized) vs. time used to derive relaxation times for **2** at different temperatures from 1.8 to 16 K. **S57**
Table S4. Pre-exponential factor, *a*, relaxation times, τ (s), and stretch factors, *b*, at various temperatures, *T* (K), for **2**. **S57**
Figure S52. Individual contributions of the multiple magnetic relaxation pathways to the Arrhenius plot of **2** at 0 Oe and plot of natural log of the relaxation time versus the inverse temperature (temperature range 1.8 to 66 K) for **2**. **S58**
Figure S53. Plot of natural log of the relaxation time versus the inverse temperature for **1** (temperature range 2 to 64 K) and for **2** (temperature range 1.8 to 66 K). **S59**
Table S5. Summary of relaxations times τ of $[(NHAr^*)_2Dy][BArF_{24}]$ (**2**) with upper- and lower τ bounds were calculated according to a 1σ model. **S59**

Figure S54. Individual contributions of the multiple magnetic relaxation pathways to the Arrhenius plot of [(NHAr*)$_2$Dy][BArF$_{24}$] (**2**) at 0 dc Oe with errors to a 1σ limit. **S62**
Table S6. Summary of fitted Arrhenius plots of (NHAr*)$_2$DyCl (**1**) and [(NHAr*)$_2$Dy][BArF$_{24}$] (**2**) to a 1σ model. **S62**
Figure S55. Plot of magnetization (*M*) vs dc magnetic field (*H*) at an average sweep rate of 100 Oe/s for **1** at 1.8 K. **S63**
Figure S56. Plot of magnetization (*M*) vs dc magnetic field (*H*) at an average sweep rate of 100 Oe/s for **1** at 2.0 K. **S63**
Figure S57. Plot of magnetization (*M*) vs dc magnetic field (*H*) at an average sweep rate of 100 Oe/s for **1** at 3.0 K. **S64**
Figure S58. Plot of magnetization (*M*) vs dc magnetic field (*H*) at an average sweep rate of 100 Oe/s for **1** at 4.0 K. **S64**
Figure S59. Plot of magnetization (*M*) vs dc magnetic field (*H*) at an average sweep rate of 100 Oe/s for **1** at 5.0 K. **S65**
Figure S60. Plot of magnetization (*M*) vs dc magnetic field (*H*) at an average sweep rate of 100 Oe/s for **1** at 6.0 K. **S65**
Figure S61. Plot of magnetization (*M*) vs dc magnetic field (*H*) at an average sweep rate of 100 Oe/s for **1** at 7.0 K. **S66**
Figure S62. Plot of magnetization (*M*) vs dc magnetic field (*H*) at an average sweep rate of 100 Oe/s for **1** at 8.0 K. **S66**
Figure S63. Plot of magnetization (*M*) vs dc magnetic field (*H*) at an average sweep rate of 100 Oe/s for **2** at 1.8 K. **S67**
Figure S64. Plot of magnetization (*M*) vs dc magnetic field (*H*) at an average sweep rate of 100 Oe/s for **2** at 2.0 K. **S67**
Figure S65. Plot of magnetization (*M*) vs dc magnetic field (*H*) at an average sweep rate of 100 Oe/s for **2** at 4.0 K. **S68**
Figure S66. Plot of magnetization (*M*) vs dc magnetic field (*H*) at an average sweep rate of 100 Oe/s for **2** at 6.0 K. **S68**
Figure S67. Plot of magnetization (*M*) vs dc magnetic field (*H*) at an average sweep rate of 100 Oe/s for **2** at 8.0 K. **S69**
Figure S68. Plot of magnetization (*M*) vs dc magnetic field (*H*) at an average sweep rate of 100 Oe/s for **2** at 10.0 K. **S69**
Figure S69. Plot of magnetization (*M*) vs dc magnetic field (*H*) at an average sweep rate of 100 Oe/s for **2** at 12.0 K. **S70**
Figure S70. Plot of magnetization (*M*) vs dc magnetic field (*H*) at an average sweep rate of 100 Oe/s for **2** at 14.0 K. **S70**
Figure S71. Plot of magnetization (*M*) vs dc magnetic field (*H*) at an average sweep rate of 100 Oe/s for **2** at 16.0 K. **S71**
Figure S72. Magnification of variable-field magnetization data for **2** at an average sweep rate of 100 Oe/s at 16.0 K. **S71**
Figure S73. Plot of magnetization (*M*) vs dc magnetic field (*H*) at an average sweep rate of 100 Oe/s for **2** at 18.0 K. **S72**
Figure S74. Plot of magnetization (*M*) vs dc magnetic field (*H*) at an average sweep rate of 100 Oe/s for **2** at 19.0 K. **S72**
Figure S75. Variable-temperature *M*(*H*) curves for **1** collected from 0 to 7 T. **S73**
Figure S76. Reduced magnetization data for **1**, collected from 2 to 10 K, between 0 and 7 T. **S73**
Figure S77. Variable-temperature *M*(*H*) curves for **2** collected from 0 to 7 T. **S74**

Figure S78. Reduced magnetization data for **2**, collected from 2 to 10 K, between 0 and 7 T. S74

8 *Ab initio* Calculations S75

Table S7. Calculated Kramers doublet (KD) energies, associated magnetic moments, *g*-tensors, and wave function composition for $(NHAr^*)_2DyCl$ (**1**). S76
Table S8. Calculated Kramers doublet (KD) energies, associated magnetic moments, *g*-tensors, and wave function composition for the $[(NHAr^*)_2Dy]^+$ cation in **2**. S76
Table S9. Crystal field parameters calculated for $(NHAr^*)_2DyCl$ (**1**) via the SINGLE_ANISO program. S77
Table S10. Crystal field parameters calculated for $[(NHAr^*)_2Dy]^+$ cation in **2** via the SINGLE_ANISO program. S77
Table S11. Calculated average transition dipole moments for the eight lowest lying Kramers doublets of $(NHAr^*)_2DyCl$ (**1**). S78
Table S12. Calculated average transition dipole moments for the eight lowest lying Kramers doublets of the $[(NHAr^*)_2Dy]^+$ cation in **2**. S79
Figure S79. Calculated relaxation barrier for $(NHAr^*)_2DyCl$ (**1**) with calculated transition magnetic dipole moments. S80
Figure S80. Calculated relaxation barrier for the $[(NHAr^*)_2Dy]^+$ cation in **2** with calculated transition magnetic dipole moments. S81
Figure S81. Variable-temperature dc magnetic susceptibility data of $(NHAr^*)_2DyCl$ (**1**), collected under a 0.1 T applied dc field with *ab initio*-calculated susceptibility. S82
Figure S82. Variable-temperature dc magnetic susceptibility data of $[(NHAr^*)_2Dy][BArF_{24}]$ (**2**), collected under a 0.1 T applied dc field with *ab initio*-calculated susceptibility. S83

9 TDDFT Calculations S83

Table S13. TDDFT calculated transitions for $(NHAr^*)_2DyCl$ (**1**) with MO depictions for primary transitions. S84
Table S14. TDDFT calculated transitions for $[(NHAr^*)_2Dy][BArF_{24}]$ (**2**) with MO depictions for primary transitions. S85

10 Further Information S86

11 References S87

12 Optimized Geometries S90

1 General Considerations

All manipulations were done under a purified nitrogen atmosphere using either a glove box or Schlenk techniques. *n*-Hexane was dried with CaH_2, distilled under nitrogen to remove oxygen and stored over 4 Å molecular sieves for 12 h prior to use. Diethyl ether was purified by passing through alumina columns to remove water after being sparged with dry nitrogen to remove oxygen. The melting point or decomposition temperature was measured on a Meltemp II instrument. A capillary was packed inside a nitrogen glovebox, sealed, and transferred to the instrument for the measurement. 2,6-dichloroiodobenzene was purchased from Oakwood and, after freeze-pump-thawing three times to remove dissolved gases, was transferred into glovebox. $DyCl_3$ and (trimethylsilyl)methyllithium solution in pentane (0.1 M) were purchased from Sigma-Aldrich and used as received. (Trimethylsilyl)methylpotassium and tosyl azide were syanthesized according to the literature procedure.[1,2] H_2NAr^* was also synthesized according to the literature.[3]

Synthesis of (NHAr*)$_2$DyCl (1): A 20 mL scintillation vial charged with a stir bar was loaded with $DyCl_3$ (108.6 mg, 0.4 mmol, 1 equiv.) and diethyl ether (8 mL). A separate 20 mL scintillation vial was loaded with KNHAr* (433.0 mg, 0.8 mmol, 2 equiv.) and diethyl ether (8 mL). Both solutions were cooled in a dry ice/acetone cold well for 20 min, and then the $DyCl_3$ solution was moved to stir plate to stir. When the solution had thawed enough to stir, a cold solution of KNHAr* was added dropwise over 10 min. The solution was left to stir for 16 h at room temperature. The volatiles were removed in vacuo. The resulting yellow solid was extracted with *n*-hexane, and the solvent was removed in vacuo. A concentrated solution in *n*-hexane was kept overnight at –35 °C in the freezer, resulting in the formation of yellow-colored X-ray quality single crystals (277 mg, 58% yield). Complex **1** is mostly NMR silent. ^{1}H NMR (500 MHz, C_6D_6) δ 3.26, 3.04, 2.87, 2.11, 1.29, 1.23, 1.11, 0.88. Anal. Cald for $C_{72}H_{100}N_2DyCl$: C, 72.58; H, 8.46; N, 2.35. Found: C, 72.32; H, 8.67; N, 2.26. λ_{max} (nm) 1205, 1154, 987, 866, 771, 633, 516 (at higher concentration), 297 (at lower concentration). Decomposition temperature is 82 °C.

Synthesis of $[(NHAr^*)_2Dy][BArF_{24}]$ (2): A 20 mL scintillation vial was charged with **1** (106.9 mg, 0.09 mmol, 1 equiv.), diethyl ether (3 mL), and a magnetic stir bar. A separate 20 mL scintillation vial was loaded with $TlBArF_{24}$ (95.7 mg, 0.09 mmol, 1 equiv.) and diethyl ether (2 mL). The $TlBArF_{24}$ solution was added dropwise to the solution of **1** over the course of a minute. The solution color rapidly changed from yellow to orange. The solution was stirred for 2 h at room temperature. The volatiles were then removed in vacuo, and the remaining residue was dissolved in diethyl ether (2 mL) and filtered through Celite. Orange X-ray quality single crystals were produced by chilling a concentrated diethyl ether solution of **2** in a –35 °C freezer overnight (40.0 mg, 22% yield). The complex is stable up to 3 d at room temperature and for months at –35 °C. ^{1}H NMR (500 MHz, THF-d_8) δ 7.17 (s), 6.90 (s), 6.80 (s), 6.69 (s), 6.17 (s), 5.10 (br), 4.96 (s), 3.52 (s), 3.47, 3.41, 2.99, 2.87, 2.34, 1.73, 1.33, 1.20, 1.19, 1.15, 1.14, 1.12, 0.92, -1.48, -4.34, -17.21, -17.87, -18.97 (br). ^{13}C NMR (126 MHz, THF-d_8) δ 160.84, 160.45, 160.03, 159.65, 149.29, 148.68, 144.23, 134.80, 133.03, 130.34, 129.77, 127.64, 127.42, 126.29, 126.10, 124.10, 122.06, 119.74, 117.81, 115.79, 111.53, 67.57, 35.67, 32.81, 31.63, 31.41, 25.51, 23.80, 15.92, 14.68. ^{19}F NMR (470 MHz, THF-d_8) δ -66.01. Anal. Cald for $C_{104}H_{112}N_2DyBF_{24}$: C, 61.86; H, 5.59; N, 1.39. Found: C, 61.51; H, 5.75; N, 1.44. λ_{max} (nm) 1218, 1189, 1048, 863, 781 (at higher concentration), 418, 293 (at lower concentration). Decomposition temperature is 94 °C.

NMR Spectroscopy

THF-d_8 was purchased from Cambridge Isotope Laboratories, Inc passed through activated alumina before use. NMR spectra were taken on Varian or Bruker instruments located in the Max T. Rogers Instrumentation facility at Michigan State University. ^{1}H, ^{13}C and ^{19}F NMR spectra were recorded on an Avance III HD 500 MHz spectrometer. NMR chemical shifts are reported in ppm and reference to the solvent peaks for ^{1}H NMR (THF-d_8, δ 1.72, 3.58 ppm), ^{13}C NMR (THF-d_8, δ 25.31, 67.21 ppm), and ^{1}H NMR (C_6D_6, δ 7.16 ppm).

UV-vis Spectroscopy

To observe charge transfer bands, low concentrations of **1** and **2** were monitored. UV-vis spectra were collected using an Ocean Optics DH-mini UV-vis spectrophotometer in an N_2 glovebox. In order to obtain molar extinction coefficients for charge transfer bands, different concentrations of solutions were prepared after serial dilutions. All spectra were baseline corrected for diethyl ether and collected at ambient temperature.

IR Spectroscopy

ATR IR spectra were recorded with an Agilent Cary 630 Fourier-transform infrared spectrometer on crushed crystalline solids under an inert nitrogen atmosphere.

Single-Crystal X-ray Diffraction

Single crystal data was collected on XtaLAB Synergy, Dualflex, Hypix diffractometer using $CuK_α$ or $MoK_α$ radiation. Data collection was done at 100 K under a continuous flow of liquid nitrogen. In Olex2 program, crystal structures were solved with ShelXT solution using intrinsic phasing and refined with the ShelXL refinement package using least squares minimization.[4,5] All hydrogens are refined anisotropically. All crystals were stable at room temperature for mounting.

Complex $(NHAr^*)_2DyCl$ (**1**) shows a "B Alert" due to a residual peak between metal and chloride. This is due to the presence of heavy metal ion and therefore cannot be removed after refinement.

Magnetic Susceptibility Measurements

Magnetic susceptibility data were collected on a Quantum Design MPMS3 Superconducting Quantum Interference Device (SQUID) magnetometer. The magnetic sample of $(NHAr^*)_2DyCl$ (**1**) and $[(NHAr^*)_2Dy][BArF_{24}]$ (**2**), were prepared by saturating and covering dried, crushed crystalline solids (**1**: 25.1 mg; **2**: 17.2 mg) with molten eicosane

((**1**: 50.3 mg; **2**: 53.4 mg) at 60 °C) to prevent crystallite torquing and to provide good thermal contact between the sample and the bath. The samples were sealed in an airtight container and transferred to the magnetometer. All data were corrected for diamagnetic contributions from the eicosane, and core diamagnetism was estimated using Pascal's constants.[6]

In addition to the fits displayed in the main text, uncertainty estimates for the ac magnetic susceptibility obtained relaxation times were included to the 1σ level via:

$$\tau_{\pm} = \tau_{\mu}\, exp\left(\pm \frac{1.82\,\sqrt{\alpha}}{1-\alpha}\right) \tag{1}$$

where τ and α are the fitted values from the generalized Debye model for the relaxation time and the distribution of relaxation times, respectively.[7]

Estimated standard deviations for the relaxation times obtained from dc magnetization decay experiments were considered via a 1σ model according to:

$$\sigma^2_{\langle \ln(\tau)\rangle} = \left(\frac{1}{b^2} - 1\right)\frac{\pi^2}{6} \tag{2}$$

$$\tau_{\pm} = exp\left(\langle \ln(\tau)\rangle \pm \sqrt{\sigma^2_{\langle \ln(\tau)\rangle}}\right) \tag{3}$$

where τ and b are the fitted values from a stretched exponential function, and $\tau_{\pm}$ are the limits of the 1σ uncertainty.[8]

The resulting calculated upper and lower τ bounds are given in Table S3 and Table S5, and the resulting fits are shown in Figure S33 and Figure S54. Results of the fitting procedure are summarized in Table S6.

2 Literature Examples of Dy Complexes

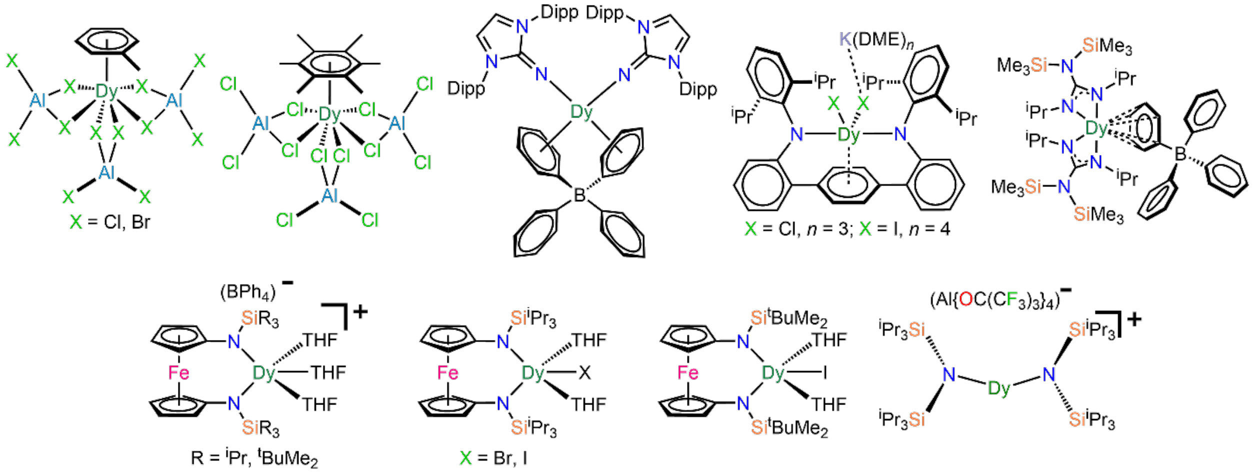

Figure S1. Literature examples of Dy arene and amide complexes used for SMM studies. Top row (from the left): $[(C_7H_8)Dy(AlX_4)_3]$ (X = Cl, Br),[9] $[(C_6Me_6)Dy(AlCl_4)_3]$,[10] $[(L^1)_2Dy(\mu\text{-}\eta^6\text{-}Ph)_2BPh_2]$ (L^1 = 1,3-bis(2,6-diisopropylphenyl)imidazoline-2-imine),[11] $[K(DME)_n][L^2DyCl_2]$ (L^2 = $\{C_6H_4[(2,6\text{-}^iPrC_6H_3)NC_6H_4]_2\}^{2-}$),[12] $[\{(Me_3Si)_2NC(N^iPr)_2\}_2Dy][(\mu\text{-}\eta^6\text{-}Ph)(BPh_3)]$.[13] Bottom row (from the left): $[(NN^R)Dy(THF)_3][BPh_4]$ (NN^R = $(C_5H_4\{NHSiR\})_2Fe$), where R = $^tBuMe_2$ or $^iPr_3$),[14] $(NN^{iPr})DyX(THF)_2$ (X = Br, I),[14] $(NN^{TBS})DyI(THF)_2$ (NN^{TBS} = $(C_5H_4\{NHSi^tBuMe_2\})_2Fe$),[15] $[\{N(Si^iPr_3)_2\}_2Dy][Al\{OC(CF_3)_3\}_4]$.[16]

3 NMR Spectroscopy

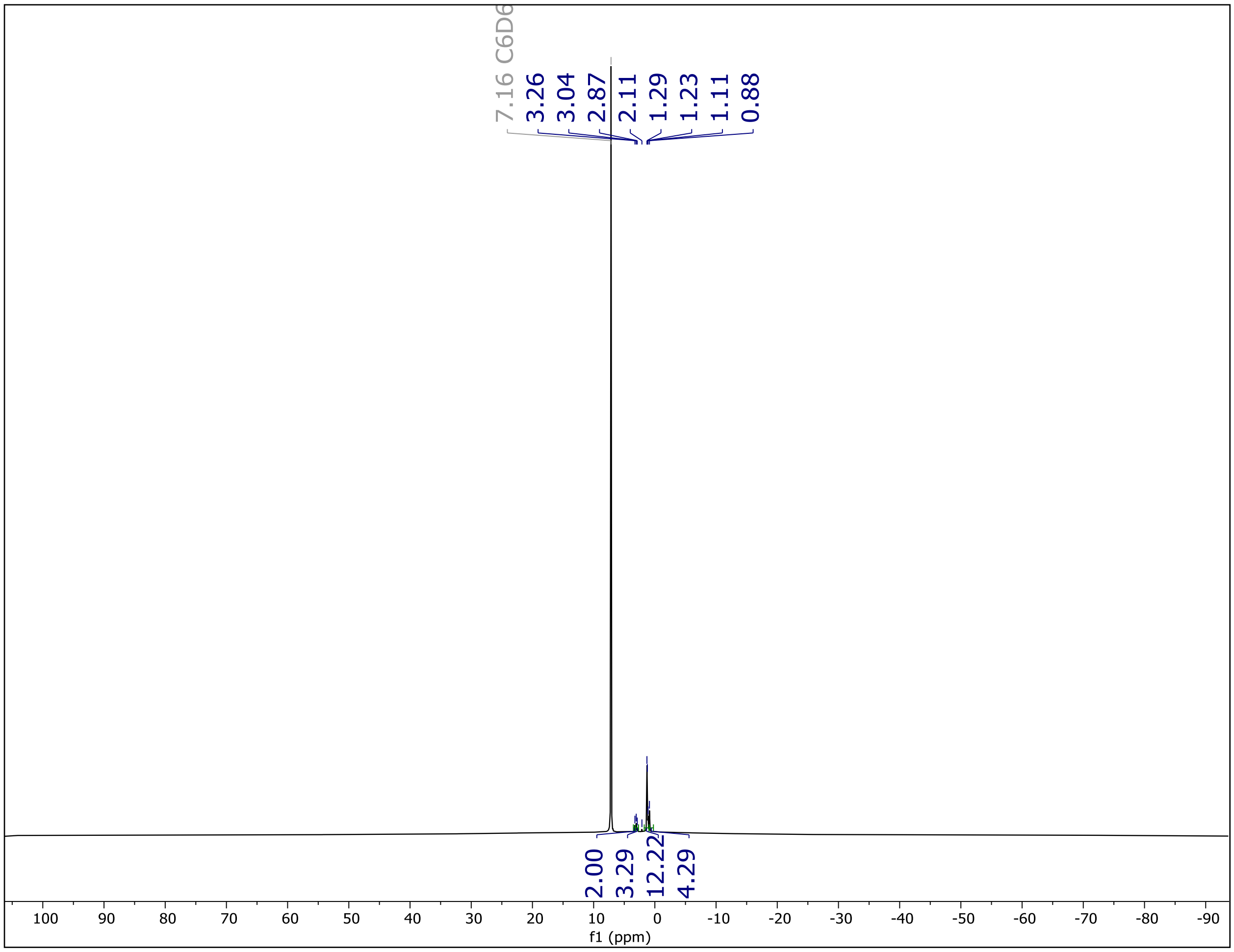

Figure S2. ^1H NMR spectrum of $(NHAr^*)_2DyCl$ (**1**) in C_6D_6 at room temperature.

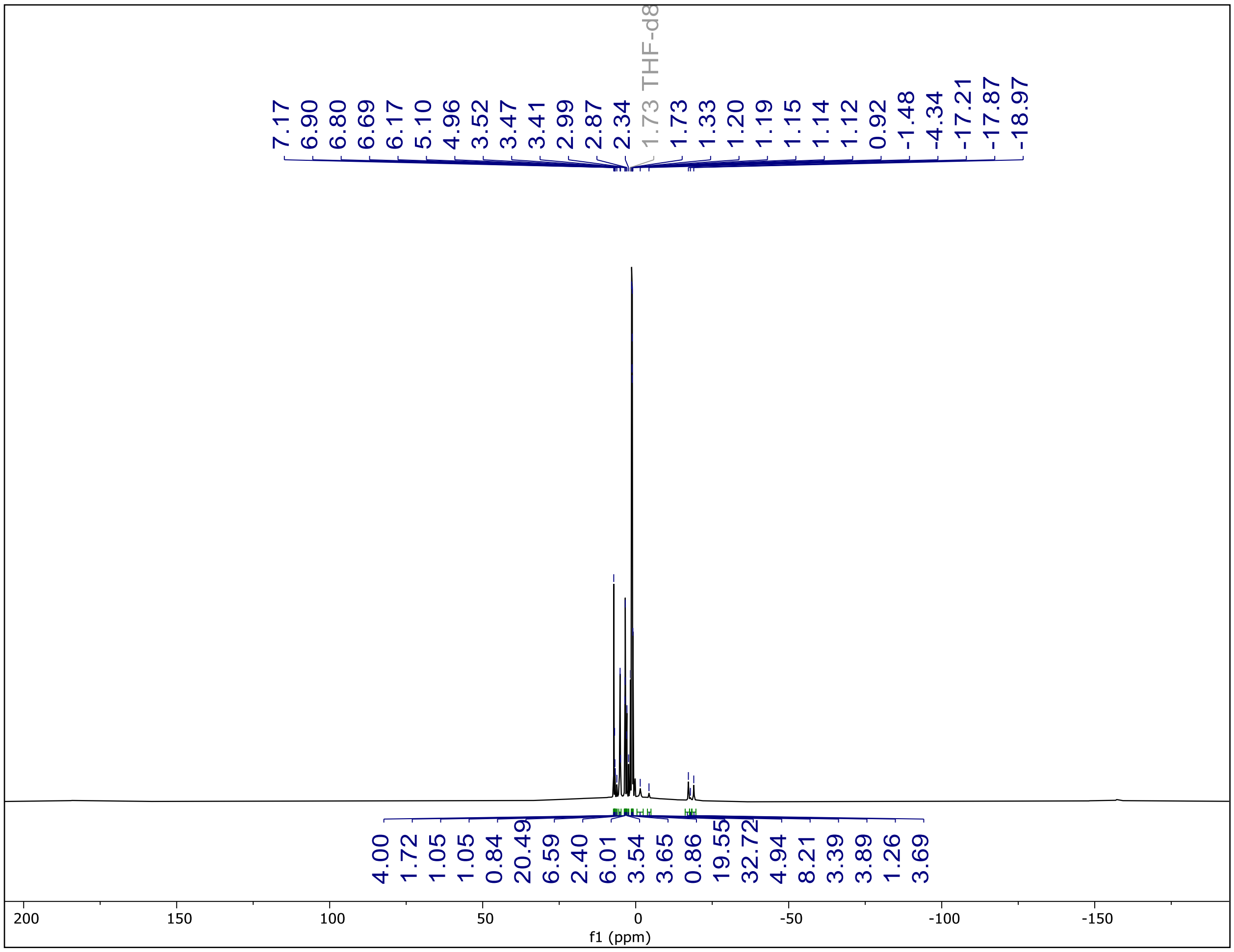

Figure S3. ^{1}H NMR spectrum of [(NHAr*)$_2$Dy][BArF$_{24}$] (**2**) in THF-d_8 at room temperature.

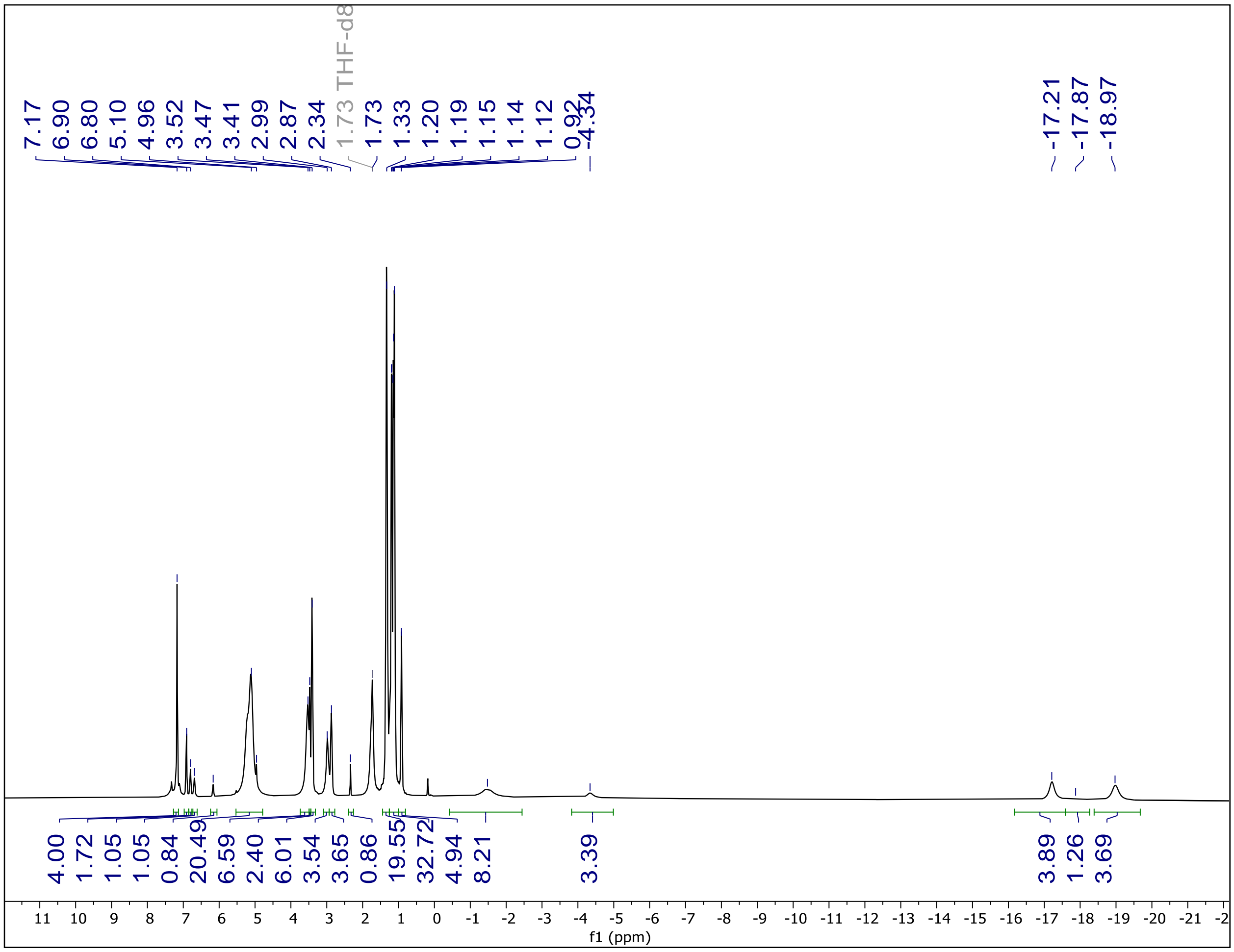

Figure S4. ^{1}H NMR spectrum (zoomed in) of [(NHAr*)$_2$Dy][BArF$_{24}$] (**2**) in THF-d_8 at room temperature.

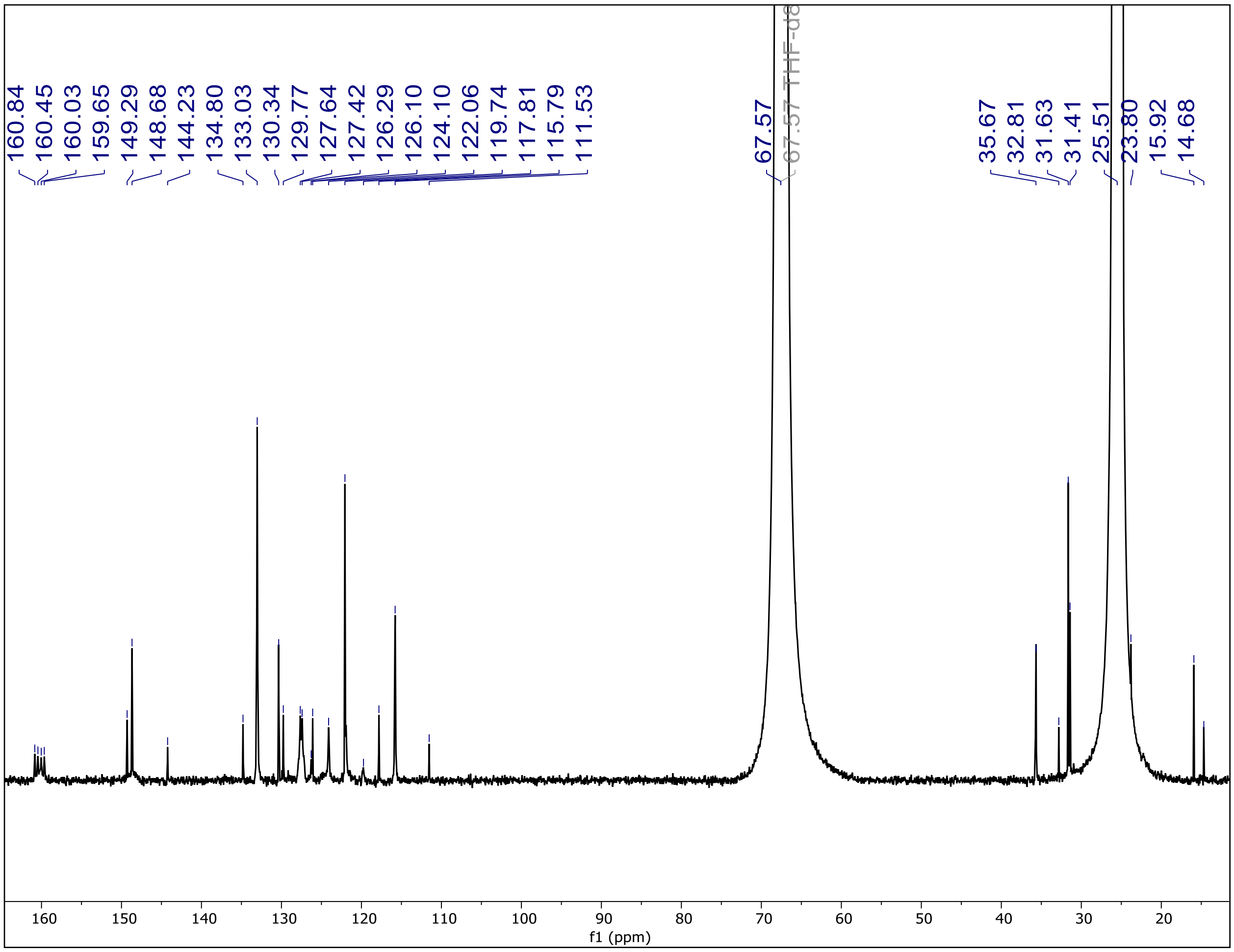

Figure S5. ^{13}C NMR spectrum of [(NHAr*)$_2$Dy][BArF$_{24}$] (**2**) in THF-d_8 at room temperature.

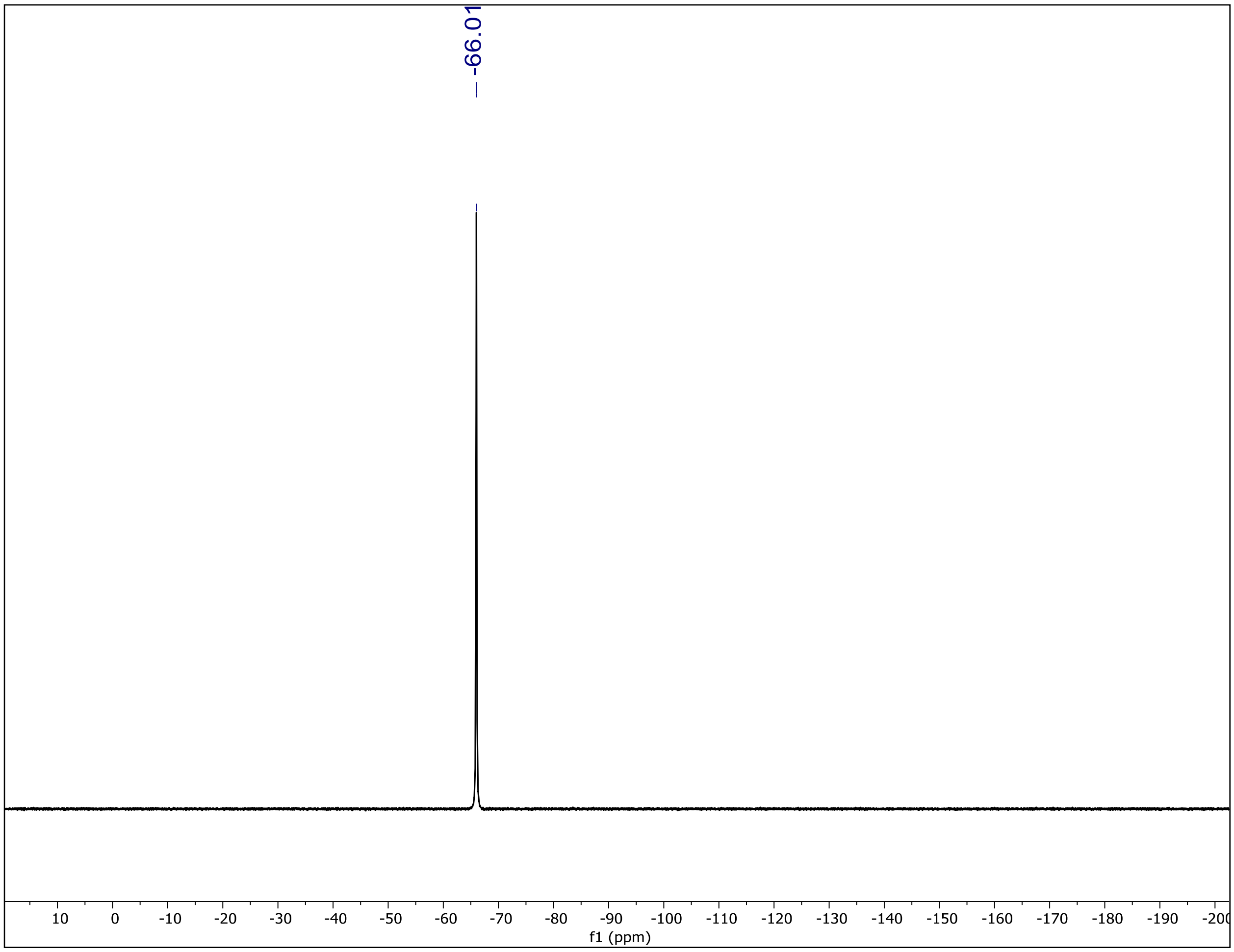

Figure S6. ^{19}F NMR spectrum of $[(NHAr^*)_2Dy][BArF_{24}]$ (**2**) in THF-d_8 at room temperature.

4 UV-vis-NIR Spectroscopy

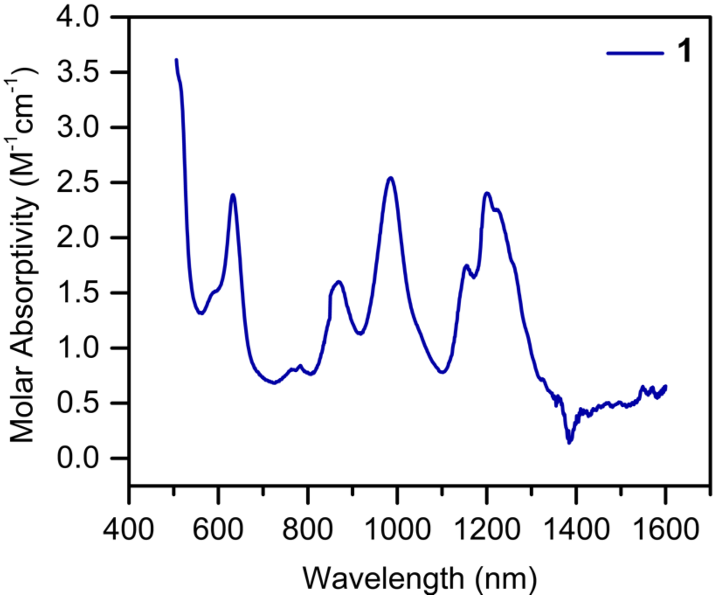

Figure S7. Vis-NIR spectrum of $(NHAr^*)_2DyCl$ (**1**) obtained from 25 μM solution in diethyl ether.

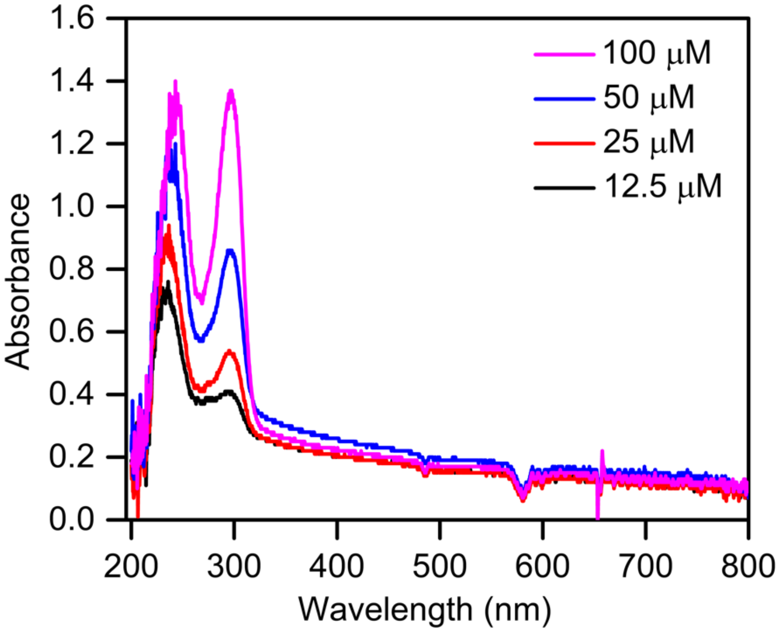

Figure S8. UV-vis spectrum of $(NHAr^*)_2DyCl$ (**1**) obtained from serial dilution of 100 μM solution.

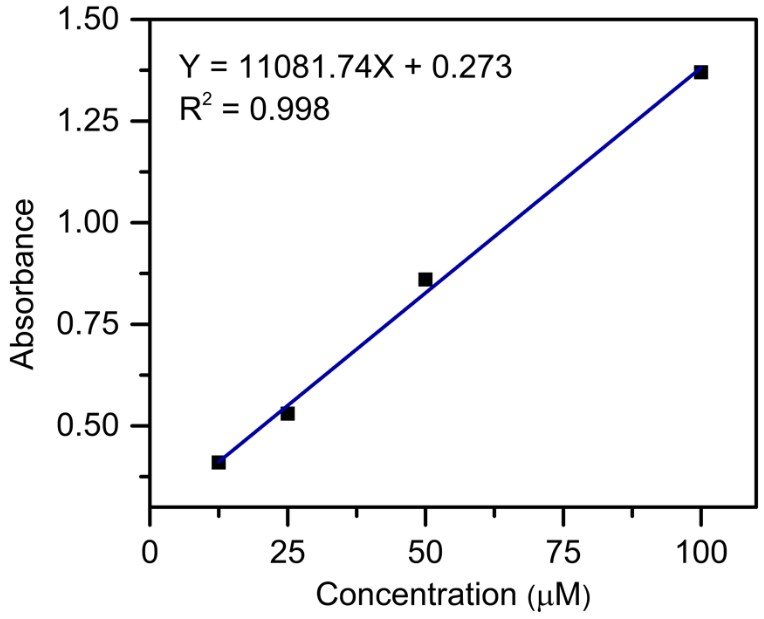

Figure S9. Concentration vs absorbance plot to calculate molar extinction coefficient at 297 nm for $(NHAr^*)_2DyCl$ (**1**) in diethyl ether. The slope of the line provides ε = 1108.1 M^{-1} cm^{-1} for the electronic transition occurring at 297 nm.

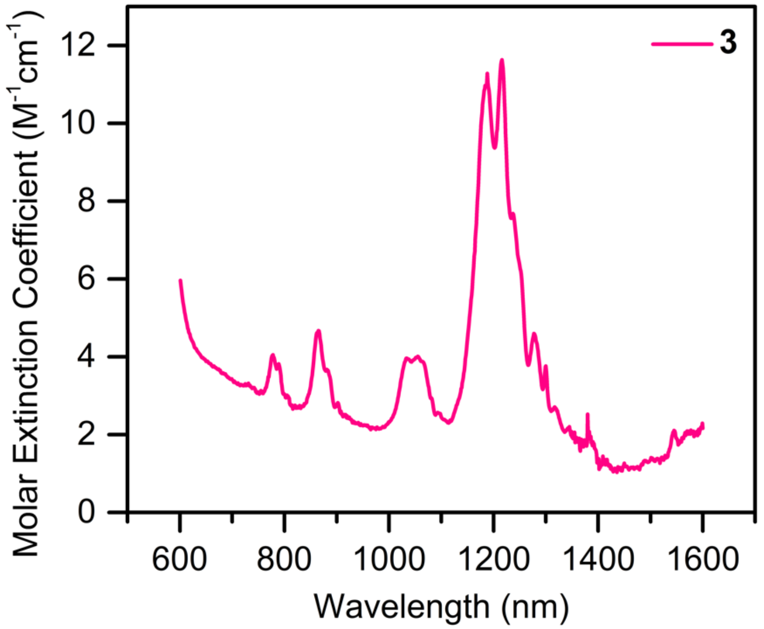

Figure S10. Vis-NIR spectrum of $[(NHAr^*)_2Dy][BArF_{24}]$ (**2**) obtained from 25 μM solution in diethyl ether.

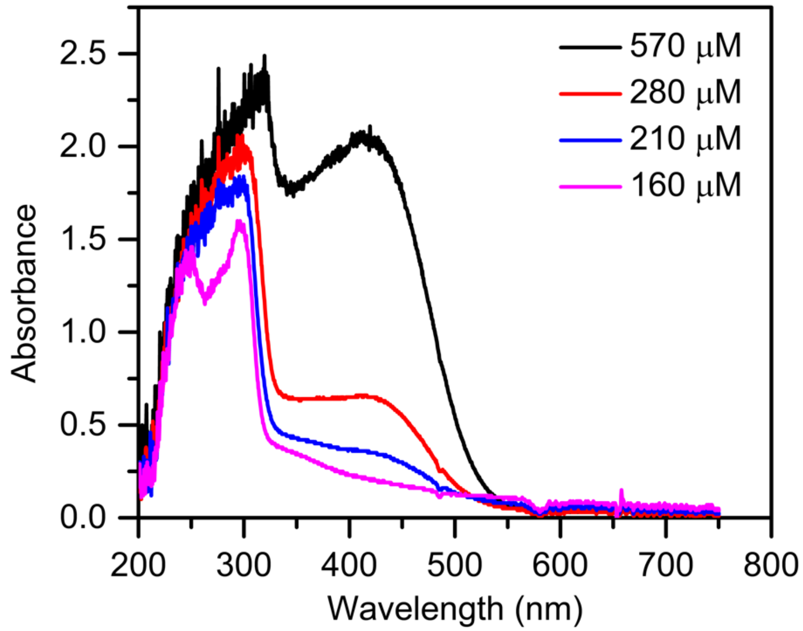

Figure S11. UV-vis spectrum of [(NHAr*)$_2$Dy][BArF$_{24}$] (**2**) obtained from serial dilution of 570 μM solution.

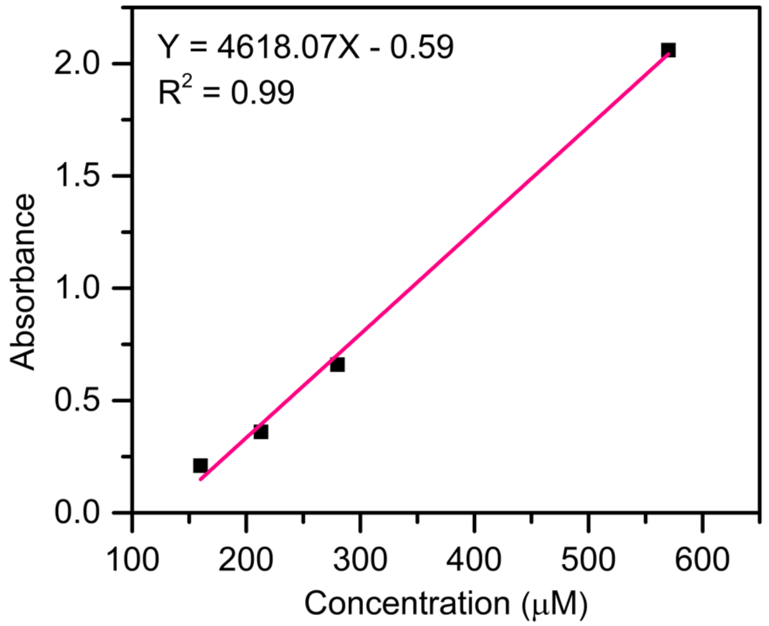

Figure S12. Concentration vs absorbance plot to calculate molar extinction coefficient at 415 nm for [(NHAr*)$_2$Dy][BArF$_{24}$] (**2**) in diethyl ether. The slope of the line provides ε = 4618 M^{-1} cm^{-1} for the electronic transition occurring at 415 nm.

5 FT-IR Spectroscopy

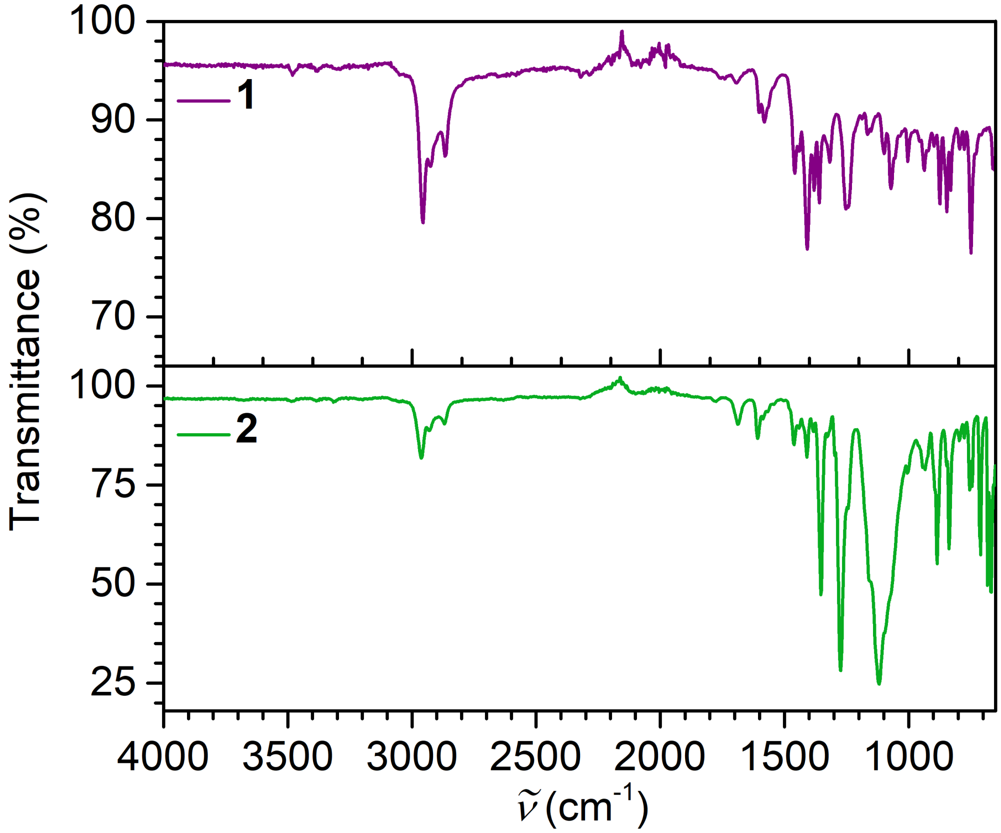

Figure S13. Top: IR spectrum of (NHAr*)$_2$DyCl (**1**, purple) recorded on polycrystalline solids. Bottom: IR spectrum of [(NHAr*)$_2$Dy][BArF$_{24}$] (**2**, green) recorded on polycrystalline solids.

6 Single Crystal X-ray Diffraction

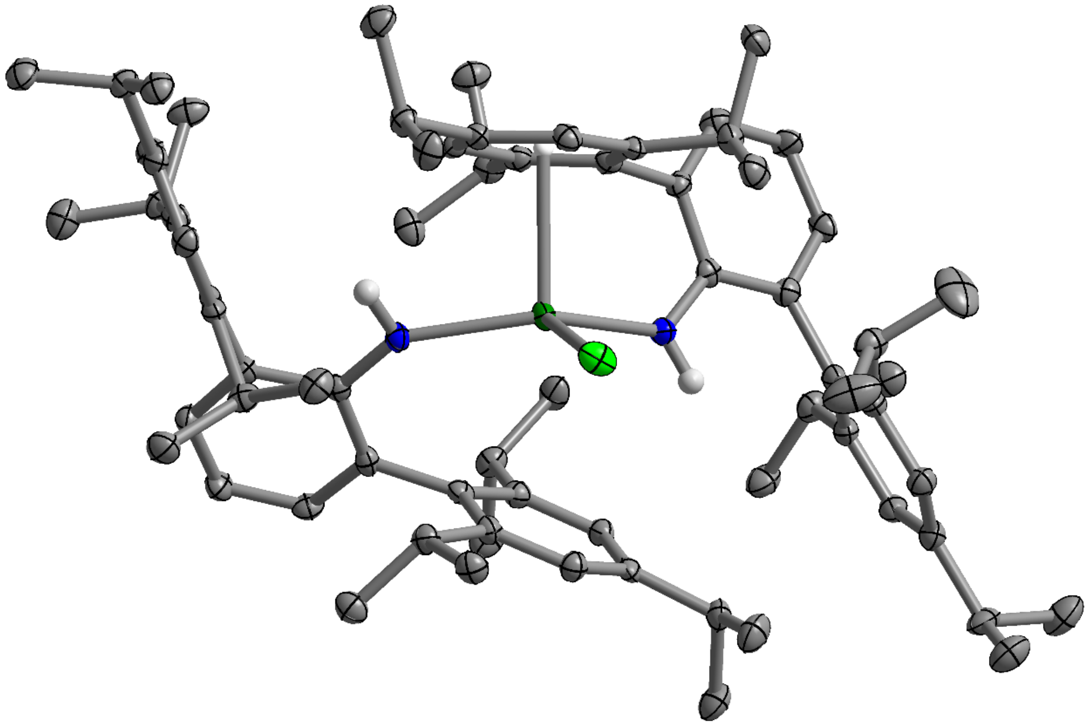

Figure S14. Structure of $(NHAr^*)_2DyCl$ (**1**) with thermal ellipsoids drawn at the 50% probability level. Dark green, blue, green, and gray ellipsoids represent Dy, N, Cl, and C atoms, respectively. Pale gray spheres represent H atoms. H atoms bound to all carbon atoms and a disordered *n*-hexane molecule in the lattice per molecule of **1** have been omitted for clarity.

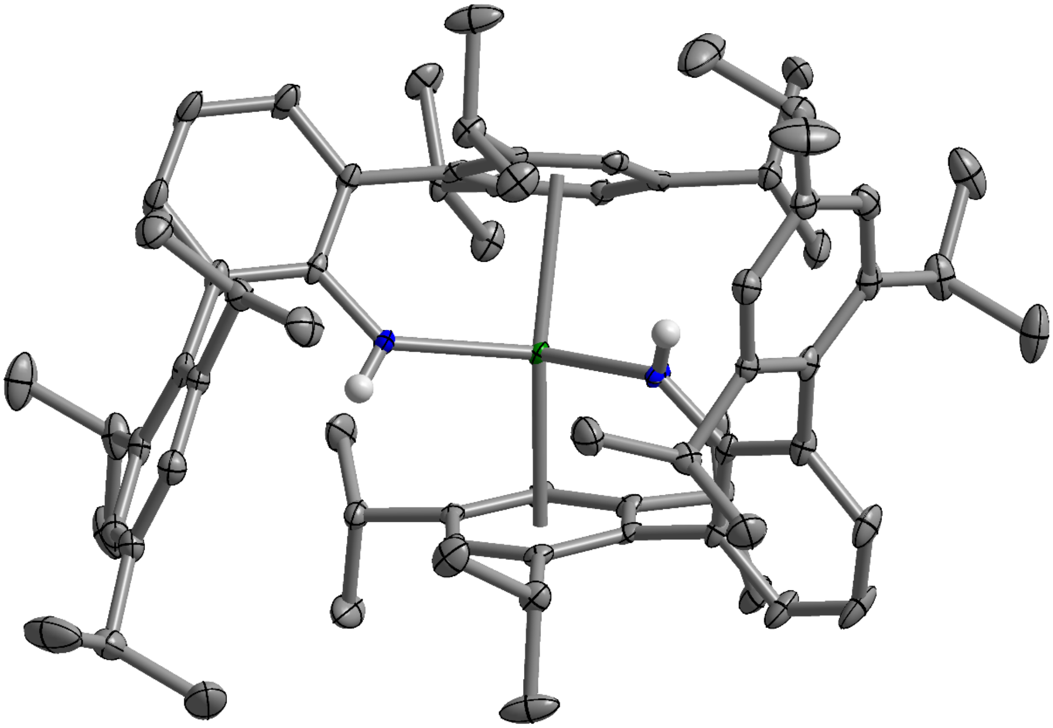

Figure S15. Structure of the cation $[(NHAr^*)_2Dy]^+$ in a crystal of **2**, with thermal ellipsoids drawn at the 50% probability level. Dark green, blue, and gray ellipsoids represent Dy, N, and C atoms, respectively. Pale gray spheres represent H atoms. H atoms bound to all carbon atoms and the $[BArF_{24}]^-$ anion have been omitted for clarity.

Table S1. Metric data from the crystal structures of $(NHAr^*)_2DyCl$ (**1**) and $[(NHAr^*)_2Dy][BArF_{24}]$ (**2**).

Complex	$(NHAr^*)_2DyCl$ (1)	$[(NHAr^*)_2Dy][BArF_{24}]$ (2)
Dy–N	2.258(3), 2.235(3)	2.222(2)
Dy–Cl	2.515(1)	-
N–C_{ipso}	1.372(5), 1.374(5)	1.394(4)
Dy–Cnt	2.542	2.496
Dy–N–C_{ipso}	137.9(7), 131.9(3)	131.5(2)
N–Dy–N	140.9(1)	121.7(1)
N–Dy–Cl	106.1(1), 104.9(1)	-
Cnt–Dy–N	98.2, 94.3	94.3, 98.2
Cnt–Dy–Cnt	-	152.6
Cnt–Dy–Cl	107.9	-
η^6-C_{Ar}–C_{Ar}	1.439(6), 1.411(6), 1.394(6), 1.397(6), 1.398(6), 1.406(6)	1.419(4), 1.414(5), 1.392(4), 1.390(4), 1.406(4), 1.415(4)
Average C_{Ar}–C_{Ar} bond	1.408(6)	1.406(4)

Table S2. Crystallographic data and structural refinement of $(NHAr^*)_2DyCl$ (**1**) and $[(NHAr^*)_2Dy][BArF_{24}]$ (**2**). **1** crystallized with one ^{n}hexane molecule in the lattice as $(NHAr^*)_2DyCl\cdot C_6H_{14}$.

Complex	$(NHAr^*)_2DyCl$ (**1**)	$[(NHAr^*)_2Dy][BArF_{24}]$ (**2**)
Empirical formula	$C_{78}H_{114}ClDyN_2$	$C_{104}H_{112}BDyF_{24}N_2$
CCDC	2361468	2361267
Formula weight	1277.66	2019.26
Temperature / K	99.96(16)	99.99(10)
Crystal system	triclinic	monoclinic
Space group	*P*-1	*C*2
a / Å	9.54318(14)	21.2676(3)
b / Å	16.7779(2)	15.6856(2)
c / Å	22.9544(3)	14.7701(2)
α / °	88.7134(10)	90
β / °	78.3872(12)	92.7530(10)
γ / °	73.9747(13)	90
Volume/Å^3	3458.07(9)	4921.55(11)
Z	2	2
ρ_{calc} / g/cm^3	1.227	1.363
M / mm^{-1}	6.441	0.851
F(000)	1358.0	2074.0
Crystal size/mm^3	0.107 × 0.103 × 0.04	0.135 × 0.098 × 0.086
Radiation	Cu K_α (λ = 1.54184)	Mo K_α (λ = 0.71073)
2Θ range for data collection / °	5.484 to 153.926	4.188 to 61.866
Index ranges	$-11 \le h \le 12$, $-21 \le k \le 19$, $-28 \le l \le 25$	$-29 \le h \le 29$, $-21 \le k \le 21$, $-20 \le l \le 20$
Reflections collected	40403	44781
Independent reflections	13657 [R_{int} = 0.0519, R_σ = 0.0592]	12201 [R_{int} = 0.0299, R_σ = 0.0293]
Data/restraints/ parameters	13657/28/782	12201/1/617

Goodness-of-fit on F^2	1.124	1.033
Final R indexes [I>=2σ (I)]	R_1 = 0.0512, wR_2 = 0.1318	R_1 = 0.0252, wR_2 = 0.0613
Final R indexes [all data]	R_1 = 0.0545, wR_2 = 0.1335	R_1 = 0.0254, wR_2 = 0.0614
Largest diff. peak/hole / e Å^{-3}	2.31/-0.79	0.95/-0.60

7 Magnetic Data

Data Derived from Static Field Dc Magnetic Measurements

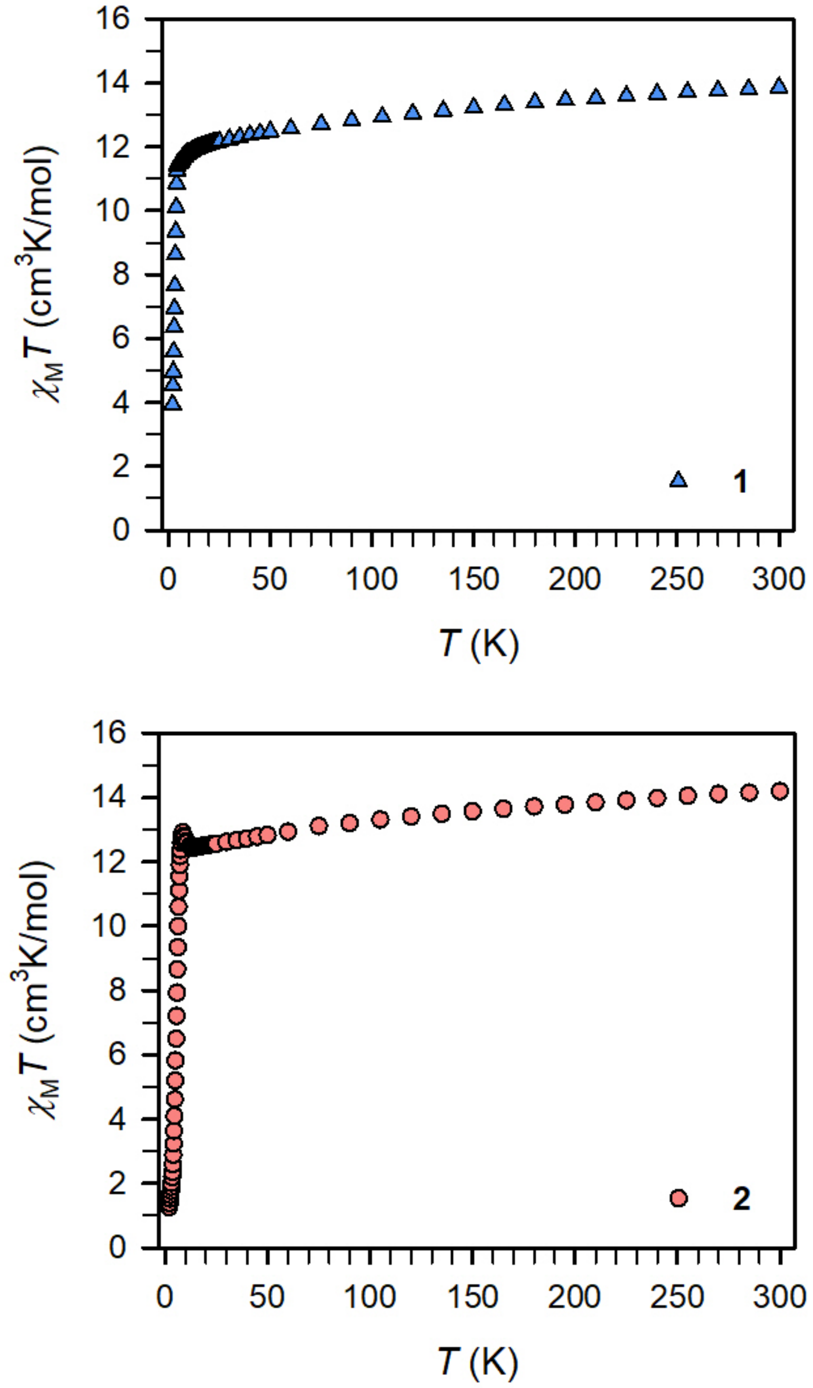

Figure S16. Temperature dependence of the $\chi_M T$ product for polycrystalline samples of **1** (top) and **2** (bottom) under a 0.1 T applied dc field.

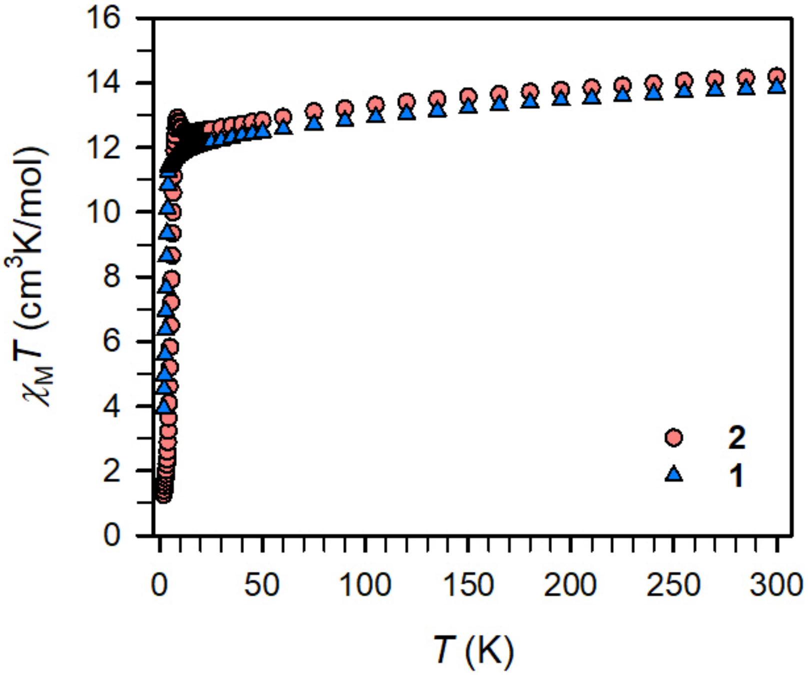

Figure S17. Temperature dependence of the $\chi_{M}T$ product for polycrystalline samples of **1** (blue) and **2** (red) under a 0.1 T applied dc field.

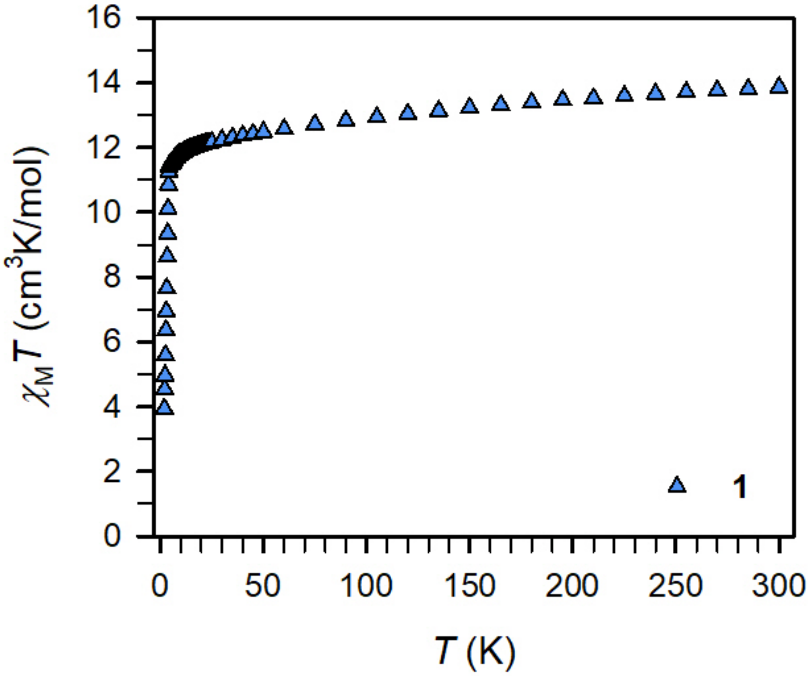

Figure S18. Variable-temperature dc magnetic susceptibility data for a restrained polycrystalline sample of **1** collected under a 0.1 T applied dc field.

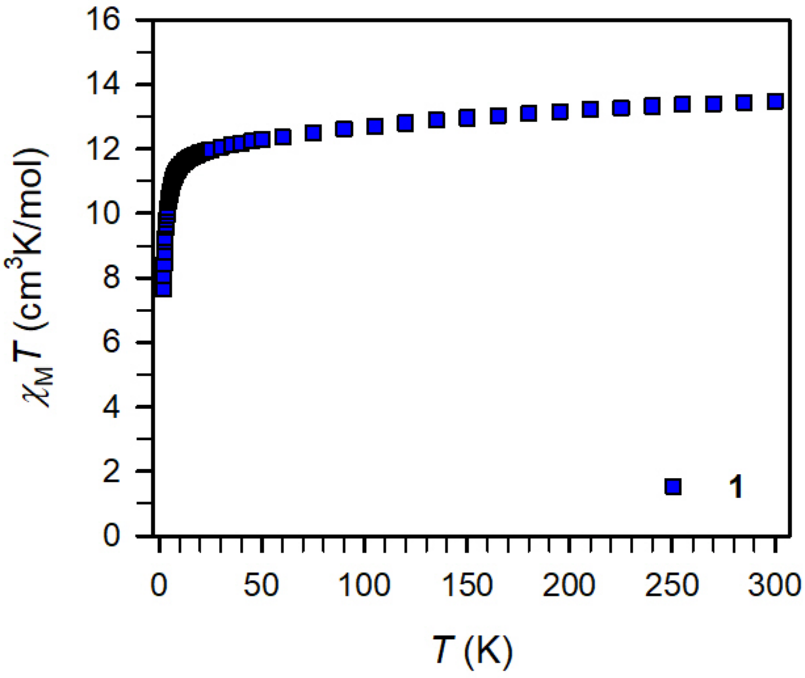

Figure S19. Variable-temperature dc magnetic susceptibility data for a restrained polycrystalline sample of **1** collected under a 0.5 T applied dc field.

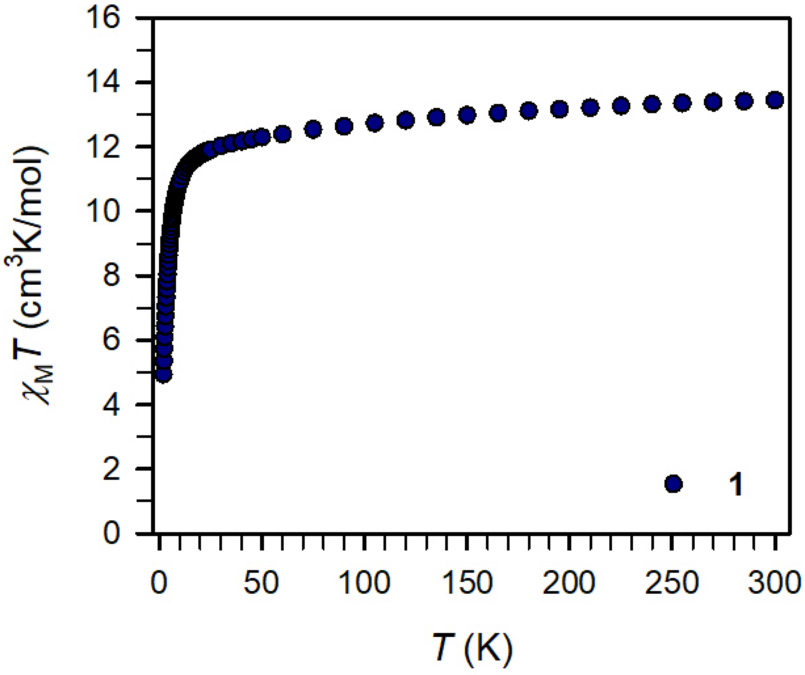

Figure S20. Variable-temperature dc magnetic susceptibility data for a restrained polycrystalline sample of **1** collected under a 1 T applied dc field.

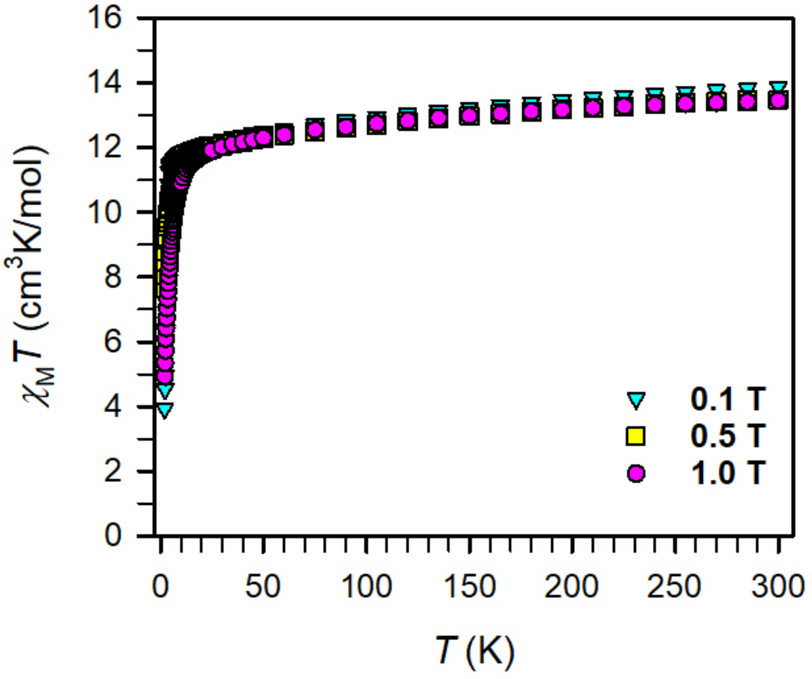

Figure S21. Variable-temperature dc magnetic susceptibility data for a restrained polycrystalline sample of **1** collected under 0.1 T (turquoise triangles), 0.5 T (yellow squares), and 1.0 T (pink circles) applied dc fields.

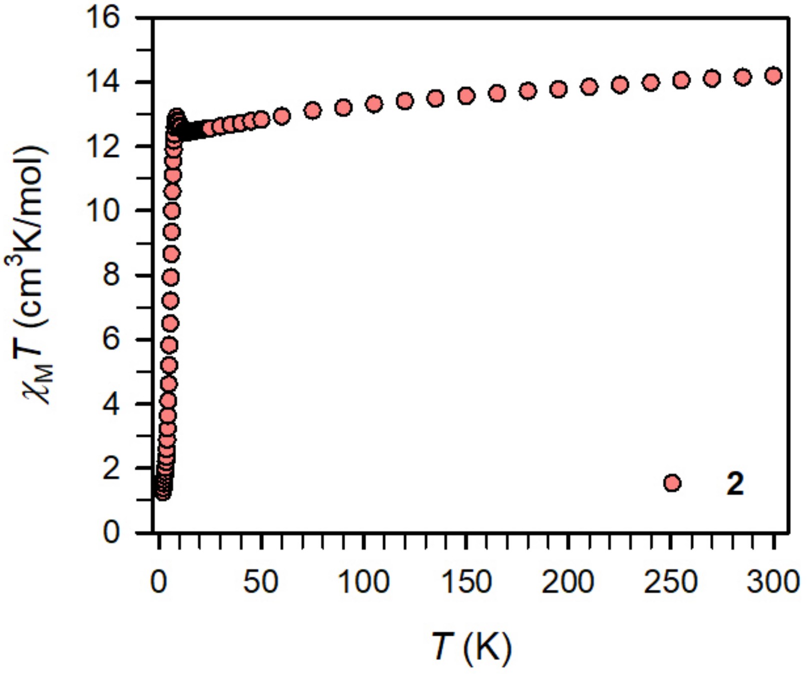

Figure S22. Variable-temperature dc magnetic susceptibility data for a restrained polycrystalline sample of **2** collected under a 0.1 T applied dc field.

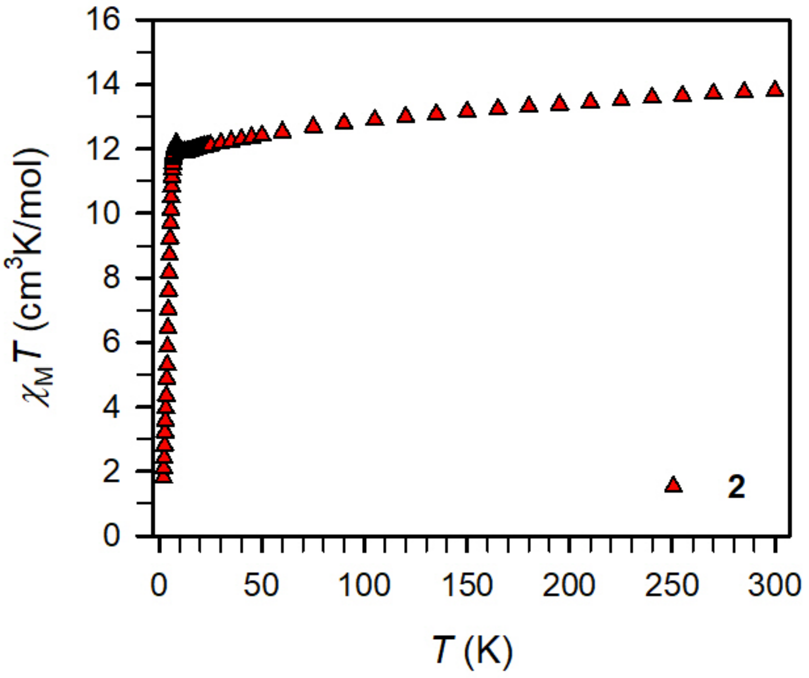

Figure S23. Variable-temperature dc magnetic susceptibility data for a restrained polycrystalline sample of **2** collected under a 0.5 T applied dc field.

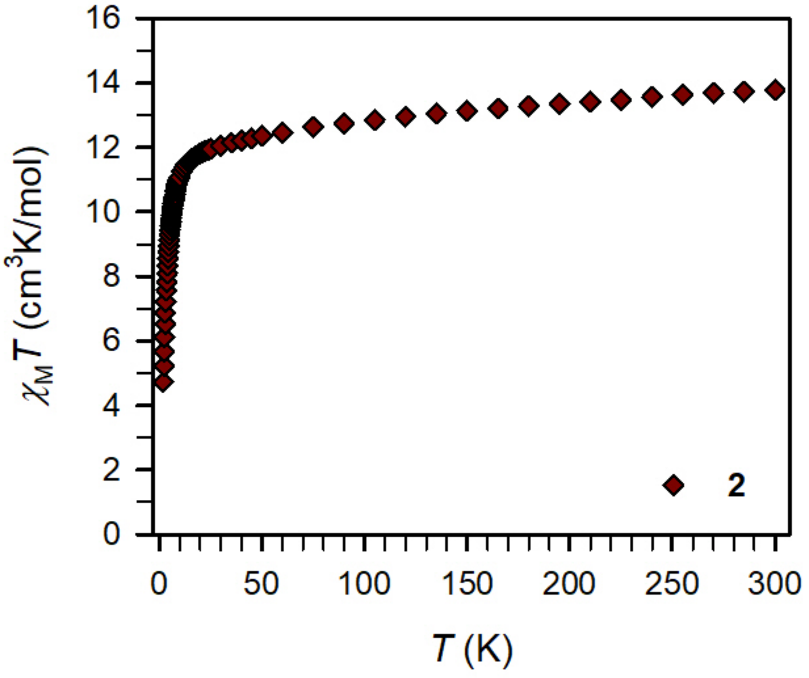

Figure S24. Variable-temperature dc magnetic susceptibility data for a restrained polycrystalline sample of **2** collected under a 1.0 T applied dc field.

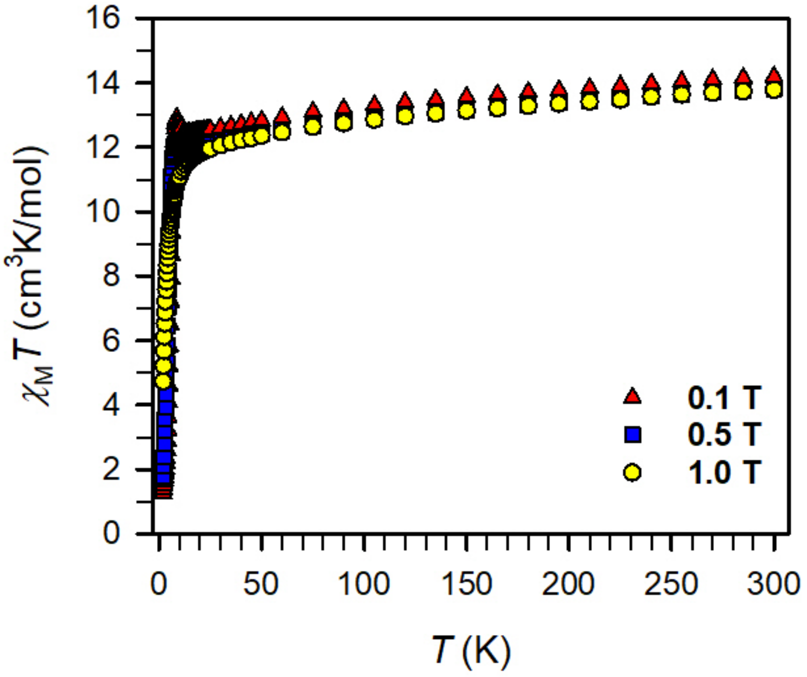

Figure S25. Variable-temperature dc magnetic susceptibility data for a restrained polycrystalline sample of **2** collected under 0.1 T (red triangles), 0.5 T (blue squares), and 1.0 T (yellow circles) applied dc fields.

Data derived from Ac Magnetic Susceptibility Measurements

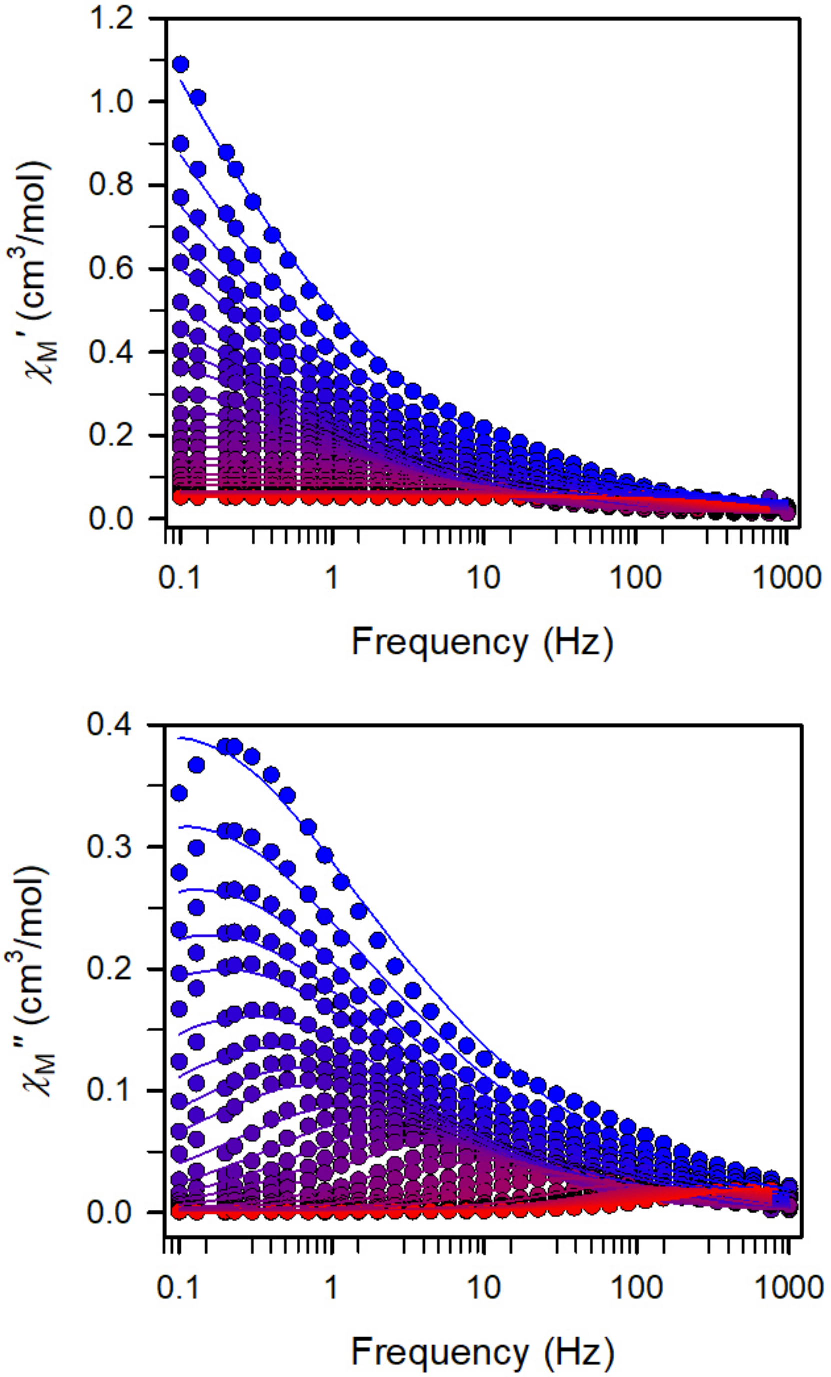

Figure S26. Variable-temperature, variable-frequency in-phase (χ_M') (top) and out-of-phase (χ_M'') (bottom) ac magnetic susceptibility data collected for **1** under a zero applied dc field from 2.0 to 64.0 K. Solid lines represent fits to the data, as described in the main text. A non-zero χ_M'' out-of-phase signal indicates the presence of an energy barrier to spin reversal.

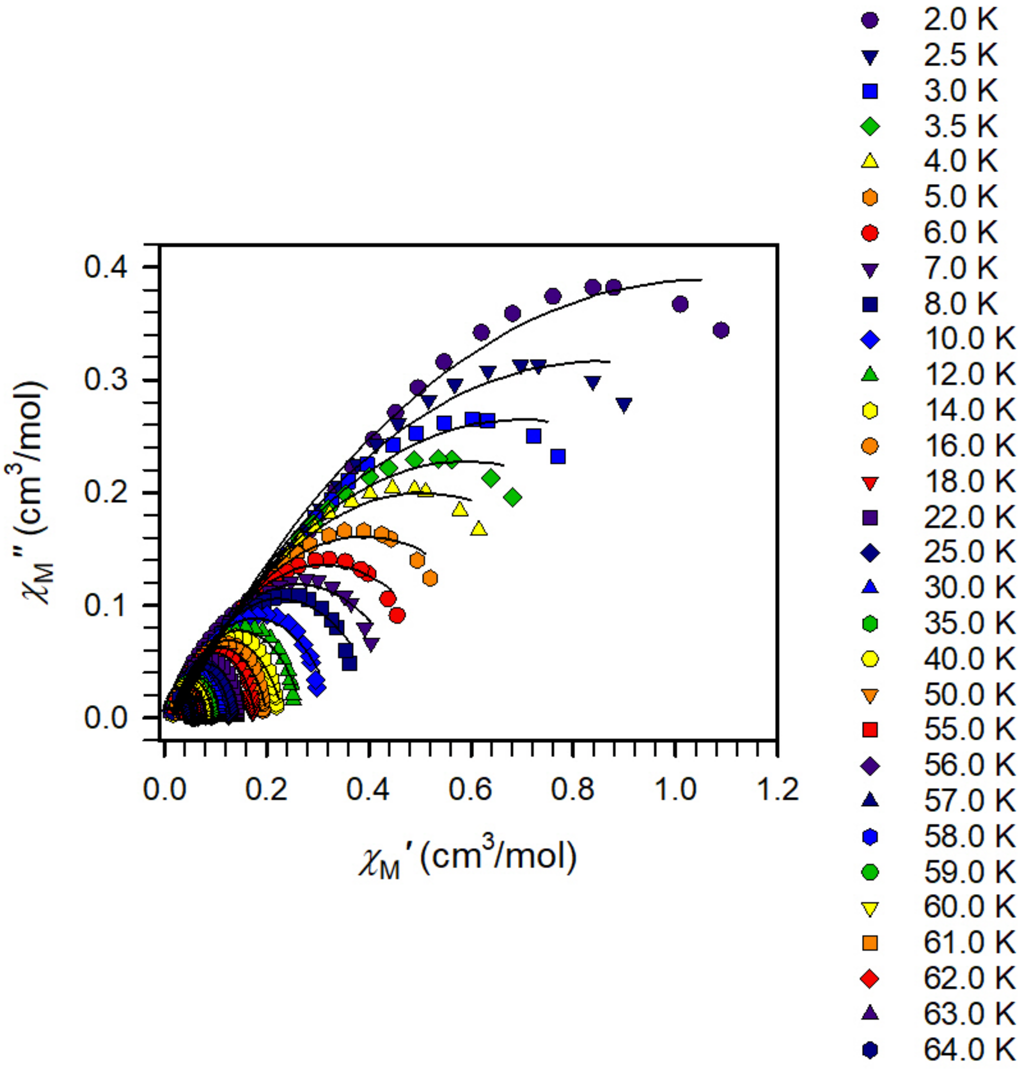

Figure S27. Cole-Cole (Argand) plots for ac susceptibility collected from 2.0 to 64.0 K under a zero applied dc field for **1**. Symbols represent the experimental data points and the points representing the fits are connected by black solid lines.

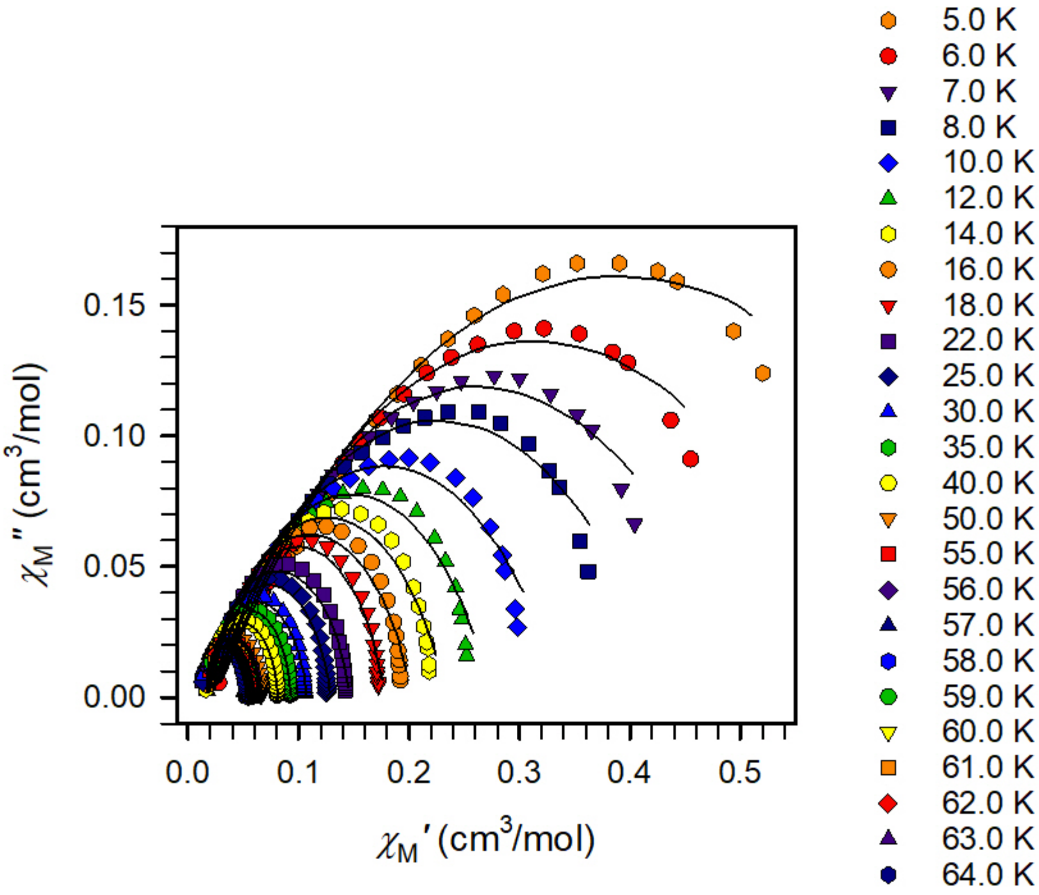

Figure S28. Cole-Cole (Argand) plots for ac susceptibility collected from 5.0 to 64.0 K under a zero applied dc field for **1**. Symbols represent the experimental data points and the points representing the fits are connected by black solid lines.

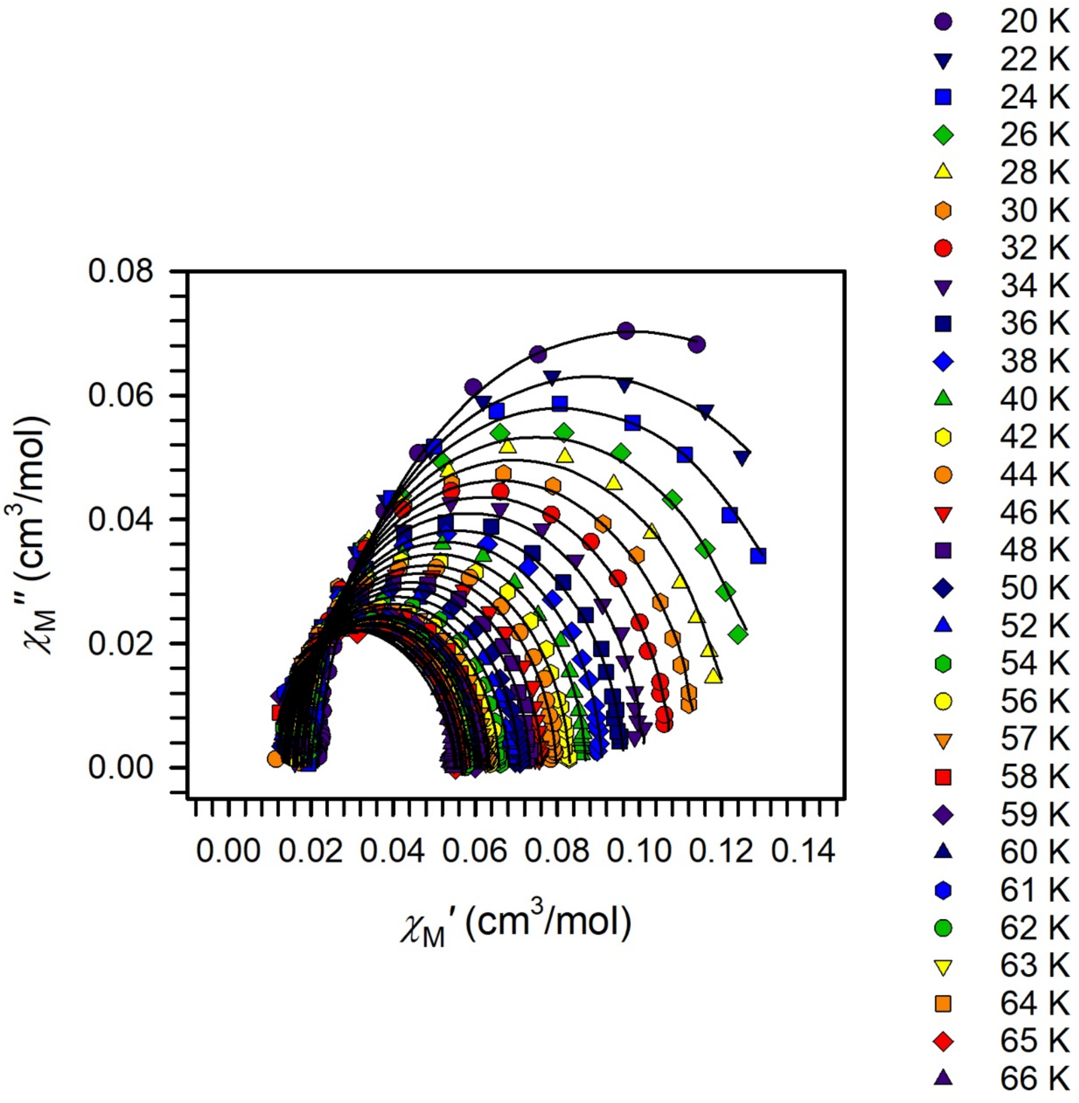

Figure S29. Cole-Cole (Argand) plots for ac susceptibility collected from 20.0 to 66.0 K under a zero applied dc field for **2**. Symbols represent the experimental data points and the points representing the fits are connected by black solid lines.

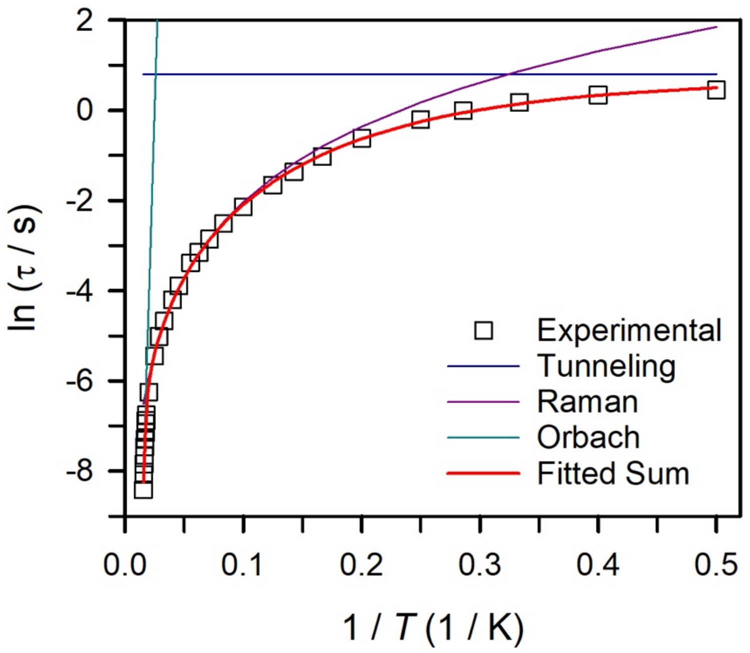

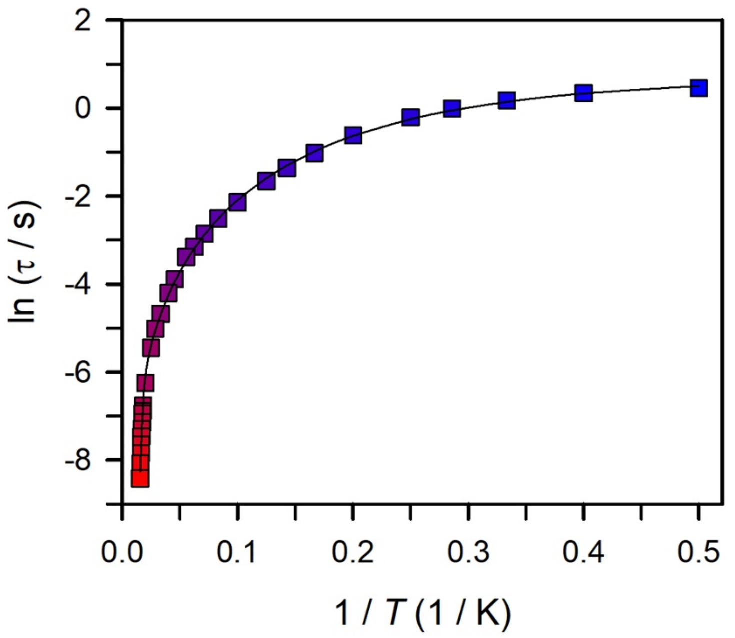

Figure S30. (top) Individual contributions of the multiple magnetic relaxation pathways to the Arrhenius plot of **1** at 0 Oe. Individual parameters used to calculate the contributions are given in main text Table 1. (bottom) Plot of natural log of the relaxation time versus the inverse temperature (temperature range 2 to 64 K) for **1**. Blue to red squares represent data extracted from ac magnetic susceptibility measurements. The black line represents a fit to Orbach, Raman, and quantum tunneling relaxation processes.

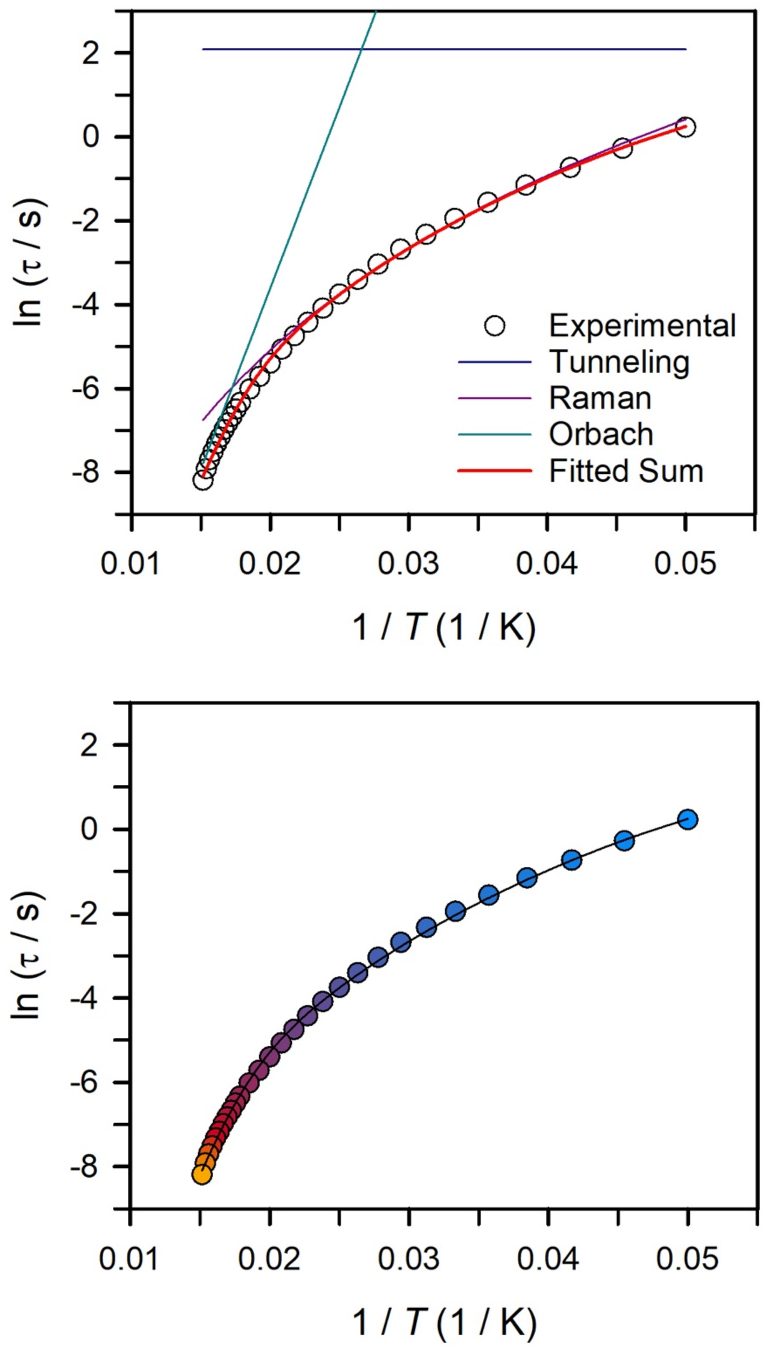

Figure S31. (top) Individual contributions of the multiple magnetic relaxation pathways to the Arrhenius plot of **2** at 0 Oe. Individual parameters used to calculate the contributions are given in Table 1 (main text). (bottom) Plot of natural log of the relaxation time versus the inverse temperature (temperature range 20 to 66 K) for **2**. Pale blue to red circles represent data extracted from ac magnetic susceptibility measurements. The black line represents a fit to Orbach relaxation process, a Raman relaxation process, and a quantum tunneling pathway.

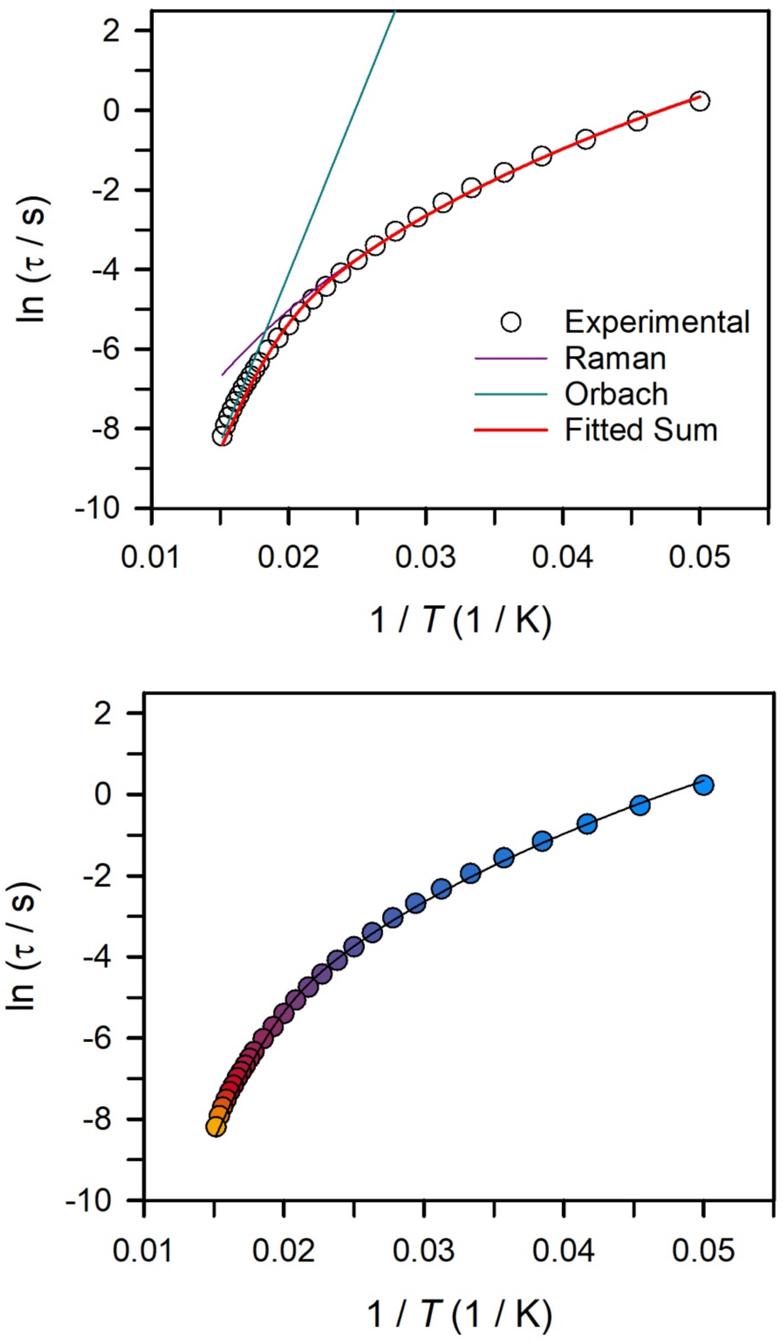

Figure S32. (top) Individual contributions of the multiple magnetic relaxation pathways to the Arrhenius plot of **2** at 0 Oe. Individual parameters used to calculate the contributions are given in Table 1 (main text). (bottom) Plot of natural log of the relaxation time versus the inverse temperature (temperature range 20 to 66 K) for **2**. Pale blue to red circles represent data extracted from ac magnetic susceptibility measurements. The black line represents a fit to Orbach and Raman relaxation processes.

Table S3. Summary of relaxation times of $(NHAr^*)_2DyCl$ (**1**), obtained from fitting the Cole-Cole plots for ac magnetic susceptibility to a generalized Debye model. Upper- and lower τ bounds were calculated according to a 1σ model.

**T (K)**	**T^{-1} (K^{-1})**	**τ (s)**	**ln(τ) (ln(s))**	**α**	**σ ln(τ) (ln(s))**	**τ_{upper} (s)**	**τ_{lower} (s)**
2	0.5000	1.570E+00	0.451	0.540	2.903	2.862E+01	8.610E-02
2.5	0.4000	1.410E+00	0.344	0.533	2.843	2.420E+01	8.217E-02
3	0.3333	1.192E+00	0.175	0.522	2.755	1.873E+01	7.584E-02
3.5	0.2857	9.897E-01	-0.010	0.511	2.663	1.419E+01	6.902E-02
4	0.2500	8.131E-01	-0.207	0.499	2.569	1.062E+01	6.226E-02
5	0.2000	5.378E-01	-0.620	0.471	2.359	5.691E+00	5.083E-02
6	0.1667	3.602E-01	-1.021	0.440	2.159	3.119E+00	4.159E-02
7	0.1429	2.567E-01	-1.360	0.417	2.017	1.929E+00	3.416E-02
8	0.1250	1.907E-01	-1.657	0.394	1.884	1.255E+00	2.897E-02
10	0.1000	1.176E-01	-2.140	0.351	1.660	6.188E-01	2.236E-02
12	0.0833	8.135E-02	-2.509	0.295	1.404	3.311E-01	1.999E-02
14	0.0714	5.755E-02	-2.855	0.274	1.314	2.142E-01	1.546E-02
16	0.0625	4.284E-02	-3.150	0.244	1.190	1.409E-01	1.303E-02
18	0.0556	3.403E-02	-3.381	0.194	0.996	9.215E-02	1.256E-02
22	0.0455	2.046E-02	-3.889	0.187	0.969	5.393E-02	7.759E-03
25	0.0400	1.499E-02	-4.201	0.170	0.902	3.695E-02	6.080E-03
30	0.0333	9.367E-03	-4.671	0.145	0.812	2.110E-02	4.158E-03
35	0.0286	6.655E-03	-5.012	0.074	0.536	1.137E-02	3.894E-03
40	0.0250	4.318E-03	-5.445	0.087	0.590	7.788E-03	2.394E-03
50	0.0200	1.939E-03	-6.245	0.080	0.561	3.398E-03	1.107E-03
55	0.0182	1.161E-03	-6.758	0.081	0.563	2.040E-03	6.612E-04
56	0.0179	1.029E-03	-6.879	0.081	0.562	1.805E-03	5.869E-04
57	0.0175	9.652E-04	-6.943	0.068	0.508	1.604E-03	5.807E-04
58	0.0172	7.897E-04	-7.144	0.087	0.588	1.422E-03	4.387E-04
59	0.0169	6.788E-04	-7.295	0.088	0.591	1.226E-03	3.759E-04
60	0.0167	5.726E-04	-7.465	0.096	0.625	1.070E-03	3.065E-04
61	0.0164	4.730E-04	-7.657	0.101	0.643	8.992E-04	2.488E-04
62	0.0161	3.936E-04	-7.840	0.101	0.642	7.483E-04	2.070E-04
63	0.0159	3.106E-04	-8.077	0.101	0.642	5.905E-04	1.634E-04
64	0.0156	2.213E-04	-8.416	0.137	0.781	4.832E-04	1.014E-04

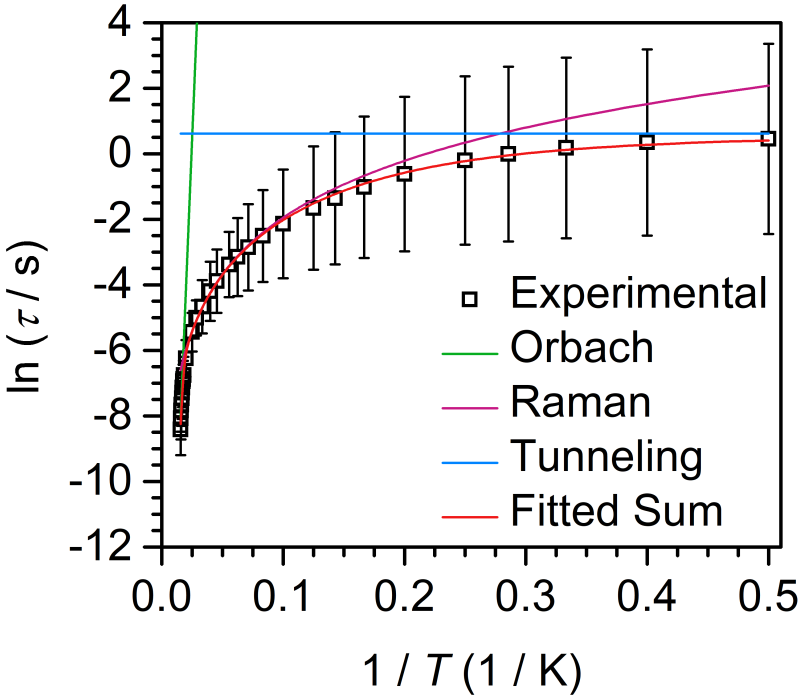

Figure S33. Individual contributions of the multiple magnetic relaxation pathways to the Arrhenius plot of $(NHAr^*)_2DyCl$ (**1**) at 0 Oe with errors to a 1σ limit. The best fit yielded U_{eff} = 626(31) cm^{-1} and τ_0 = 10$^{-9.6(3)}$ s. The red line represents a fit to the sum of an Orbach, a Raman, and a QTM process. Individual parameters used to calculate the contributions are given in Table S6.

DC Relaxation Experiments

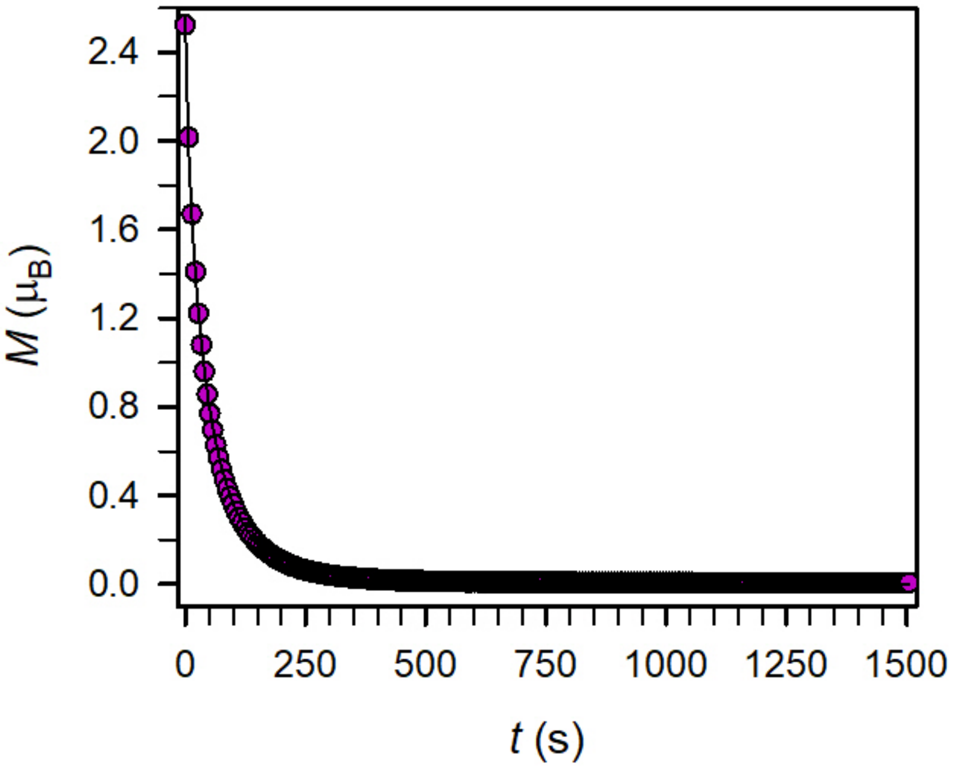

Figure S34. Plot of magnetization vs. time used to derive relaxation times for **2** at 1.8 K. The data (pink circles) were fit to a function of the form $y = a \cdot \exp(-((t/\tau)^b))$ where b is a stretch factor (black line). Decay of the magnetization vs. time for **2**, obtained by applying a magnetic field of 4 T to the sample at a temperature of 1.8 K for 5 min, and then quickly removing the magnetic field.

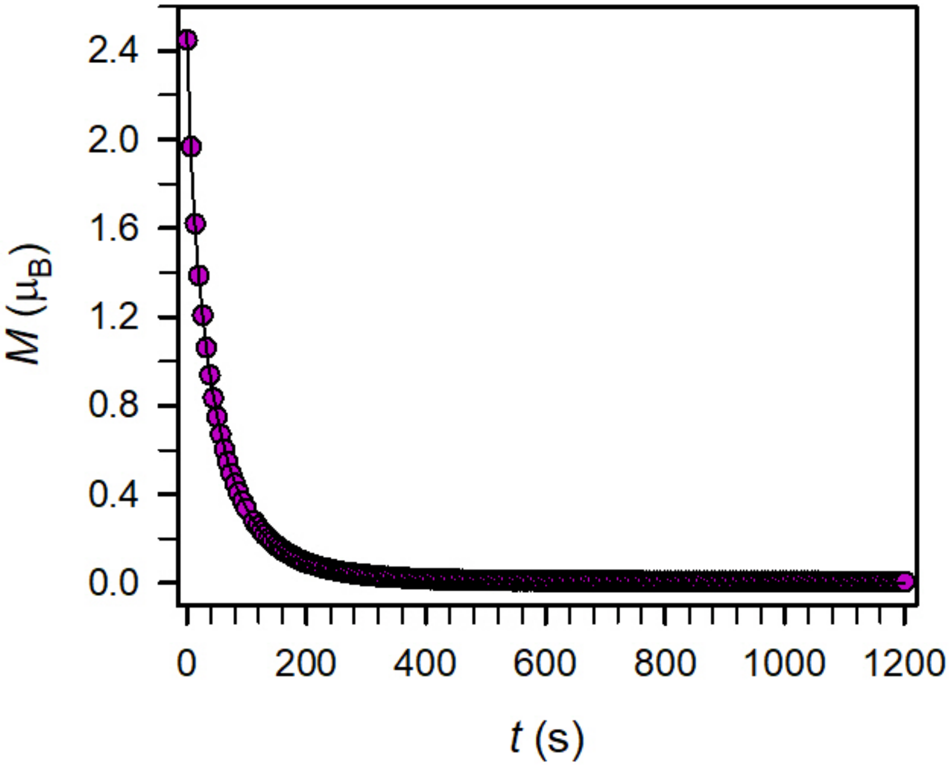

Figure S35. Plot of magnetization vs. time used to derive relaxation times for **2** at 1.9 K. The data (pink circles) were fit to a function of the form $y = a \cdot \exp(-((t/\tau)^b))$ where b is a stretch factor (black line). Decay of the magnetization vs. time for **2**, obtained by applying a magnetic field of 4 T to the sample at a temperature of 1.9 K for 5 min, and then quickly removing the magnetic field.

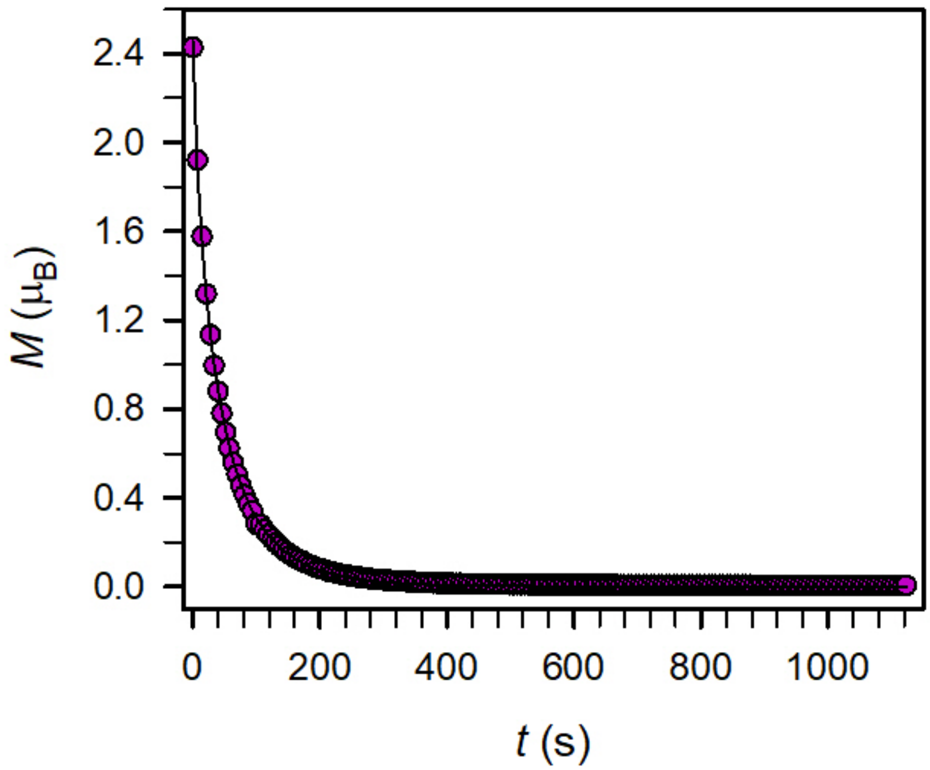

Figure S36. Plot of magnetization vs. time used to derive relaxation times for **2** at 2.0 K. The data (pink circles) were fit to a function of the form $y = a \cdot \exp(-((t/\tau)^b))$ where b is a stretch factor (black line). Decay of the magnetization vs. time for **2**, obtained by applying a magnetic field of 4 T to the sample at a temperature of 2.0 K for 5 min, and then quickly removing the magnetic field.

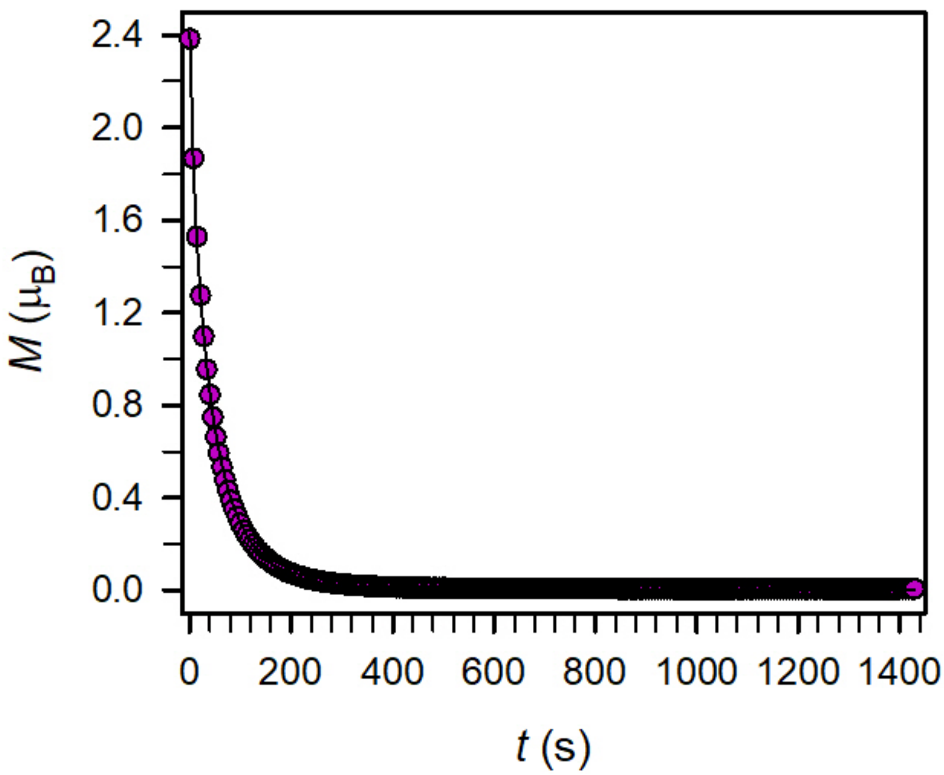

Figure S37. Plot of magnetization vs. time used to derive relaxation times for **2** at 2.1 K. The data (pink circles) were fit to a function of the form $y = a \cdot \exp(-((t/\tau)^b))$ where b is a stretch factor (black line). Decay of the magnetization vs. time for **2**, obtained by applying a magnetic field of 4 T to the sample at a temperature of 2.1 K for 5 min, and then quickly removing the magnetic field.

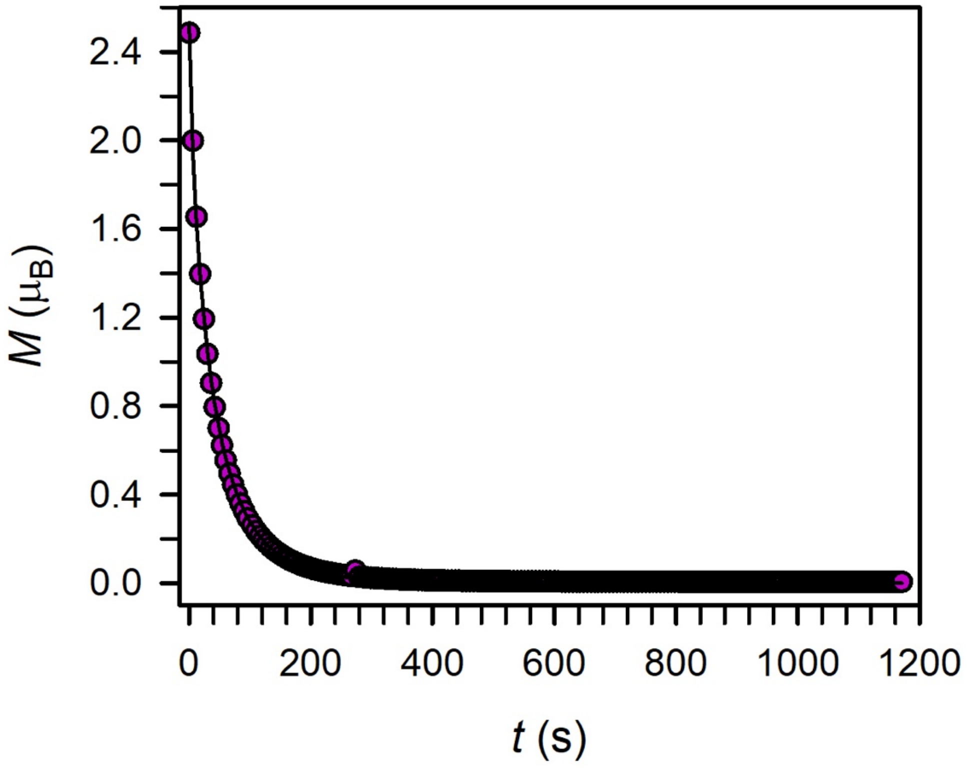

Figure S38. Plot of magnetization vs. time used to derive relaxation times for **2** at 2.2 K. The data (pink circles) were fit to a function of the form $y = a\cdot\exp(-((t/\tau)^b))$ where b is a stretch factor (black line). Decay of the magnetization vs. time for **2**, obtained by applying a magnetic field of 4 T to the sample at a temperature of 2.2 K for 5 min, and then quickly removing the magnetic field.

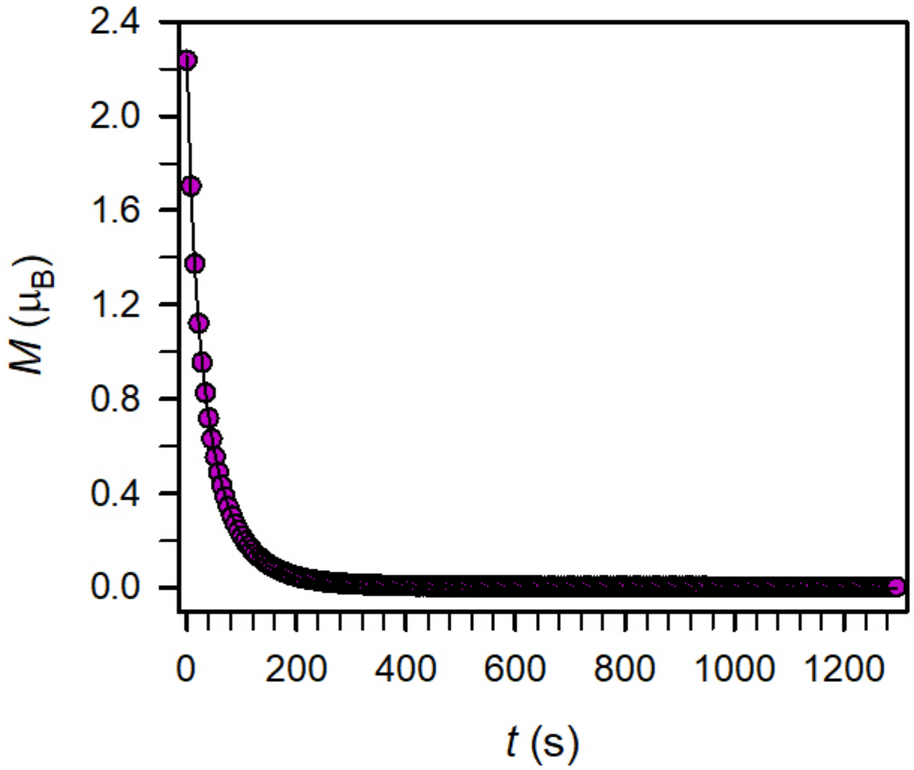

Figure S39. Plot of magnetization vs. time used to derive relaxation times for **2** at 2.5 K. The data (pink circles) were fit to a function of the form $y = a \cdot \exp(-((t/\tau)^b))$ where b is a stretch factor (black line). Decay of the magnetization vs. time for **2**, obtained by applying a magnetic field of 4 T to the sample at a temperature of 2.5 K for 5 min, and then quickly removing the magnetic field.

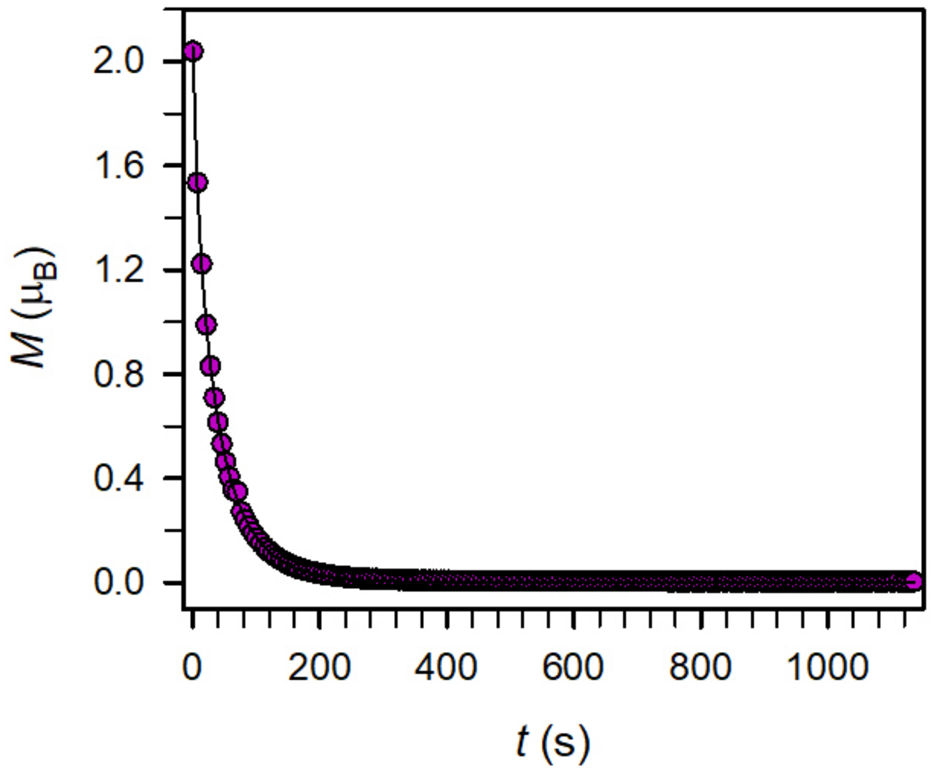

Figure S40. Plot of magnetization vs. time used to derive relaxation times for **2** at 3.0 K. The data (pink circles) were fit to a function of the form $y = a \cdot \exp(-((t/\tau)^b))$ where b is a stretch factor (black line). Decay of the magnetization vs. time for **2**, obtained by applying a magnetic field of 4 T to the sample at a temperature of 3.0 K for 5 min, and then quickly removing the magnetic field.

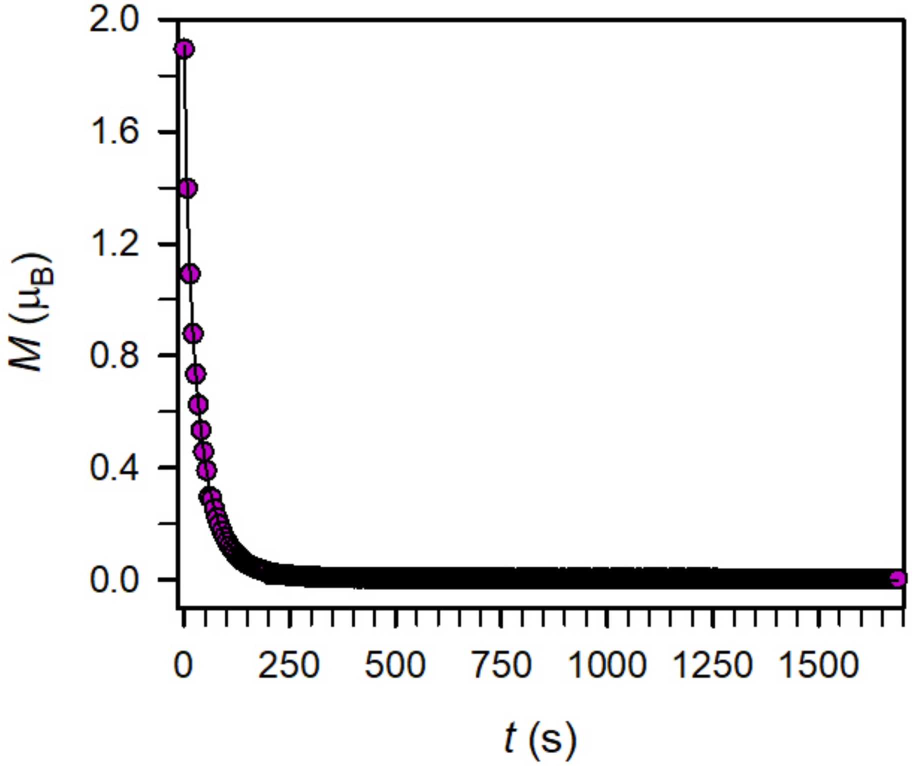

Figure S41. Plot of magnetization vs. time used to derive relaxation times for **2** at 3.5 K. The data (pink circles) were fit to a function of the form $y = a \cdot \exp(-((t/\tau)^b))$ where b is a stretch factor (black line). Decay of the magnetization vs. time for **2**, obtained by applying a magnetic field of 4 T to the sample at a temperature of 3.5 K for 5 min, and then quickly removing the magnetic field.

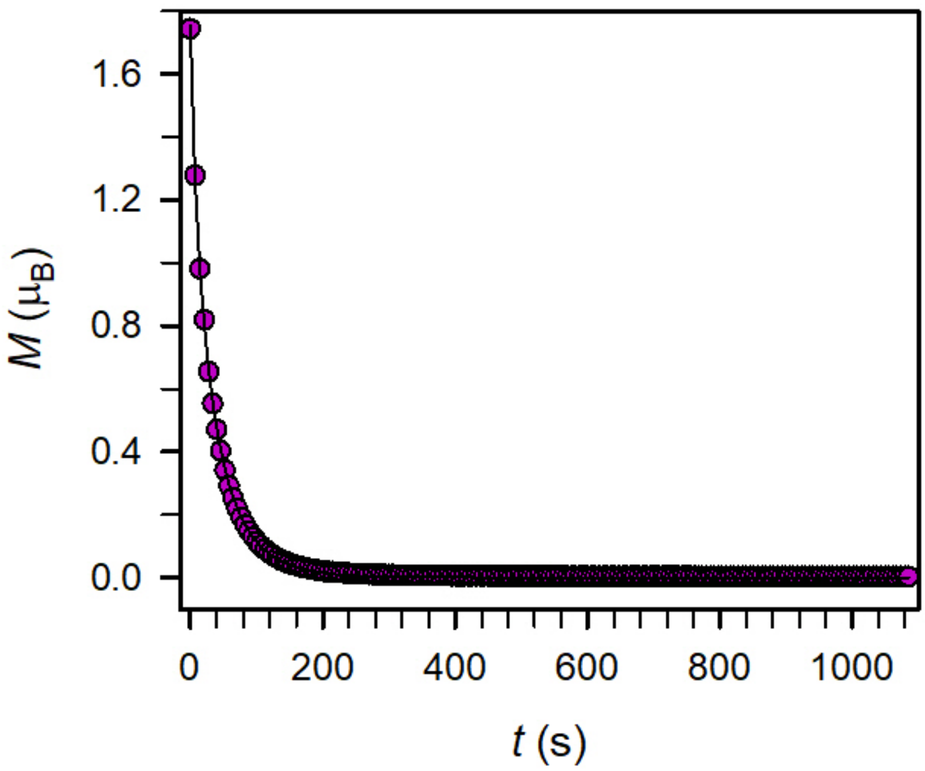

Figure S42. Plot of magnetization vs. time used to derive relaxation times for **2** at 4.0 K. The data (pink circles) were fit to a function of the form $y = a\cdot\exp(-((t/\tau)^b))$ where b is a stretch factor (black line). Decay of the magnetization vs. time for **2**, obtained by applying a magnetic field of 4 T to the sample at a temperature of 4.0 K for 5 min, and then quickly removing the magnetic field.

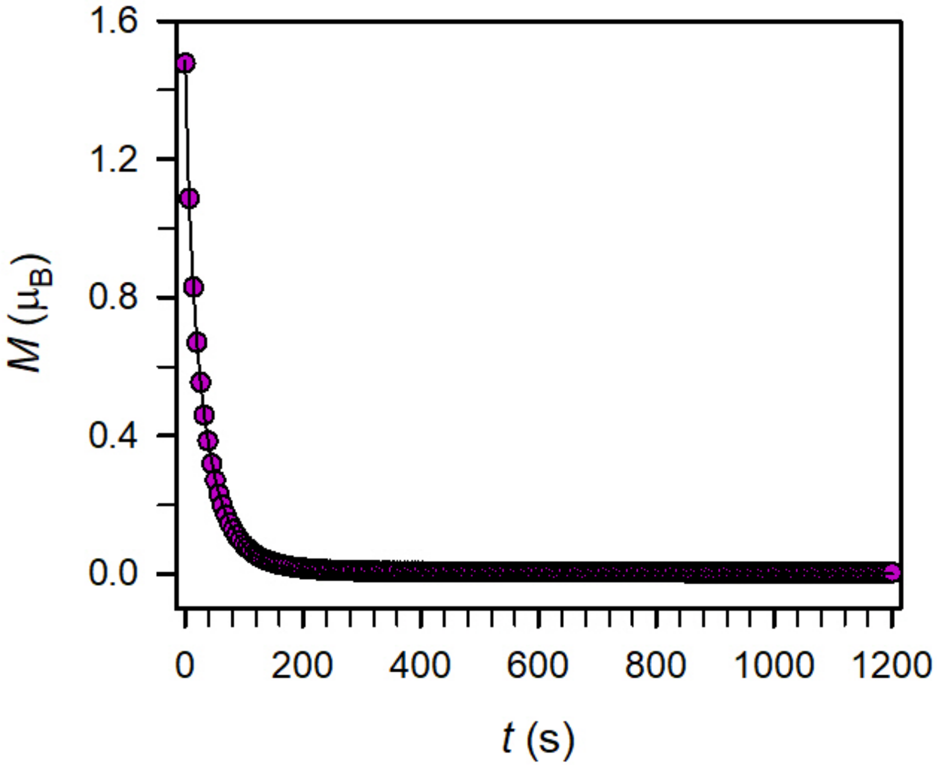

Figure S43. Plot of magnetization vs. time used to derive relaxation times for **2** at 5.0 K. The data (pink circles) were fit to a function of the form $y = a\cdot\exp(-((t/\tau)^b))$ where b is a stretch factor (black line). Decay of the magnetization vs. time for **2**, obtained by applying a magnetic field of 4 T to the sample at a temperature of 5.0 K for 5 min, and then quickly removing the magnetic field.

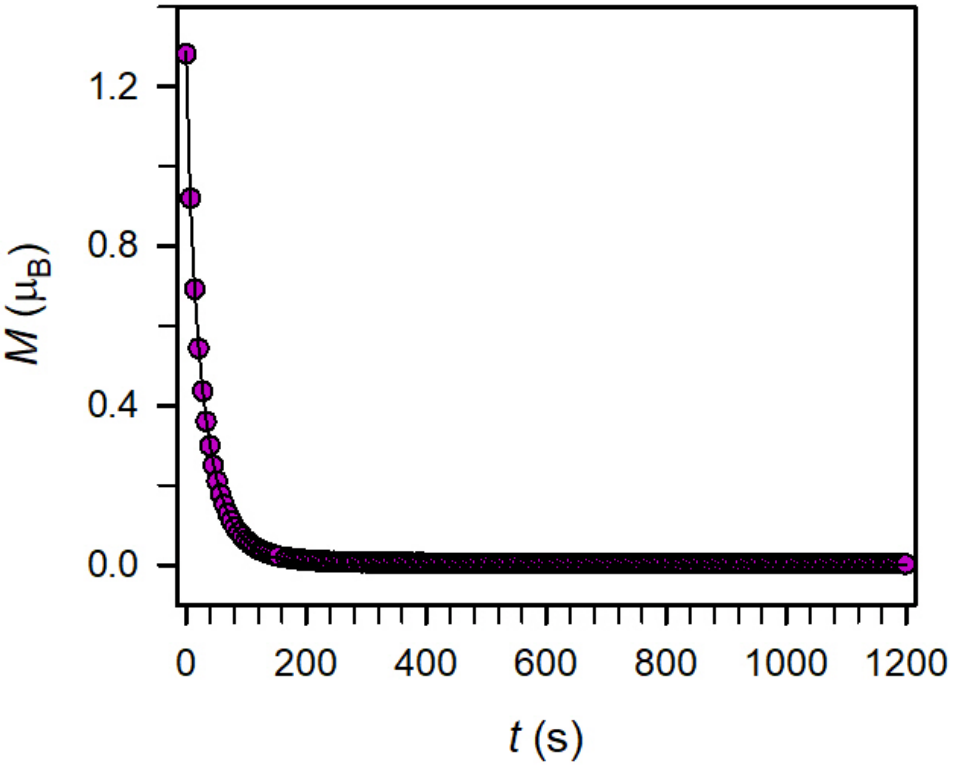

Figure S44. Plot of magnetization vs. time used to derive relaxation times for **2** at 6.0 K. The data (pink circles) were fit to a function of the form $y = a \cdot \exp(-((t/\tau)^b))$ where b is a stretch factor (black line). Decay of the magnetization vs. time for **2**, obtained by applying a magnetic field of 4 T to the sample at a temperature of 6.0 K for 5 min, and then quickly removing the magnetic field.

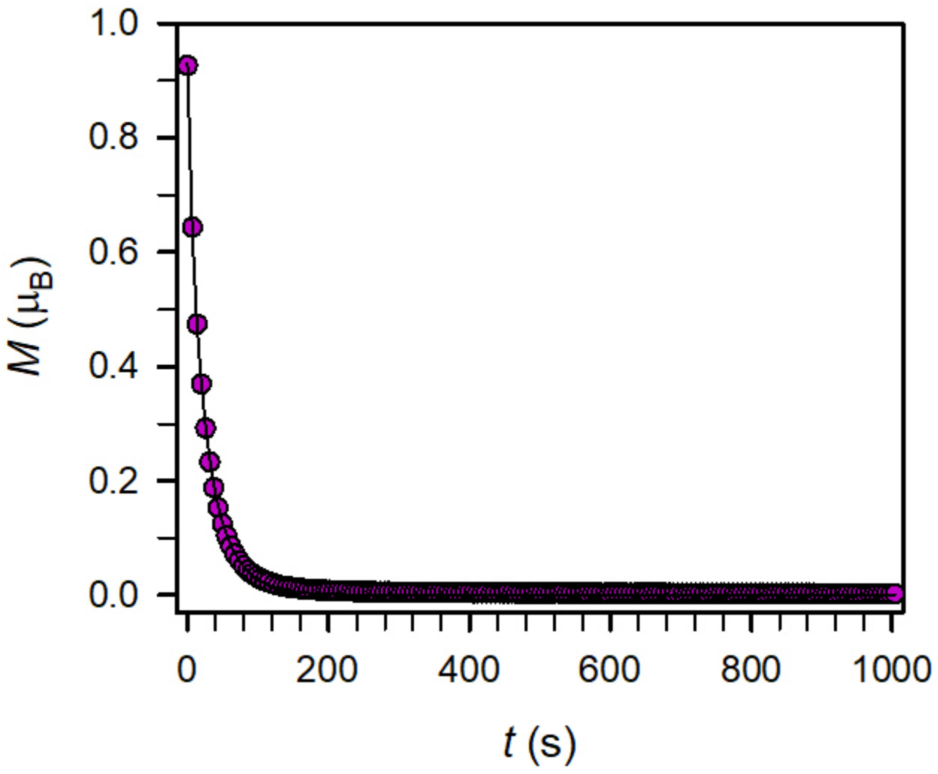

Figure S45. Plot of magnetization vs. time used to derive relaxation times for **2** at 8.0 K. The data (pink circles) were fit to a function of the form $y = a \cdot \exp(-((t/\tau)^b))$ where b is a stretch factor (black line). Decay of the magnetization vs. time for **2**, obtained by applying a magnetic field of 4 T to the sample at a temperature of 8.0 K for 5 min, and then quickly removing the magnetic field.

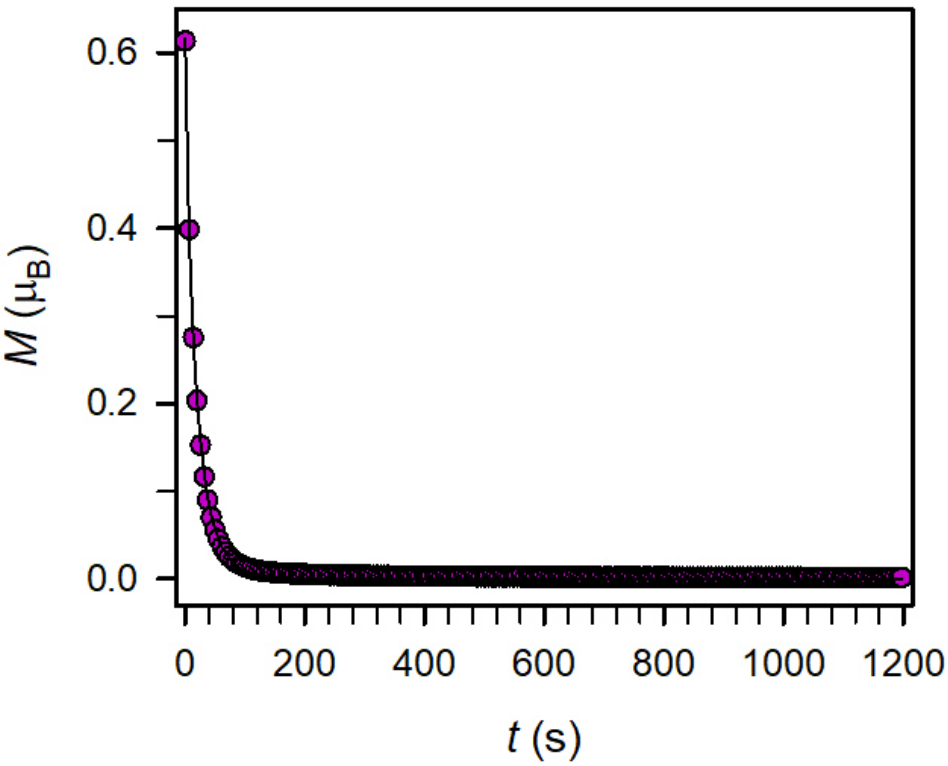

Figure S46. Plot of magnetization vs. time used to derive relaxation times for **2** at 10.0 K. The data (pink circles) were fit to a function of the form $y = a \cdot \exp(-((t/\tau)^b))$ where b is a stretch factor (black line). Decay of the magnetization vs. time for **2**, obtained by applying a magnetic field of 4 T to the sample at a temperature of 10.0 K for 5 min, and then quickly removing the magnetic field.

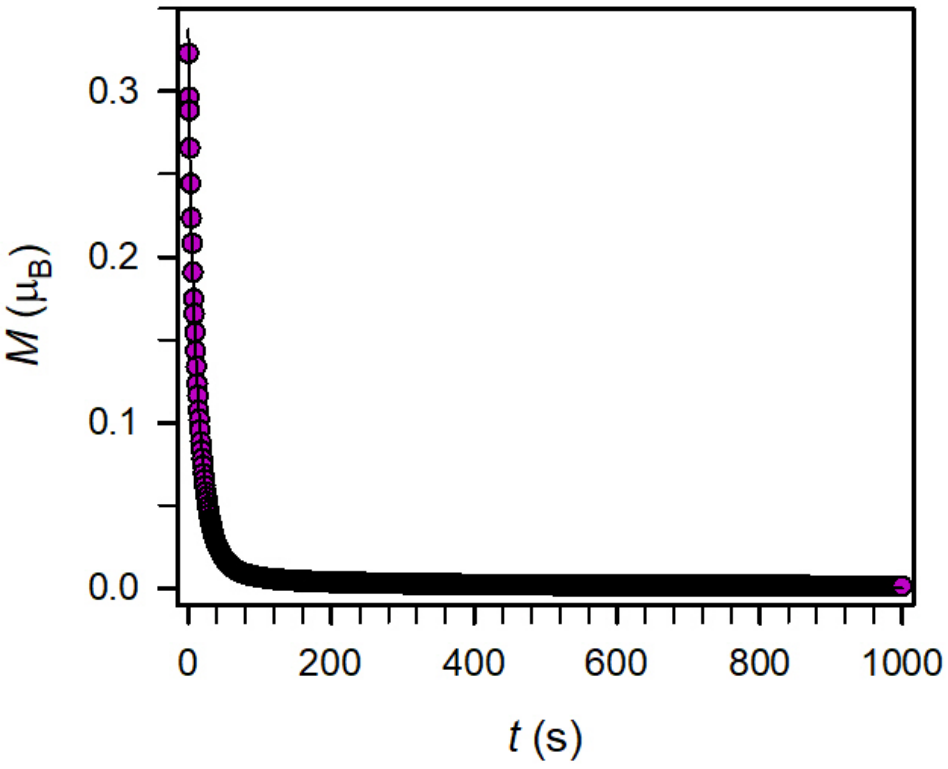

Figure S47. Plot of magnetization vs. time used to derive relaxation times for **2** at 12.0 K. The data (pink circles) were fit to a function of the form $y = a \cdot \exp(-((t/\tau)^b))$ where b is a stretch factor (black line). Decay of the magnetization vs. time for **2**, obtained by applying a magnetic field of 4 T to the sample at a temperature of 12.0 K for 5 min, and then quickly removing the magnetic field.

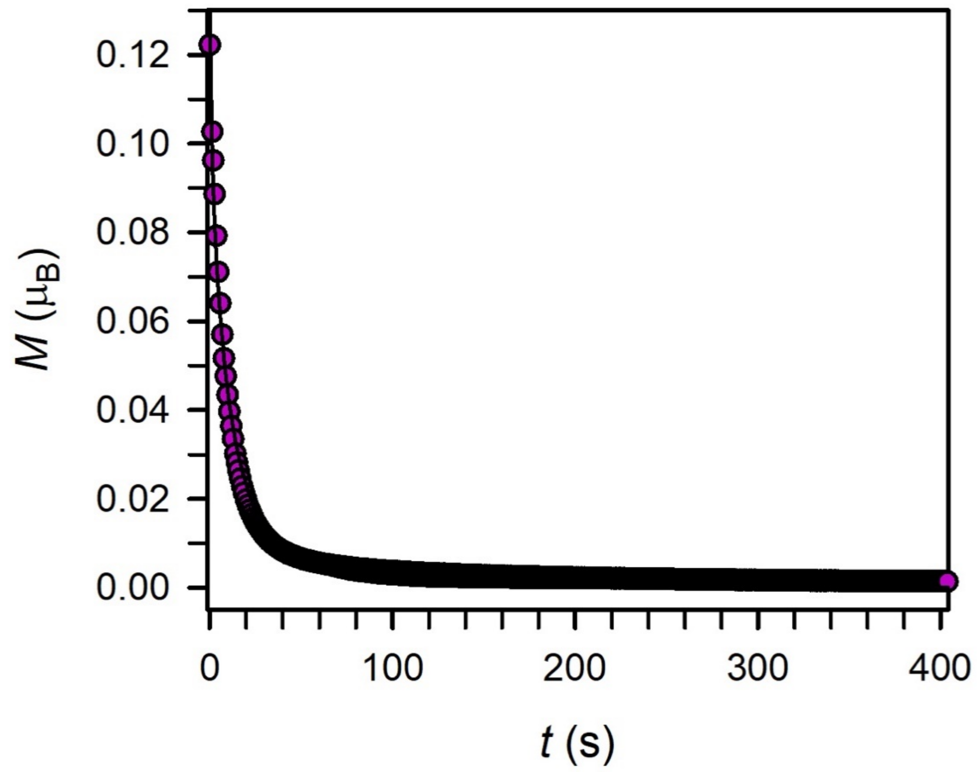

Figure S48. Plot of magnetization vs. time used to derive relaxation times for **2** at 14.0 K. The data (pink circles) were fit to a function of the form $y = a \cdot \exp(-((t/\tau)^b))$ where b is a stretch factor (black line). Decay of the magnetization vs. time for **2**, obtained by applying a magnetic field of 4 T to the sample at a temperature of 14.0 K for 5 min, and then quickly removing the magnetic field.

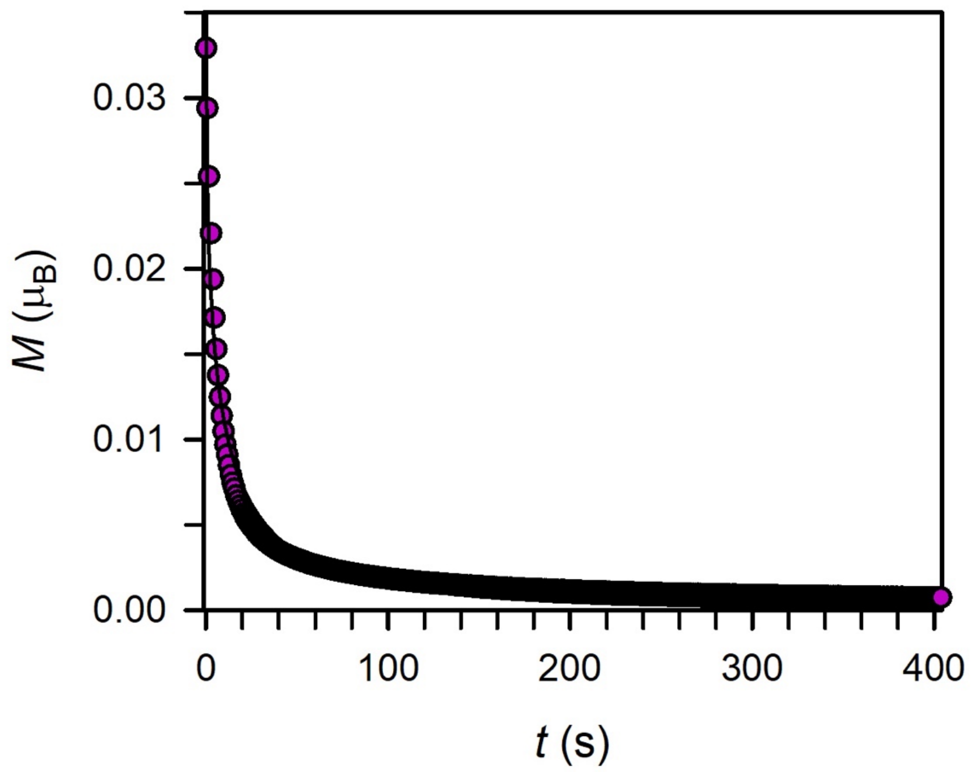

Figure S49. Plot of magnetization vs. time used to derive relaxation times for **2** at 16.0 K. The data (pink circles) were fit to a function of the form $y = a \cdot \exp(-((t/\tau)^b))$ where b is a stretch factor (black line). Decay of the magnetization vs. time for **2**, obtained by applying a magnetic field of 4 T to the sample at a temperature of 16.0 K for 5 min, and then quickly removing the magnetic field.

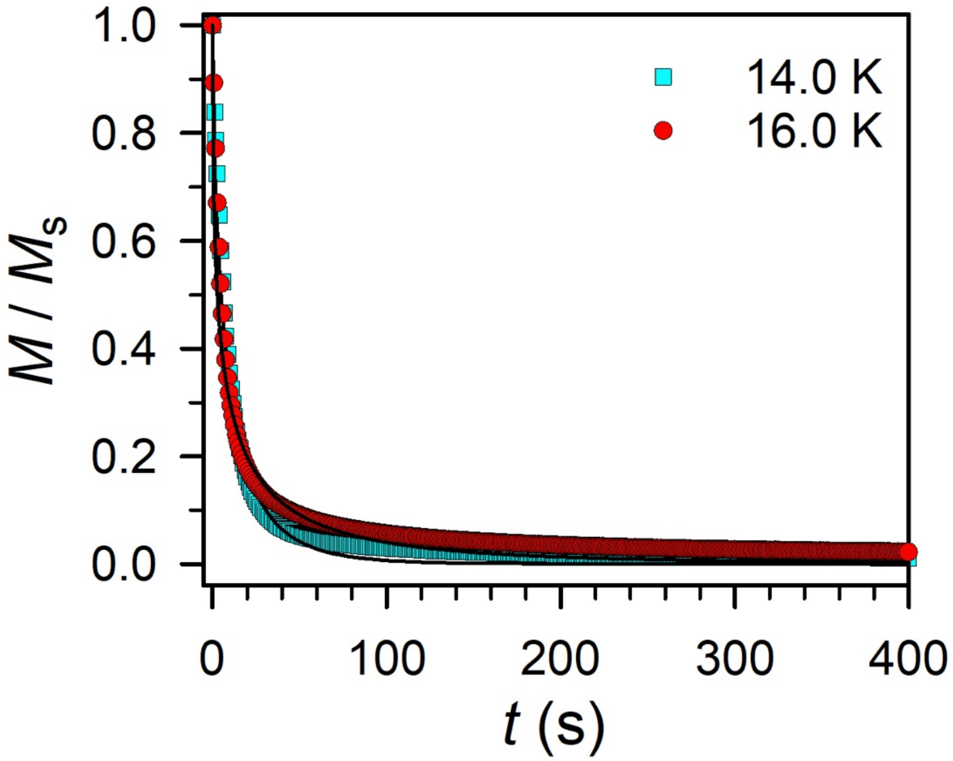

Figure S50. Plot of magnetization (normalized) vs. time used to derive relaxation times for **2** at 14 and 16 K. The data were fit to a function of the form $y = a\cdot\exp(-((t/\tau)^b))$ where b is a stretch factor (black line). Decay of the magnetization vs. time for **2**, obtained by applying a magnetic field of 4 T to the sample at a given temperature for 5 min, and then quickly removing the magnetic field.

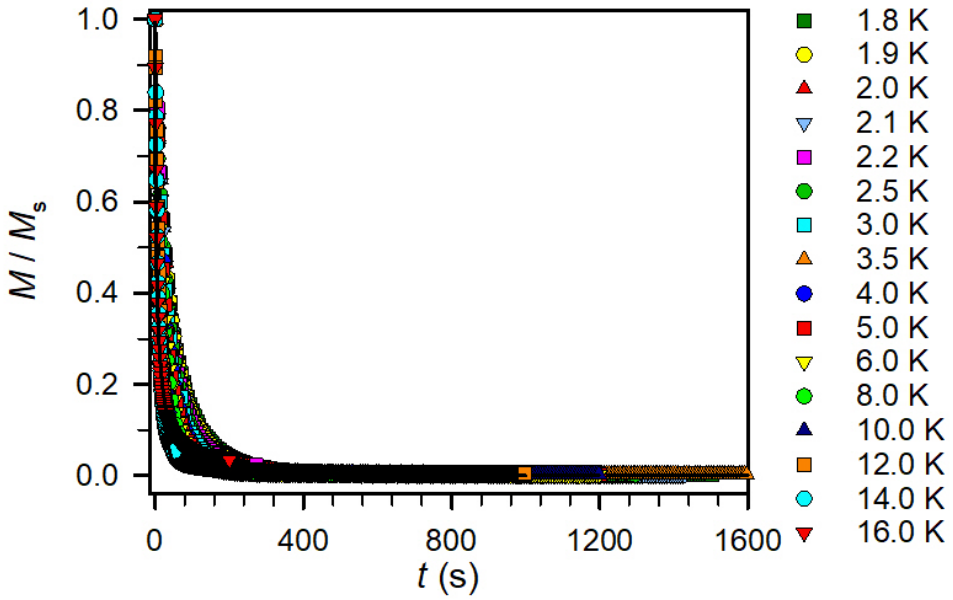

Figure S51. Plot of magnetization (normalized) vs. time used to derive relaxation times for **2** at different temperatures from 1.8 to 16 K. The data were fit to a function of the form $y = a \cdot \exp(-((t/\tau)^b))$ where b is a stretch factor (black line). Decay of the magnetization vs. time for **2**, obtained by applying a magnetic field of 4 T to the sample at a given temperature for 5 min, and then quickly removing the magnetic field.

Table S4. Pre-exponential factor, *a*, relaxation times, τ (s), and stretch factors, *b*, at various temperatures, *T* (K), for **2**.

***T* (K)**	**pre-exponential factor, *a***	**τ (s)**	**stretch factor, *b***
1.8	2.559	41.287	0.753
1.9	2.481	40.174	0.766
2.0	2.456	38.728	0.766
2.1	2.411	37.859	0.771
2.2	2.519	35.824	0.770
2.5	2.254	33.933	0.779
3.0	2.052	31.624	0.784
3.5	1.907	29.569	0.792
4.0	1.752	28.756	0.804
5.0	1.486	26.829	0.808
6.0	1.287	25.231	0.820
8.0	0.931	21.742	0.826
10.0	0.616	17.647	0.830
12.0	0.338	12.465	0.784
14.0	0.127	9.290	0.757
16.0	0.037	6.823	0.445

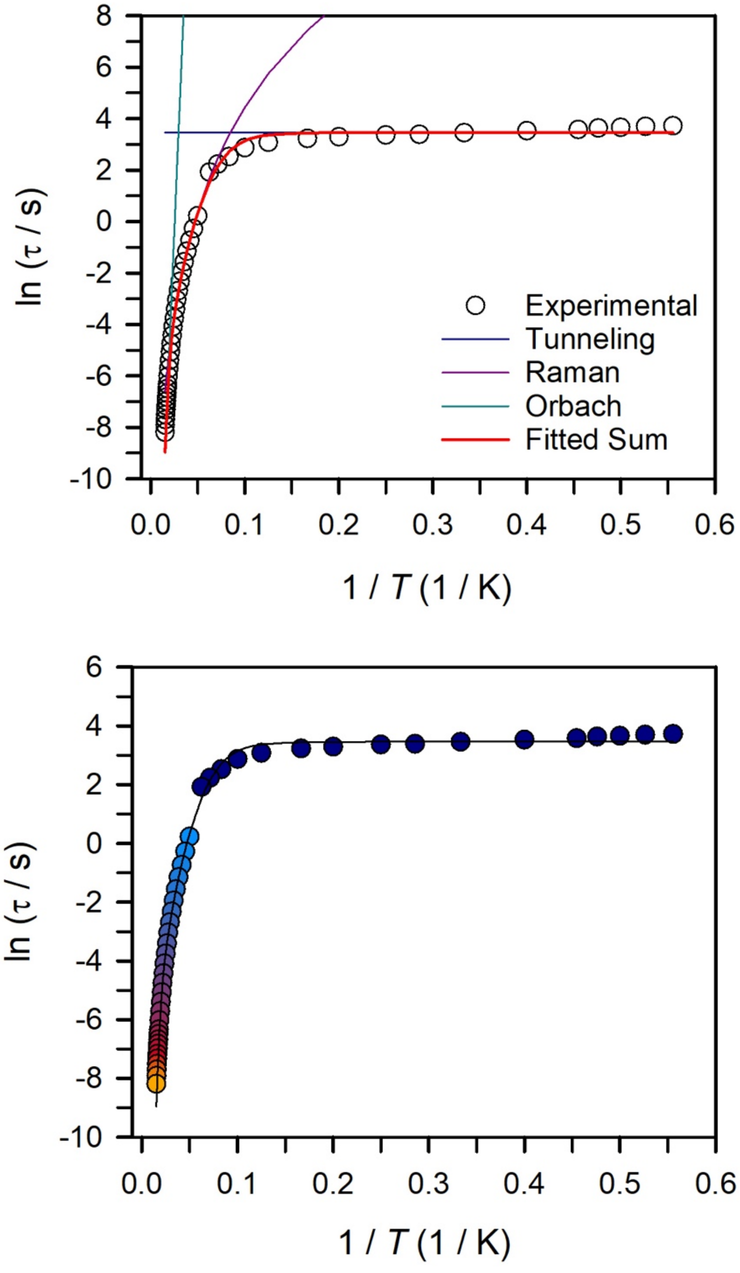

Figure S52. (top) Individual contributions of the multiple magnetic relaxation pathways to the Arrhenius plot of **2** at 0 Oe. Individual parameters used to calculate the contributions are given Table 1 (main text). (bottom) Plot of natural log of the relaxation time versus the inverse temperature (temperature range 1.8 to 66 K) for **2**. Pale blue to red circles represent data extracted from ac magnetic susceptibility measurements, and dark blue circles represent data extracted from dc relaxation experiments. The black line represents a fit to a Orbach relaxation process, a Raman relaxation process and a quantum tunneling pathway as described in Figure 8.

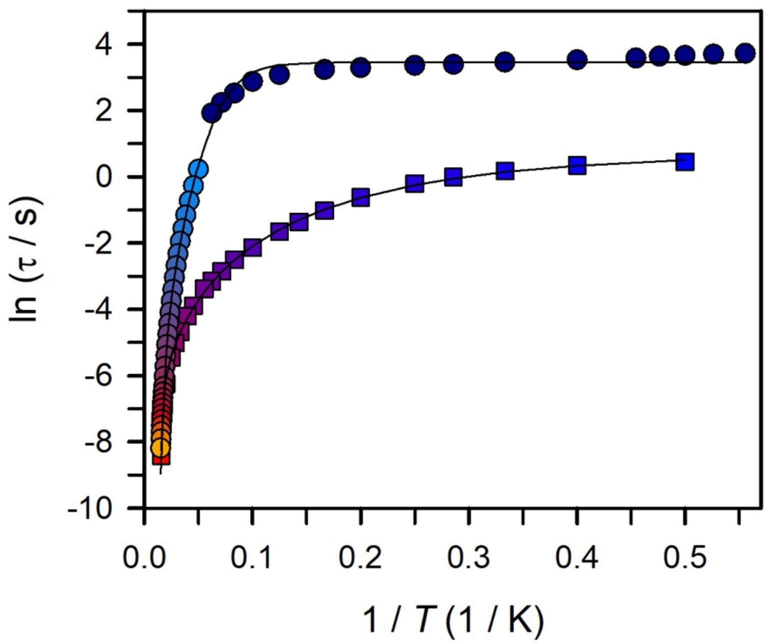

Figure S53. Plot of natural log of the relaxation time versus the inverse temperature for **1** (temperature range 2 to 64 K) and for **2** (temperature range 1.8 to 66 K). For **1**: Blue to red squares represent data extracted from ac magnetic susceptibility measurements. For **2**: Pale blue to red circles represent data extracted from ac magnetic susceptibility measurements, and dark blue circles represent data extracted from dc relaxation experiments. Each black line represents a fit to a Orbach relaxation process, a Raman relaxation process and a quantum tunneling pathway as described in Figure 8, and Table S4.

Table S5. Summary of relaxations times of $[(NHAr^*)_2Dy][BArF_{24}]$ (**2**), either obtained from fitting the Cole-Cole plots for ac magnetic susceptibility to a generalized Debye model or from fitting magnetization decay curves to a stretched exponential function. Upper- and lower τ bounds were calculated according to a 1σ model. Data in green and blue were extracted from ac susceptibility measurements and dc relaxation experiments, respectively.

T (K)	T^{-1} (K^{-1})	τ (s)	ln(τ) (ln(s))	α	b	σ ln(τ) (ln(s))	τ_{upper} (s)	τ_{lower} (s)
1.8	0.5556	4.129 E+01	3.721	-	0.753	1.121	1.266 E+02	1.346 E+01
1.9	0.5263	4.017 E+01	3.693	-	0.766	1.077	1.180 E+02	1.368 E+01
2	0.5000	3.873 E+01	3.657	-	0.766	1.075	1.135 E+02	1.321 E+01
2.1	0.4762	3.786 E+01	3.634	-	0.771	1.060	1.093 E+02	1.311 E+01
2.2	0.4545	3.582 E+01	3.579	-	0.770	1.062	1.036 E+02	1.239 E+01

2.5	0.4000	3.393 E+01	3.524	-	0.779	1.032	9.527 E+01	1.209 E+01
3	0.3333	3.162 E+01	3.454	-	0.784	1.017	8.741 E+01	1.144 E+01
3.5	0.2857	2.957 E+01	3.387	-	0.792	0.990	7.955 E+01	1.099 E+01
4	0.2500	2.876 E+01	3.359	-	0.804	0.950	7.432 E+01	1.113 E+01
5	0.2000	2.683 E+01	3.289	-	0.808	0.935	6.835 E+01	1.053 E+01
6	0.1667	2.523 E+01	3.228	-	0.820	0.894	6.171 E+01	1.032 E+01
8	0.1250	2.174 E+01	3.079	-	0.826	0.875	5.217 E+01	9.060 E+00
10	0.1000	1.765 E+01	2.871	-	0.830	0.861	4.175 E+01	7.459 E+00
12	0.0833	1.246 E+01	2.523	-	0.784	1.016	3.444 E+01	4.511 E+00
14	0.0714	9.290 E+00	2.229	-	0.757	1.107	2.810 E+01	3.071 E+00
16	0.0625	6.823 E+00	1.920	-	0.445	2.582	9.019 E+01	5.162 E-01
20	0.0500	1.251 E+00	0.224	0.055	-	0.450	1.964 E+00	7.976 E-01
22	0.0455	7.589 E-01	-0.276	0.048	-	0.417	1.151 E+00	5.002 E-01
24	0.0417	4.808 E-01	-0.732	0.040	-	0.379	7.024 E-01	3.290 E-01
26	0.0385	3.147 E-01	-1.156	0.039	-	0.372	4.563 E-01	2.170 E-01
28	0.0357	2.090 E-01	-1.565	0.036	-	0.358	2.988 E-01	1.462 E-01
30	0.0333	1.425 E-01	-1.949	0.034	-	0.348	2.018 E-01	1.006 E-01
32	0.0313	9.770 E-02	-2.326	0.032	-	0.335	1.366 E-01	6.990 E-02
34	0.0294	6.822 E-02	-2.685	0.026	-	0.301	9.219 E-02	5.048 E-02
36	0.0278	4.784 E-02	-3.040	0.040	-	0.380	6.997 E-02	3.271 E-02
38	0.0263	3.317 E-02	-3.406	0.029	-	0.316	4.550 E-02	2.417 E-02
40	0.0250	2.349 E-02	-3.751	0.033	-	0.345	3.316 E-02	1.665 E-02
42	0.0238	1.677 E-02	-4.088	0.038	-	0.367	2.420 E-02	1.162 E-02

44	0.0227	1.196 E-02	-4.426	0.040	-	0.377	1.744 E-02	8.206 E-03
46	0.0217	8.657 E-03	-4.749	0.036	-	0.359	1.239 E-02	6.046 E-03
48	0.0208	6.294 E-03	-5.068	0.027	-	0.309	8.571 E-03	4.622 E-03
50	0.0200	4.522 E-03	-5.399	0.041	-	0.387	6.657 E-03	3.071 E-03
52	0.0192	3.285 E-03	-5.719	0.045	-	0.406	4.931 E-03	2.188 E-03
54	0.0185	2.428 E-03	-6.021	0.029	-	0.319	3.341 E-03	1.765 E-03
56	0.0179	1.767 E-03	-6.338	0.034	-	0.347	2.500 E-03	1.250 E-03
57	0.0175	1.503 E-03	-6.501	0.042	-	0.389	2.218 E-03	1.018 E-03
58	0.0172	1.767 E-03	-6.338	0.034	-	0.347	2.500 E-03	1.250 E-03
59	0.0169	1.083 E-03	-6.828	0.044	-	0.401	1.617 E-03	7.251 E-04
60	0.0167	9.244 E-04	-6.986	0.044	-	0.401	1.380 E-03	6.190 E-04
61	0.0164	7.692 E-04	-7.170	0.048	-	0.421	1.171 E-03	5.051 E-04
62	0.0161	6.574 E-04	-7.327	0.045	-	0.404	9.843 E-04	4.390 E-04
63	0.0159	5.441 E-04	-7.516	0.054	-	0.445	8.490 E-04	3.487 E-04
64	0.0156	4.503 E-04	-7.706	0.062	-	0.485	7.311 E-04	2.773 E-04
65	0.0154	3.637 E-04	-7.919	0.074	-	0.535	6.208 E-04	2.130 E-04
66	0.0152	2.767 E-04	-8.193	0.099	-	0.637	5.231 E-04	1.464 E-04

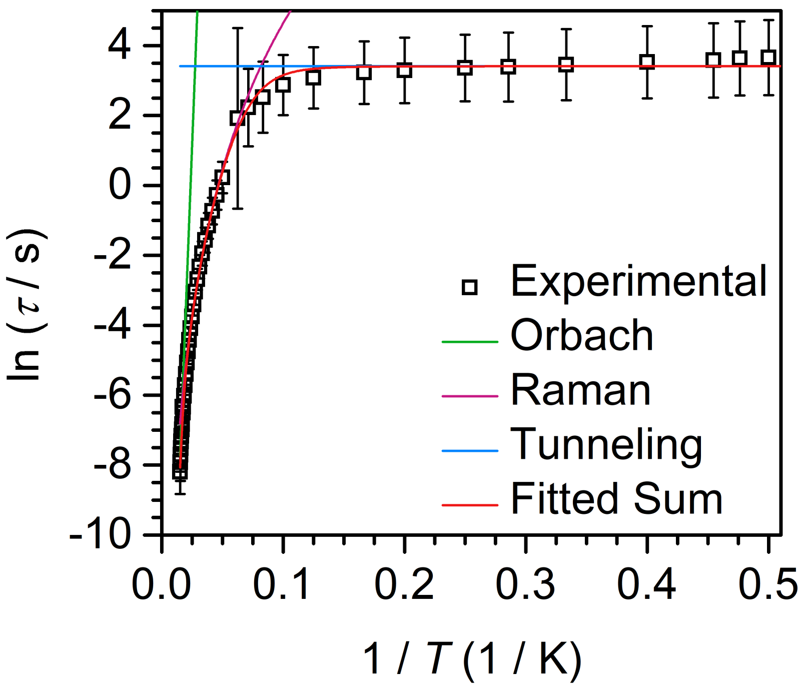

Figure S54. Individual contributions of the multiple magnetic relaxation pathways to the Arrhenius plot of $[(NHAr^*)_2Dy][BArF_{24}]$ (**2**) at 0 Oe with errors to a 1σ limit. The best fit yielded U_{eff} = 625(2) cm^{-1} and τ_0 = 10$^{-9.28(3)}$ s. The red lines represent fits to the sum of an Orbach, a Raman, and a QTM process. Individual parameters used to calculate the contributions are given in Table S6.

Table S6. Summary of fitted Arrhenius plots of $(NHAr^*)_2DyCl$ (**1**) and $[(NHAr^*)_2Dy][BArF_{24}]$ (**2**) to a 1σ model.

Compound	U_{eff} (cm^{-1})	τ_0 (s)	C (s^{-1}K^{-n})	n	τ_{QTM} (s)
**1**	626(31)	$10^{-9.6(3)}$	$10^{-1.66(8)}$	2.50(5)	$10^{0.27(9)}$
**2**	625(2)	$10^{-9.28(3)}$	$10^{-8.1(1)}$	6.07(8)	$10^{1.48(3)}$

Variable-Field Magnetization Measurements

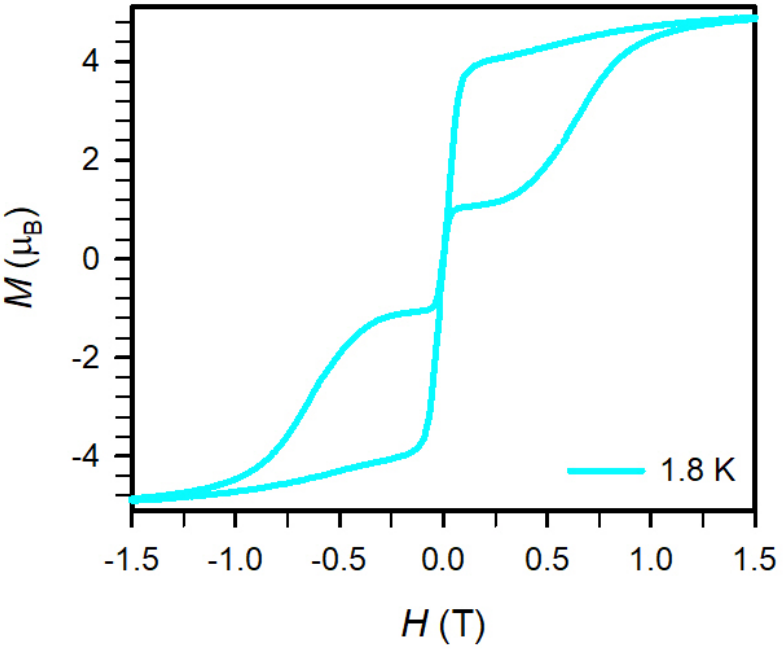

Figure S55. Plot of magnetization (*M*) vs dc magnetic field (*H*) at an average sweep rate of 100 Oe/s for **1** at 1.8 K. Solid lines are guides for the eye.

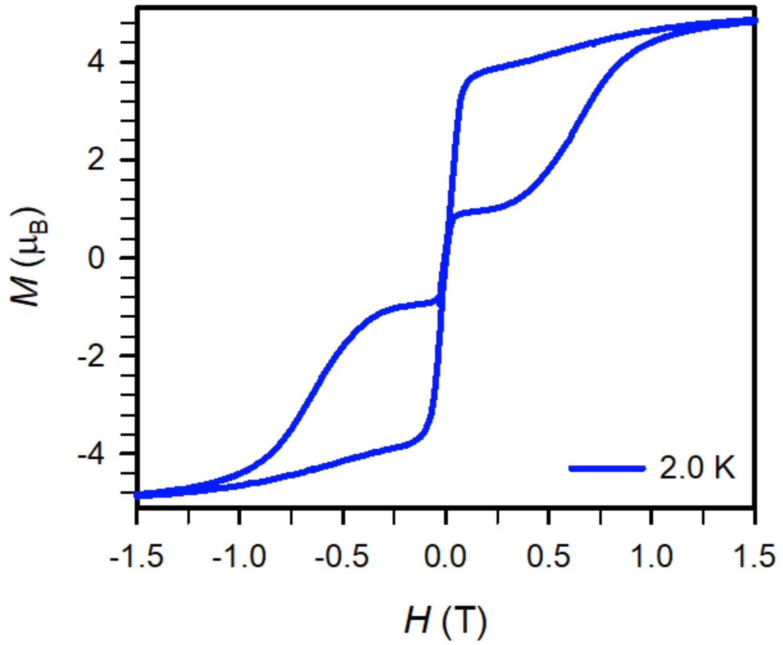

Figure S56. Plot of magnetization (*M*) vs dc magnetic field (*H*) at an average sweep rate of 100 Oe/s for **1** at 2.0 K. Solid lines are guides for the eye.

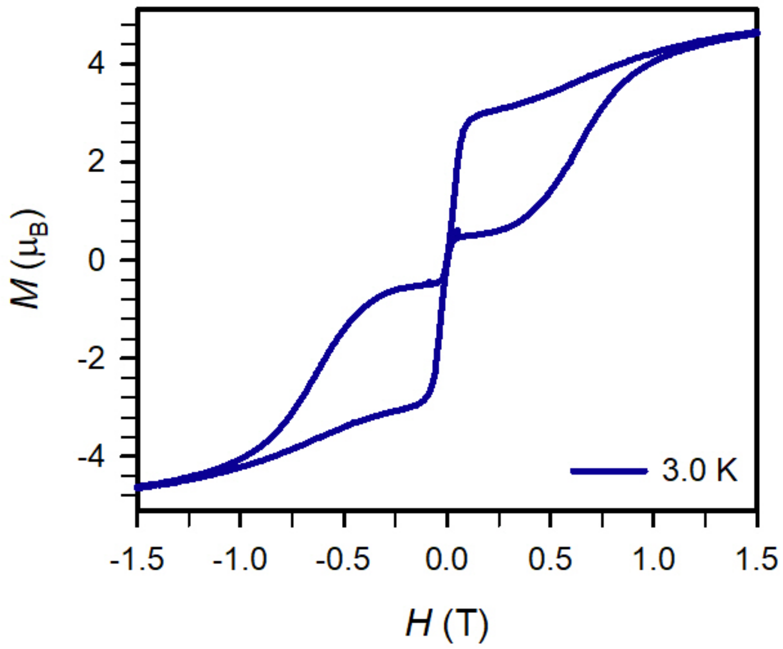

Figure S57. Plot of magnetization (*M*) vs dc magnetic field (*H*) at an average sweep rate of 100 Oe/s for **1** at 3.0 K. Solid lines are guides for the eye.

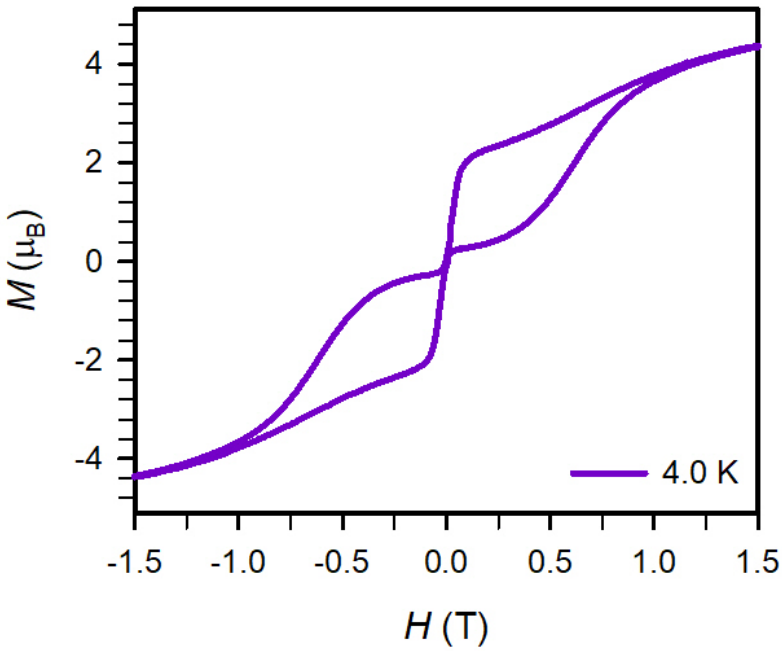

Figure S58. Plot of magnetization (*M*) vs dc magnetic field (*H*) at an average sweep rate of 100 Oe/s for **1** at 4.0 K. Solid lines are guides for the eye.

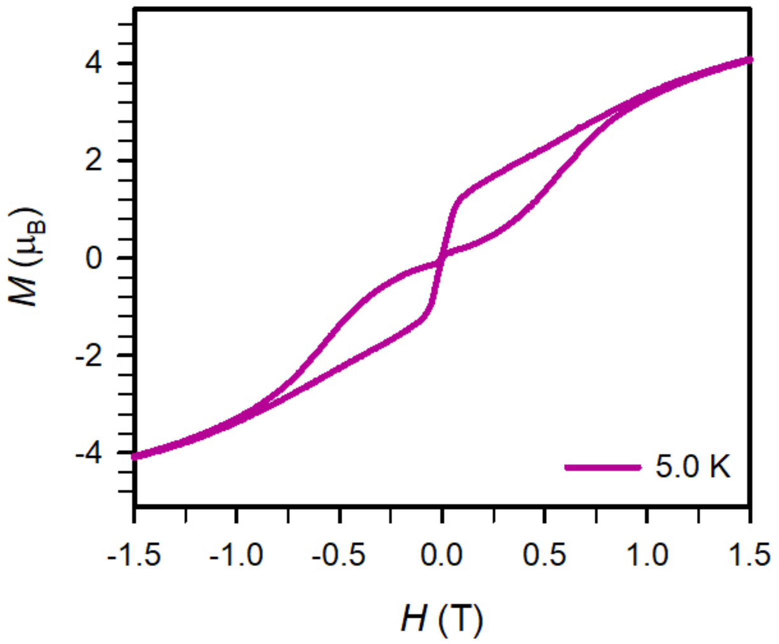

Figure S59. Plot of magnetization (*M*) vs dc magnetic field (*H*) at an average sweep rate of 100 Oe/s for **1** at 5.0 K. Solid lines are guides for the eye.

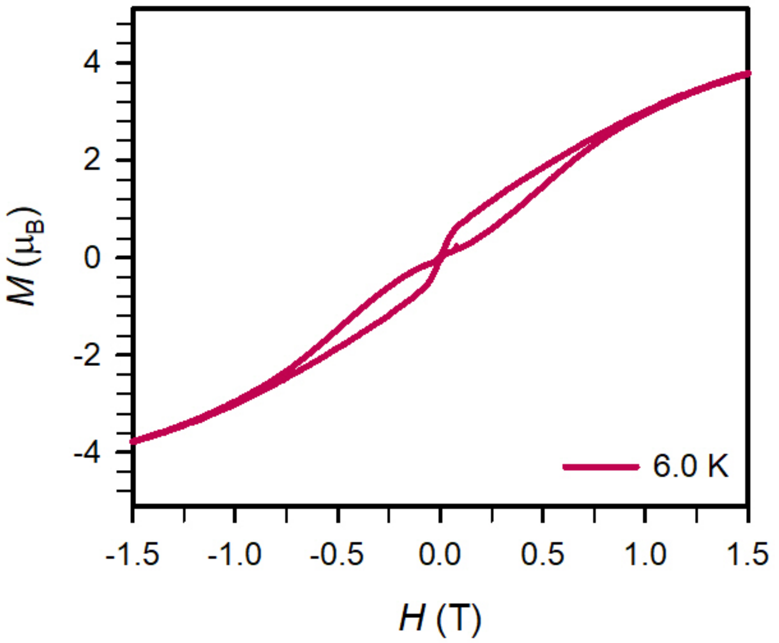

Figure S60. Plot of magnetization (*M*) vs dc magnetic field (*H*) at an average sweep rate of 100 Oe/s for **1** at 6.0 K. Solid lines are guides for the eye.

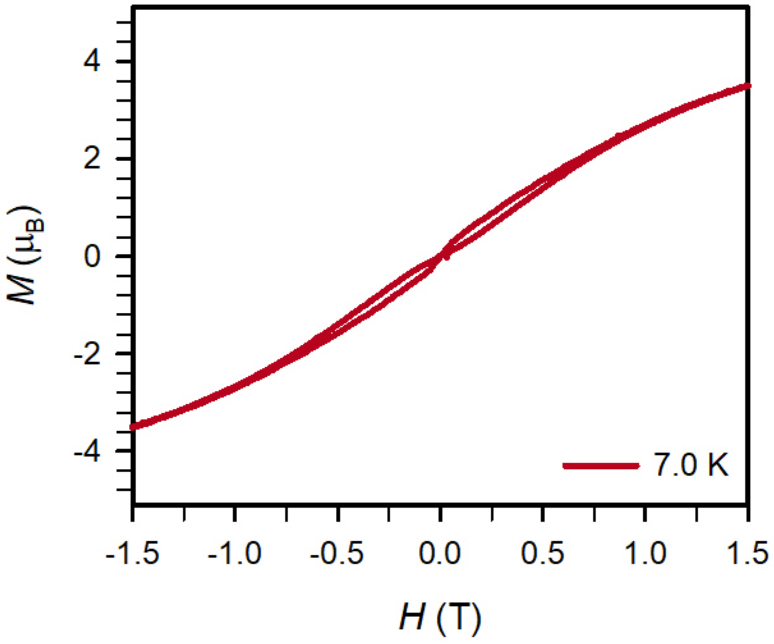

Figure S61. Plot of magnetization (*M*) vs dc magnetic field (*H*) at an average sweep rate of 100 Oe/s for **1** at 7.0 K. Solid lines are guides for the eye.

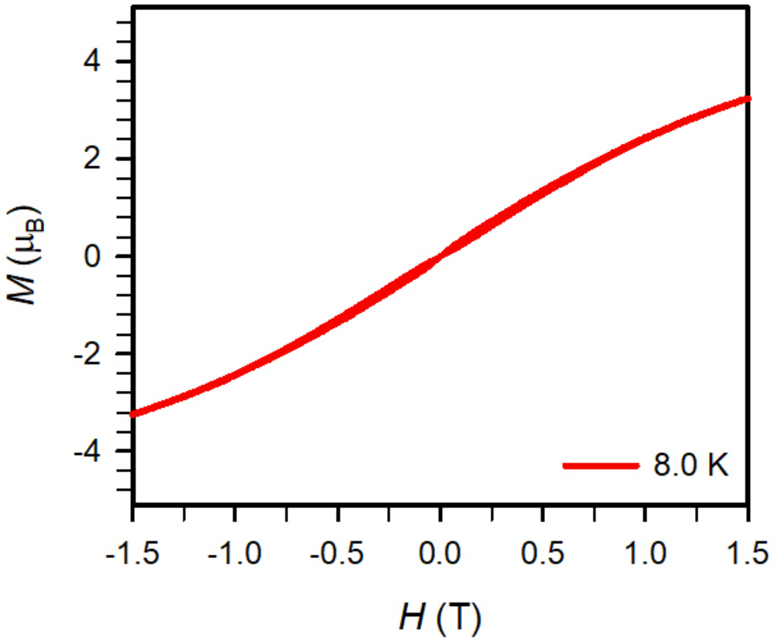

Figure S62. Plot of magnetization (*M*) vs dc magnetic field (*H*) at an average sweep rate of 100 Oe/s for **1** at 8.0 K. Solid lines are guides for the eye.

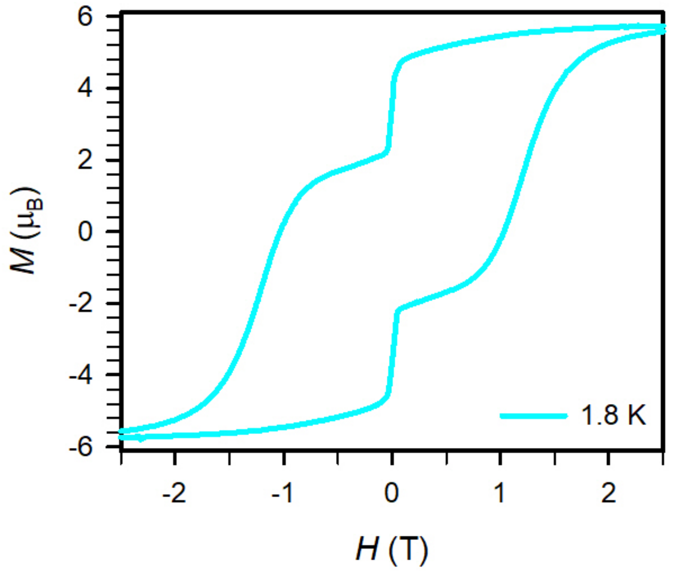

Figure S63. Plot of magnetization (*M*) vs dc magnetic field (*H*) at an average sweep rate of 100 Oe/s for **2** at 1.8 K. Solid lines are guides for the eye.

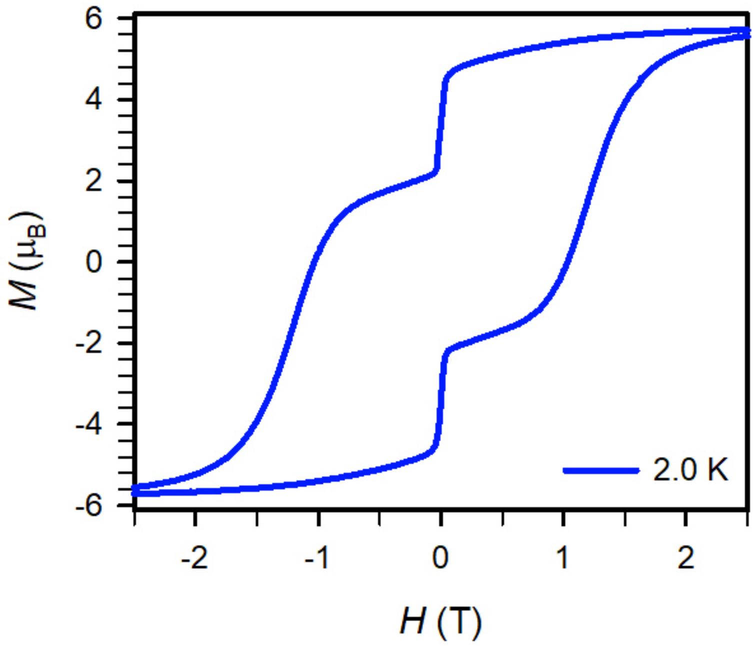

Figure S64. Plot of magnetization (*M*) vs dc magnetic field (*H*) at an average sweep rate of 100 Oe/s for **2** at 2.0 K. Solid lines are guides for the eye.

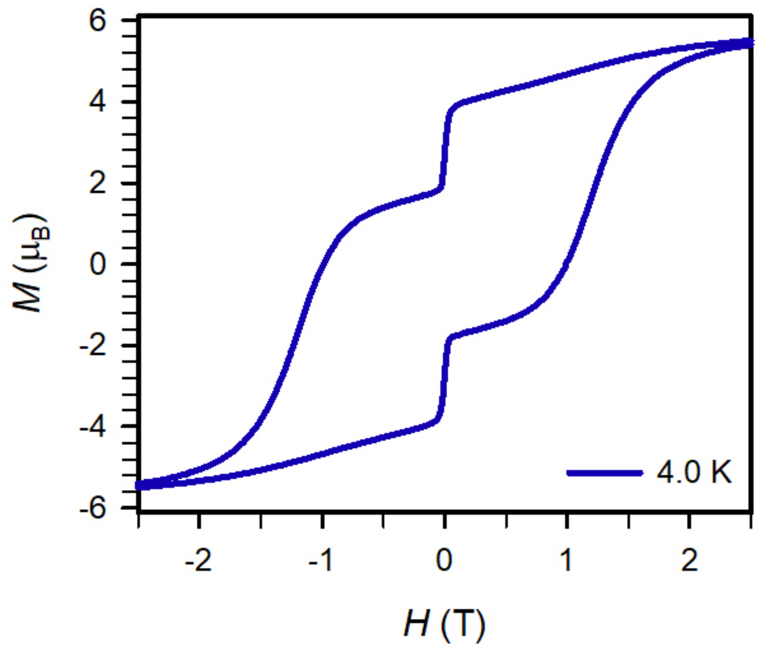

Figure S65. Plot of magnetization (*M*) vs dc magnetic field (*H*) at an average sweep rate of 100 Oe/s for **2** at 4.0 K. Solid lines are guides for the eye.

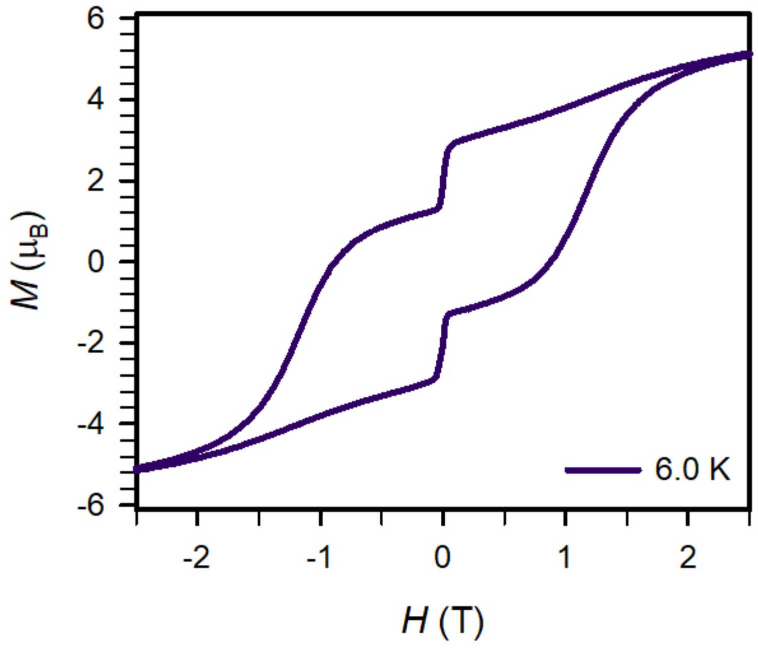

Figure S66. Plot of magnetization (*M*) vs dc magnetic field (*H*) at an average sweep rate of 100 Oe/s for **2** at 6.0 K. Solid lines are guides for the eye.

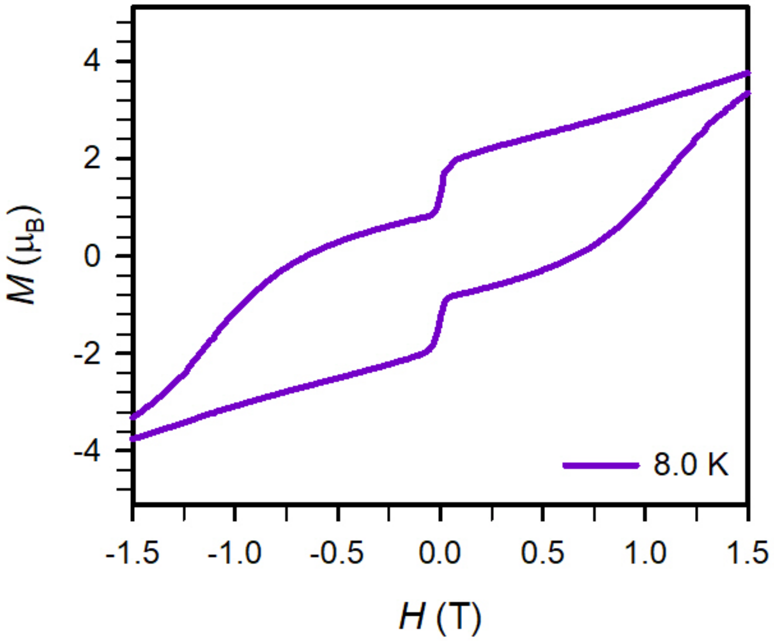

Figure S67. Plot of magnetization (*M*) vs dc magnetic field (*H*) at an average sweep rate of 100 Oe/s for **2** at 8.0 K. Solid lines are guides for the eye.

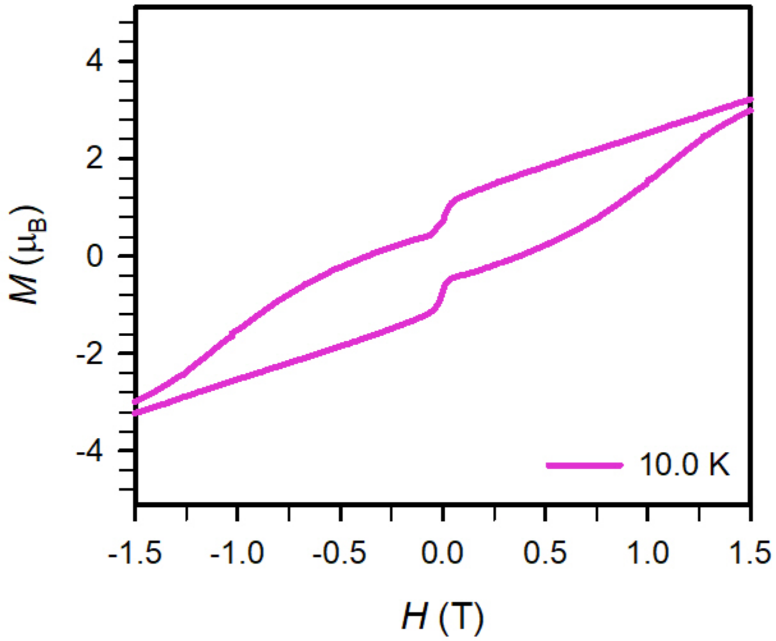

Figure S68. Plot of magnetization (*M*) vs dc magnetic field (*H*) at an average sweep rate of 100 Oe/s for **2** at 10.0 K. Solid lines are guides for the eye.

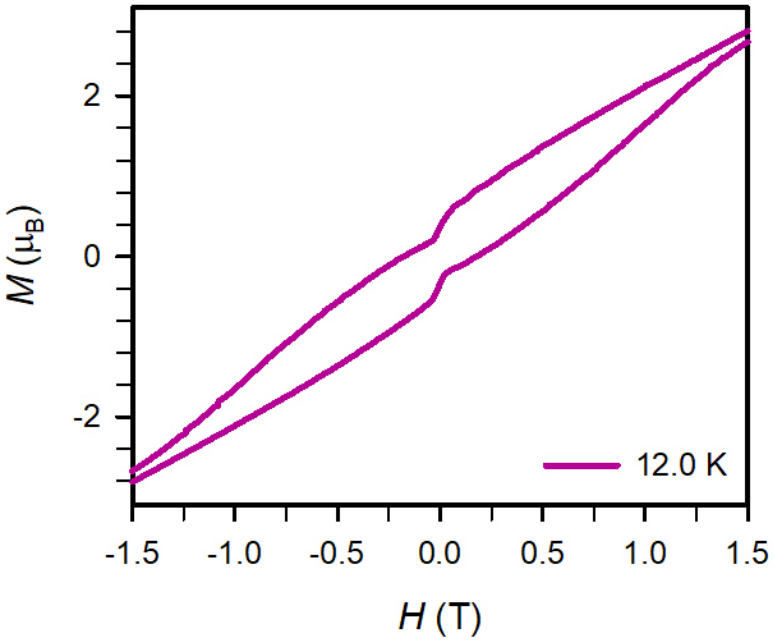

Figure S69. Plot of magnetization (*M*) vs dc magnetic field (*H*) at an average sweep rate of 100 Oe/s for **2** at 12.0 K. Solid lines are guides for the eye.

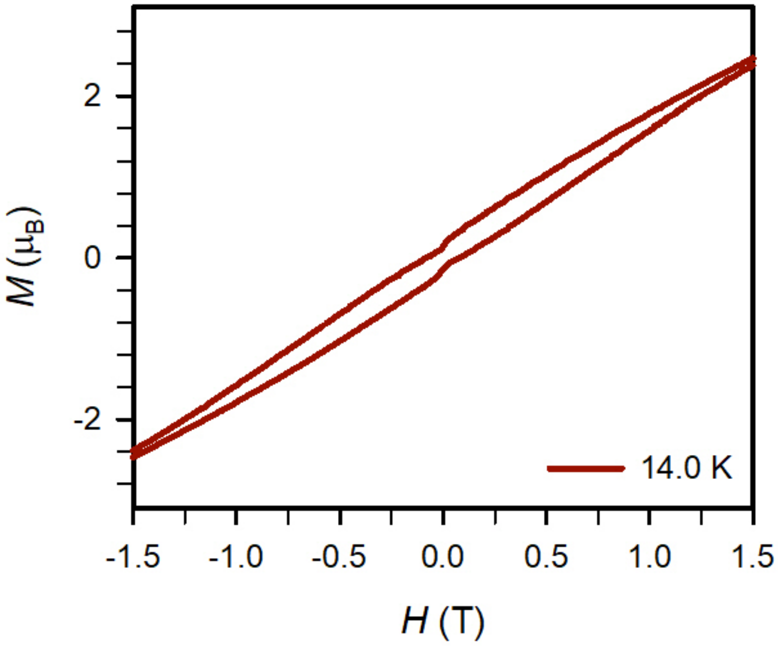

Figure S70. Plot of magnetization (*M*) vs dc magnetic field (*H*) at an average sweep rate of 100 Oe/s for **2** at 14.0 K. Solid lines are guides for the eye.

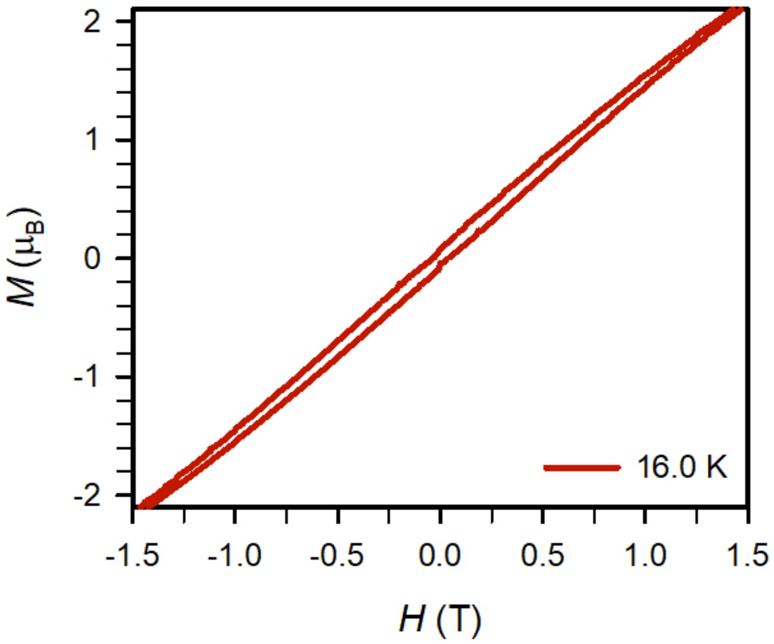

Figure S71. Plot of magnetization (M) vs dc magnetic field (H) at an average sweep rate of 100 Oe/s for **2** at 16.0 K. Solid lines are guides for the eye.

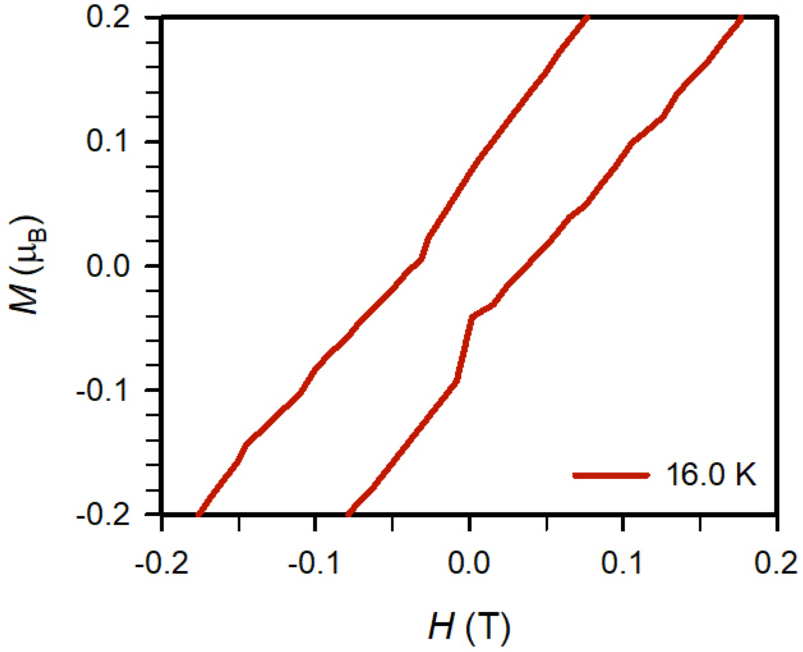

Figure S72. Magnification of variable-field magnetization data for **2** at an average sweep rate of 100 Oe/s at 16.0 K. Solid lines are guides for the eye.

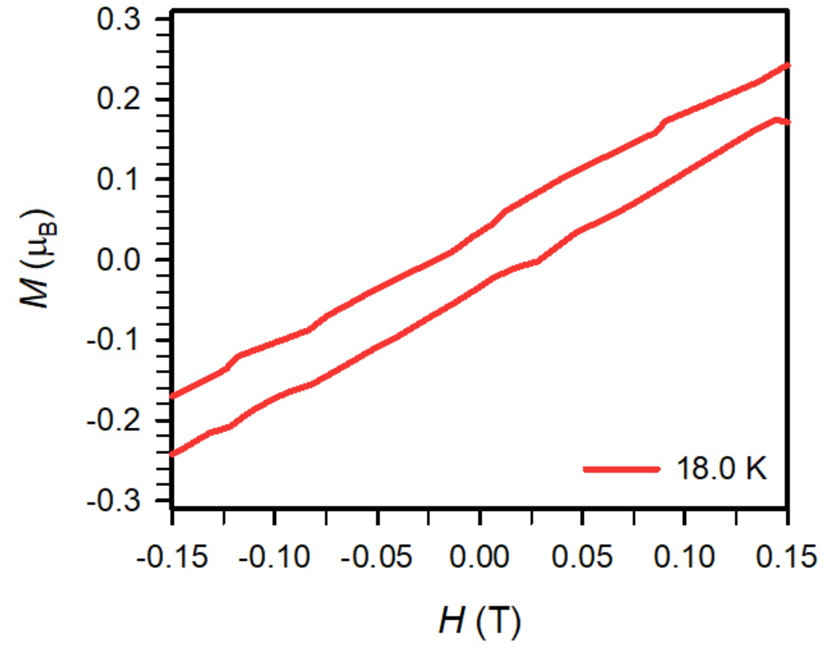

Figure S73. Plot of magnetization (*M*) vs dc magnetic field (*H*) at an average sweep rate of 100 Oe/s for **2** at 18.0 K. Solid lines are guides for the eye.

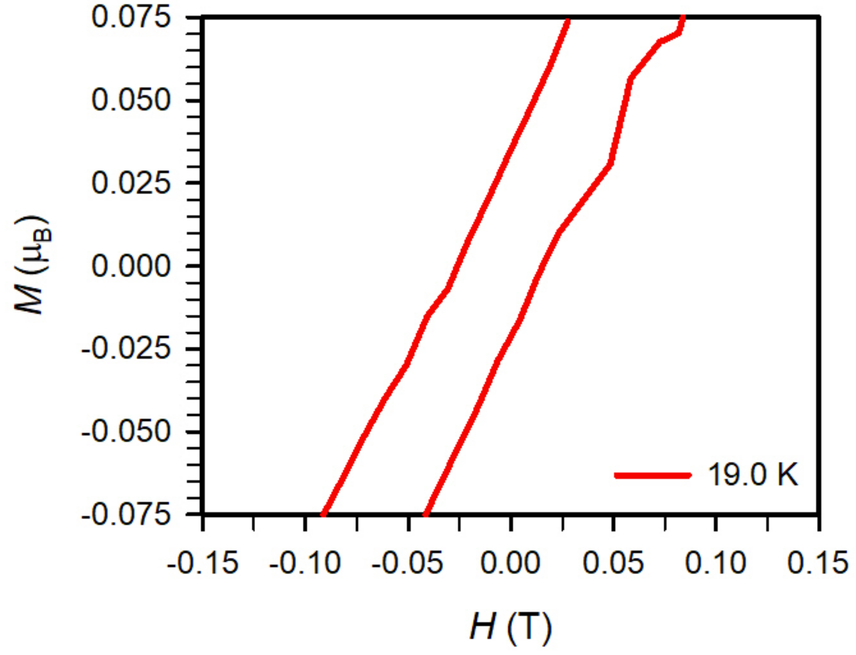

Figure S74. Plot of magnetization (*M*) vs dc magnetic field (*H*) at an average sweep rate of 100 Oe/s for **2** at 19.0 K. Solid lines are guides for the eye.

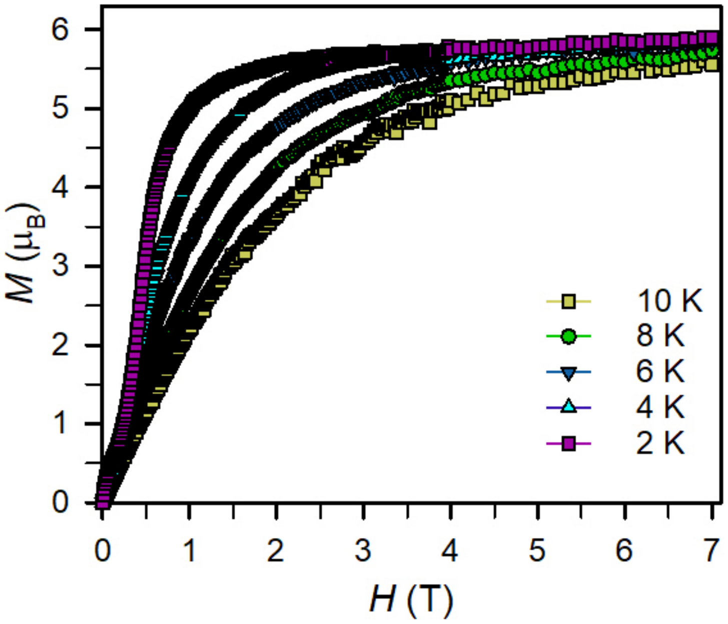

Figure S75. Variable-temperature $M(H)$ curves for **1** collected from 0 to 7 T.

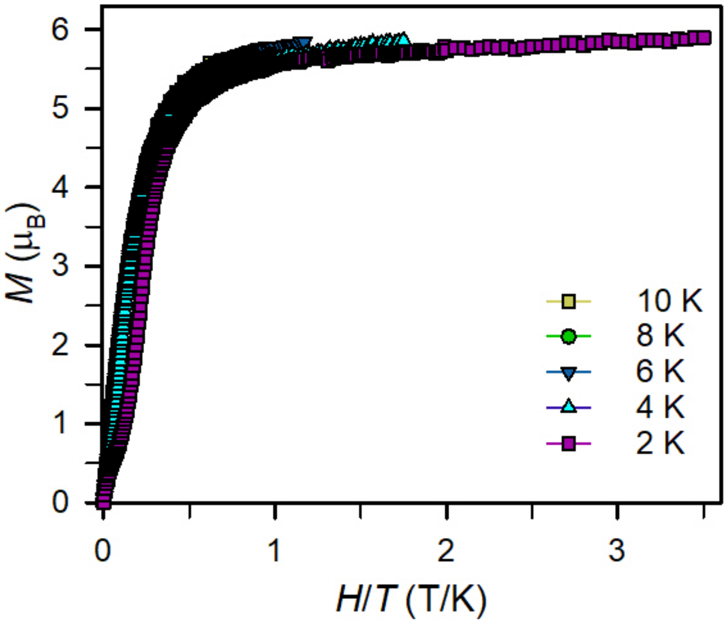

Figure S76. Reduced magnetization data for **1**, collected from 2 to 10 K, between 0 and 7 T.

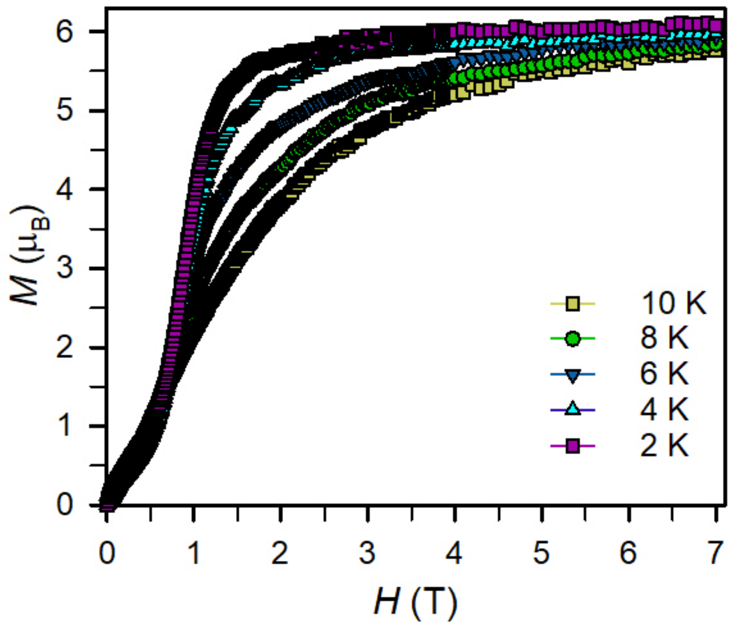

Figure S77. Variable-temperature *M*(*H*) curves for **2** collected from 0 to 7 T.

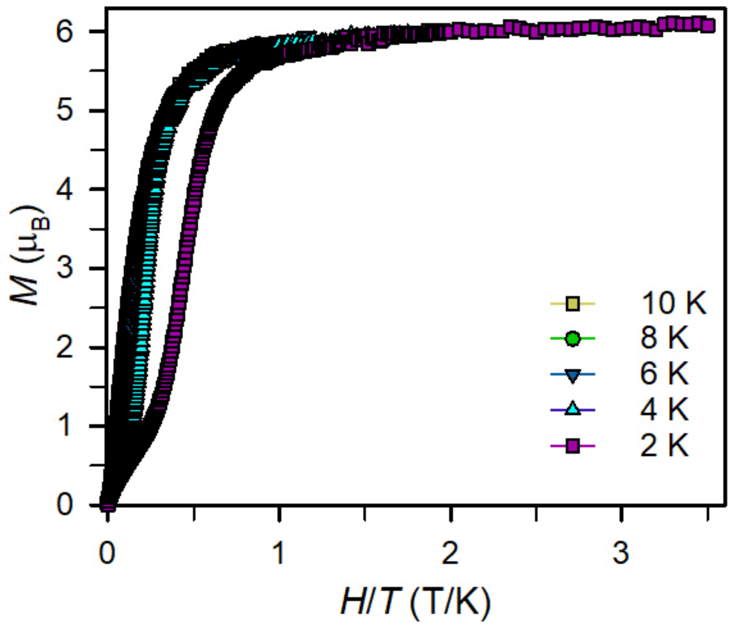

Figure S78. Reduced magnetization data for **2**, collected from 2 to 10 K, between 0 and 7 T.

8 *Ab initio* Calculations

The magnetic properties of $(NHAr^*)_2DyCl$ (**1**) and the $[(NHAr^*)_2Dy]^+$ cation in **2** were calculated via a Complete Active Space Self-Consistent Field (CASSCF)/N-Valence Electron Perturbation Theory (NEVPT2) approach[17–19] with the RI-JK approximation[19] as implemented in ORCA 5.0.4.[20,21] Scalar relativistic effects were accounted for with the second order Douglas-Kroll approach,[22,23] where the DKH-def2-SVP basis set was used for all atoms and C atoms of the peripheral Tripp groups,[24] DKH-def2-TZVP for Cl, N and C atoms[25] of the coordinating Tripp groups and SARC2-DKH-QZVP/SARC2-DKH-QZVP/JK basis sets for the Dy atoms.[26] Auxiliary basis sets for C, N and H were generated via the autoaux feature.[27]

Finer integration grids (defgrid 3) with tight convergence criteria (energy convergence tolerance: 1e−07) were used throughout with disabled frozen core approximation. The calculations were carried out on the coordinates obtained from single-crystal XRD for $(NHAr^*)_2DyCl$ (**1**) and the $[(NHAr^*)_2Dy]^+$ cation in **2** at 100 K, where all hydrogen position were initially optimized via TPSSh functional.[28,29] A nine-electrons-in-seven-4f-orbitals active space with 21 quintet, 128 triplet and 130 doublet roots considered for the state averaged (SA) CASSCF calculation. Dynamic correlation effects were introduced via strongly contracted NEVPT2 (SC-NEVPT2).[17,18,30] The construction of the fourth order reduced density matrix was simplified via the efficient implementation (D4step efficient).[31,32] The orbital energies of the SA-CASSCF were chosen as diagonal elements of the state-specific Fock operators (canonstep 0). Spin-Orbit-Coupling (SOC) effects were included within the NEVPT2 step via Quasi-Degenerate Perturbation Theory (QDPT) using the mean-field/effective potential Hamiltonian RI-SOMF(1x).[33–35] The free-particle Foldy–Wouthuysen (fpFW) transformation was carried out in the first step of the DKH protocol by including the vector potential. Picture change corrections were included on the second order, as well as finite nucleus corrections.[34] Lastly, the magnetic properties such as *g* tensors, crystal field parameters, and estimated single-ion anisotropy barrier were calculated via the SINGLE_ANISO standalone program.[36] Calculated susceptibility curves were scaled such that they align with the experimental room-temperature $\chi_M T$ values. For both complexes the scaling factor for the $\chi_M T$ vs T curves was determined as the fraction of the experimental vs. the calculated $\chi_M T$ value at 300 K,

which amounts to 1.5% (**1**) and 3.6% (**2**). Similar deviations have been observed in the literature and were corrected via small scaling factors such as 1.08 for $[Dy(Cp^{ttt})_2][B(C_6F_5)_4]$, with Cp^{ttt} = $\{C_5H_2{}^tBu_3\text{-}1,2,4\}$ and ^tBu = $C(CH_3)_3$ and fall within the accuracy of the method.[37]

Table S7. Calculated Kramers doublet (KD) energies, associated magnetic moments, *g*-tensors, and wave function composition for $(NHAr^*)_2DyCl$ (**1**). Only contributions > 10% are printed. The wave function decompositions correspond to the lowest atomic multiplet *J* = 15/2 in wave functions with definite projection of the total moment to the quantization axis.

KD	*E* (cm^{-1})	*M* (μ_B)	g_x	g_y	g_z	Wave function composition
**1**	0.0	±9.91	0.0012	0.0015	19.8237	$\lvert\pm15/2\rangle$ (100%)
**2**	315.4	±8.44	0.0327	0.0454	16.8850	$\lvert\pm13/2\rangle$ (97%)
**3**	600.9	±6.68	0.7267	1.1751	13.3661	$\lvert\pm11/2\rangle$ (85%); $\lvert\pm7/2\rangle$ (12%);
**4**	779.9	±3.42	7.8446	6.8974	4.4649	$\lvert\pm9/2\rangle$ (45%); $\lvert\pm5/2\rangle$ (28%); $\lvert\pm1/2\rangle$ (15%)
**5**	910.8	±0.49	0.9566	2.1327	10.2558	$\lvert\pm9/2\rangle$ (24%); $\lvert\pm7/2\rangle$ (20%); $\lvert\pm3/2\rangle$ (32%); $\lvert\pm1/2\rangle$ (14%)
**6**	1098.4	±0.59	0.1958	0.3454	13.9049	$\lvert\pm9/2\rangle$ (18%); $\lvert\pm7/2\rangle$ (34%); $\lvert\pm5/2\rangle$ (15%); $\lvert\pm1/2\rangle$ (28%)
**7**	1272.6	±0.85	0.0263	0.0492	17.6498	$\lvert\pm7/2\rangle$ (22 %); $\lvert\pm5/2\rangle$ (35 %); $\lvert\pm3/2\rangle$ (27 %)
**8**	1401.1	±0.99	0.0111	0.0203	19.5442	$\lvert\pm7/2\rangle$ (10 %); $\lvert\pm5/2\rangle$ (21 %); $\lvert\pm3/2\rangle$ (30 %); $\lvert\pm1/2\rangle$ (35 %)

Table S8. Calculated Kramers doublet (KD) energies associated magnetic moments, *g*-tensors and wave function composition for the $[(NHAr^*)_2Dy]^+$ cation in **2**. Only contributions > 10% are printed. The wave function decompositions correspond to the lowest atomic multiplet *J* = 15/2 in wave functions with definite projection of the total moment to the quantization axis.

KD	*E* (cm^{-1})	*M* (μ_B)	g_x	g_y	g_z	Wave function composition
**1**	0.0	9.91	0.0000	0.0000	19.8212	$\lvert\pm15/2\rangle$ (100%)
**2**	219.0	8.49	0.0006	0.0009	17.0177	$\lvert\pm13/2\rangle$ (98%)
**3**	481.7	7.00	0.0435	0.0522	14.0088	$\lvert\pm11/2\rangle$ (93%)
**4**	722.5	5.48	0.6485	0.6790	11.0302	$\lvert\pm9/2\rangle$ (84%); $\lvert\pm5/2\rangle$ (12%)
**5**	909.5	3.81	2.0616	2.7822	8.1141	$\lvert\pm7/2\rangle$ (71%); $\lvert\pm3/2\rangle$ (18%)
**6**	1021.3	2.69	0.7717	0.8889	18.4147	$\lvert\pm5/2\rangle$ (42%); $\lvert\pm3/2\rangle$ (26%); $\lvert\pm1/2\rangle$ (10%)
**7**	1045.0	1.20	1.2380	2.1356	7.1354	$\lvert\pm5/2\rangle$ (25%); $\lvert\pm1/2\rangle$ (60%)
**8**	1114.5	1.76	12.3284	8.2137	1.2694	$\lvert\pm5/2\rangle$ (20%); $\lvert\pm3/2\rangle$ (46%); $\lvert\pm1/2\rangle$ (25%)

Table S9. Crystal field parameters calculated for $(NHAr^*)_2DyCl$ (**1**) via the SINGLE_ANISO program. The Hamiltonian employed to calculate the crystal field parameters is given by:

$$\hat{H}_{CF} = \sum_{k=2,4,6} \sum_{k=-q}^{+q} \left[B_k^q \hat{O}_k^q(S) \right]$$

where $\hat{O}_k^q$ is the extended Stevens operator, B_k^q the crystal field parameter, *k* is the rank of the irreducible tensor operator (ITO) (2,4,6), *q* is the component of the ITO (*q* = −*k*, −*k*+1,…0,1,…*k*).

**k**	**q**	**Weight (%)**	B_q^k	**k**	**q**	**Weight (%)**	B_q^k
	−2	10	2.45E+00		−6	<1	-7.78E-05
	−1	<1	-1.55E-03		−5	<1	-2.79E-04
**2**	0	34	-7.03E+00		−4	2	1.31E-04
	1	<1	-1.83E-01		−3	2	2.50E-04
	2	20	5.09E+00		−2	3	-2.15E-04
	−4	1	-6.43E-03		−1	<1	-4.64E-05
	−3	2	-2.40E-02	**6**	0	3	3.51E-05
	−2	3	9.95E-03		1	<1	1.46E-05
	−1	< 1	2.78E-03		2	2	-9.74E-05
**4**	0	5	-5.35E-03		3	<1	1.03E-04
	1	<1	9.59E-04		4	<1	-4.37E-05
	2	<1	-1.60E-03		5	<1	2.52E-04
	3	1	-1.39E-02		6	2	1.40E-04
	4	2	-7.41E-03				

Table S10. Crystal field parameters calculated for $[(NHAr^*)_2Dy]^+$ cation in **2** via the SINGLE_ANISO program.

**k**	**q**	**Weight (%)**	B_q^k	**k**	**q**	**Weight (%)**	B_q^k
	−2	1.1	-2.12E-01		−6	<1	-2.66E-05
	−1	<1	2.25E-02		−5	<1	8.60E-05
**2**	0	43.0	-6.61E+00		−4	<1	-3.66E-05
	1	<1	-3.58E-01		−3	<1	-2.66E-05
	2	8.4	1.58E+00	**6**	−2	<1	4.58E-05
	−4	<1	7.73E-04		−1	<1	-1.76E-05
	−3	<1	5.55E-03		0	3.7	3.38E-05
**4**	−2	1.1	-2.85E-03		1	4.4	2.55E-04
	−1	<1	7.63E-04		2	7.3	-3.38E-04
	0	1.7	-1.46E-03		3	1.4	1.29E-04

1	2.9	-1.08E-02	4	2.6	1.30E-04
2	7.8	2.08E-02	5	1.0	-2.42E-04
3	2.7	-2.69E-02	6	<1	6.16E-05
4	<1	-2.67E-03			

Table S11. Calculated average transition dipole moments for the eight lowest lying Kramers doublets with opposing magnetization (+*I* → *I*−1), and for excited states (*I* → *I*+1), (*I* → *I*+2), (*I* → *I*+3), (*I* → *I*+4) and (*I* → *I*+5) of $(NHAr^*)_2DyCl$ (**1**).

Through Barrier (+*I* → −*I*)			Through Excited States (+*I* → *I*+1)			Through Excited States (+*I* → *I*+2)		
**KD1**	**KD2**	**Magnitude**	**KD**	**KD+1**	**Magnitude**	**KD**	**KD+2**	**Magnitude**
+1	−1	4.55E-04	+1	+2	1.75E+00	+1	+3	9.59E-02
+2	−2	1.31E-02	+1	−2	1.47E-03	+1	−3	1.28E-02
+3	−3	3.20E-01	+2	+3	2.38E+00	+2	+4	1.81E-01
+4	−4	2.08E+00	+2	−3	2.82E-02	+2	−4	2.46E-01
+5	−5	2.04E+00	+3	+4	2.62E+00	+3	+5	4.58E-01
+6	−6	5.16E-01	+3	−4	3.40E-01	+3	−5	1.06E+00
+7	−7	6.82E-02	+4	+5	2.44E+00	+4	+6	6.41E-01
+8	−8	2.55E-02	+4	−5	1.25E+00	+4	−6	4.68E-01
			+5	+6	2.29E+00	+5	+7	3.32E-01
			+5	−6	1.88E+00	+5	−7	2.41E-01
			+6	+7	2.34E+00	+6	+8	1.06E-01
			+6	−7	2.32E-01	+6	−8	1.05E+00
			+7	+8	2.74E-02			
			+7	−8	1.67E+00			

Through Excited States (+*I* → *I*+3)			Through Excited States (+*I* → *I*+4)			Through Excited States (+*I* → *I*+5)		
**KD**	**KD+3**	**Magnitude**	**KD**	**KD+4**	**Magnitude**	**KD**	**KD+5**	**Magnitude**
+1	+4	1.58E-01	+1	+5	3.67E-02	+1	+6	2.59E-02
+1	−4	1.70E-02	+1	−5	8.38E-02	+1	−6	5.34E-02
+2	+5	3.38E-01	+2	+6	1.56E-01	+2	+7	1.99E-02
+2	−5	9.59E-02	+2	−6	1.59E-01	+2	−7	9.25E-02
+3	+6	4.37E-01	+3	+7	1.10E-01	+3	+8	1.31E-01
+3	−6	3.98E-01	+3	−7	2.05E-01	+3	−8	6.10E-02
+4	+7	2.74E-01	+4	+8	1.50E-01			
+4	−7	3.08E-01	+4	−8	7.79E-02			

+5	+8	2.33E-01						
+5	−8	2.75E-01						

Table S12. Calculated average transition dipole moments for the eight lowest lying Kramers doublets with opposing magnetization (+*I* → *I*−1), and for excited states (*I* → *I*+1), (*I* → *I*+2), (*I* → *I*+3), (*I* → *I*+4) and (*I* → *I*+5) of the $[(NHAr^*)_2Dy]^+$ cation in **2**.

Through Barrier (+*I* → −*I*)			Through Excited States (+*I* → *I*+1)			Through Excited States (+*I* → *I*+2)		
**KD1**	**KD2**	**Magnitude**	**KD**	**KD+1**	**Magnitude**	**KD**	**KD+2**	**Magnitude**
+1	−1	8.16E-07	+1	+2	1.72E+00	+1	+3	1.53E-01
+2	−2	2.50E-04	+1	−2	5.29E-05	+1	−3	6.51E-04
+3	−3	1.60E-02	+2	+3	2.40E+00	+2	+4	1.41E-01
+4	−4	2.22E-01	+2	−3	1.38E-03	+2	−4	1.17E-02
+5	−5	8.37E-01	+3	+4	2.80E+00	+3	+5	3.82E-01
+6	−6	6.38E-01	+3	−4	2.85E-02	+3	−5	9.92E-02
+7	−7	1.27E+00	+4	+5	3.03E+00	+4	+6	5.95E-01
+8	−8	2.11E+00	+4	−5	1.54E-01	+4	−6	1.91E-01
			+5	+6	1.72E+00	+5	+7	2.98E+00
			+5	−6	6.42E-01	+5	−7	4.20E-01
			+6	+7	1.61E+00	+6	+8	4.45E-01
			+6	−7	5.08E-01	+6	−8	5.01E-01
			+7	+8	2.96E+00			
			+7	−8	2.12E+00			
**Through Excited States (+*I* → *I*+3)**			**Through Excited States (+*I* → *I*+4)**			**Through Excited States (+*I* → *I*+5)**		
**KD**	**KD+3**	**Magnitude**	**KD**	**KD+4**	**Magnitude**	**KD**	**KD+5**	**Magnitude**
+1	+4	1.82E-01	+1	+5	5.71E-02	+1	+6	6.39E-02
+1	−4	1.66E-03	+1	−5	9.39E-03	+1	−6	3.42E-03
+2	+5	1.65E-01	+2	+6	1.26E-01	+2	+7	2.94E-02
+2	−5	1.11E-02	+2	−6	1.51E-02	+2	−7	5.53E-03
+3	+6	2.88E-01	+3	+7	1.28E-01	+3	+8	3.84E-02
+3	−6	5.27E-02	+3	−7	7.41E-02	+3	−8	5.73E-02
+4	+7	5.60E-01	+4	+8	1.98E-01			
+4	−7	2.32E-01	+4	−8	8.31E-02			
+5	+8	6.76E-01						
+5	−8	2.73E-01						

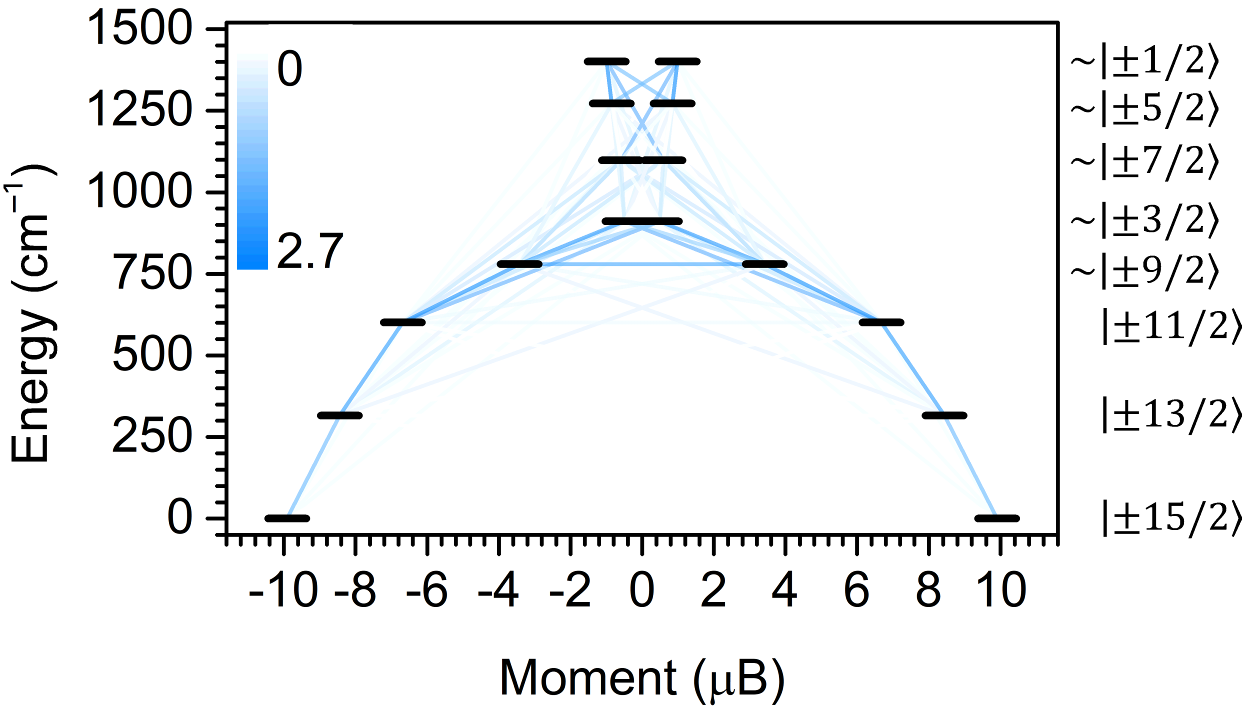

Figure S79. Calculated relaxation barrier for $(NHAr^*)_2DyCl$ (**1**). Solid lines represent possible relaxation processes as indicated by calculated transition magnetic dipole moments, where dark blue coloration represents most probable transitions and faded blue indicates vanishing probabilities. The numbers on the right are given for the primary M_J state comprising the wave function for each state.

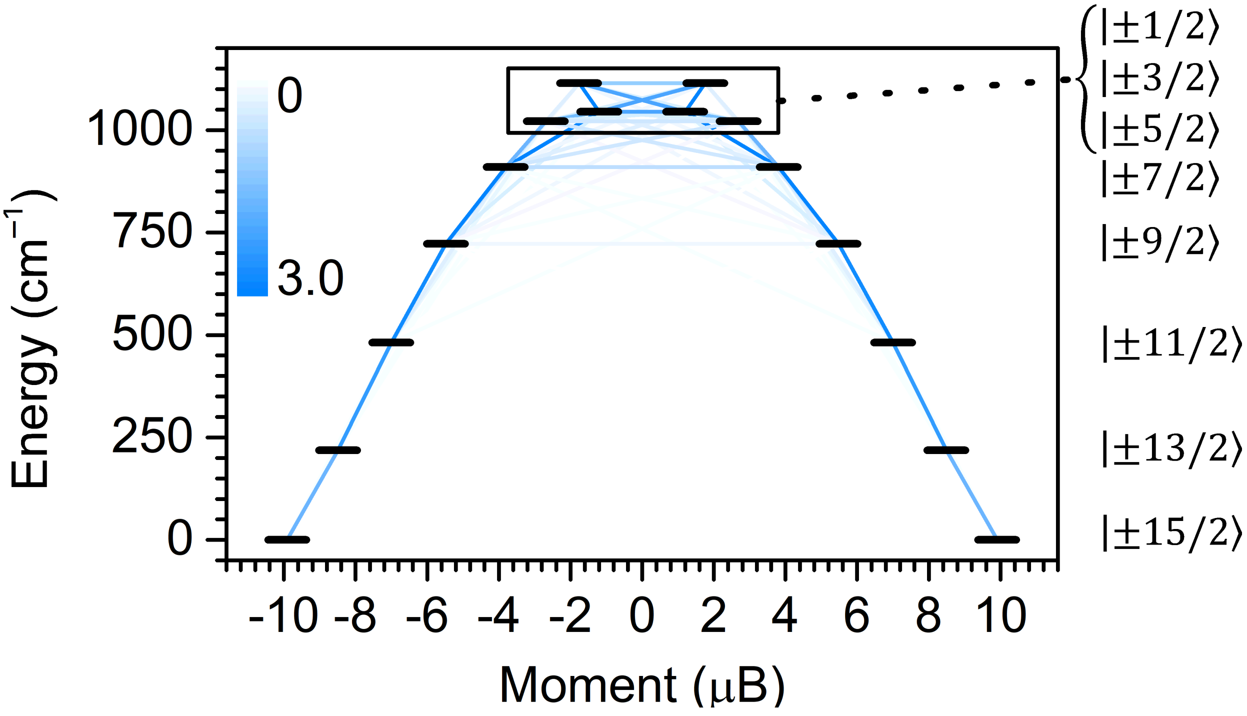

Figure S80. Calculated relaxation barrier for the $[(NHAr^*)_2Dy]^+$ cation in **2**. Solid lines represent possible relaxation processes as indicated by calculated transition magnetic dipole moments, where dark blue coloration represents most probable transitions and faded blue indicates vanishing probabilities. The numbers on the right are given for the primary M_J state comprising the wave function for each state. The three highest lying KDs are superpositions of the $|\pm 1/2\rangle$, $|\pm 3/2\rangle$ and $|\pm 5/2\rangle$ states with varying weights and therefore not further specified.

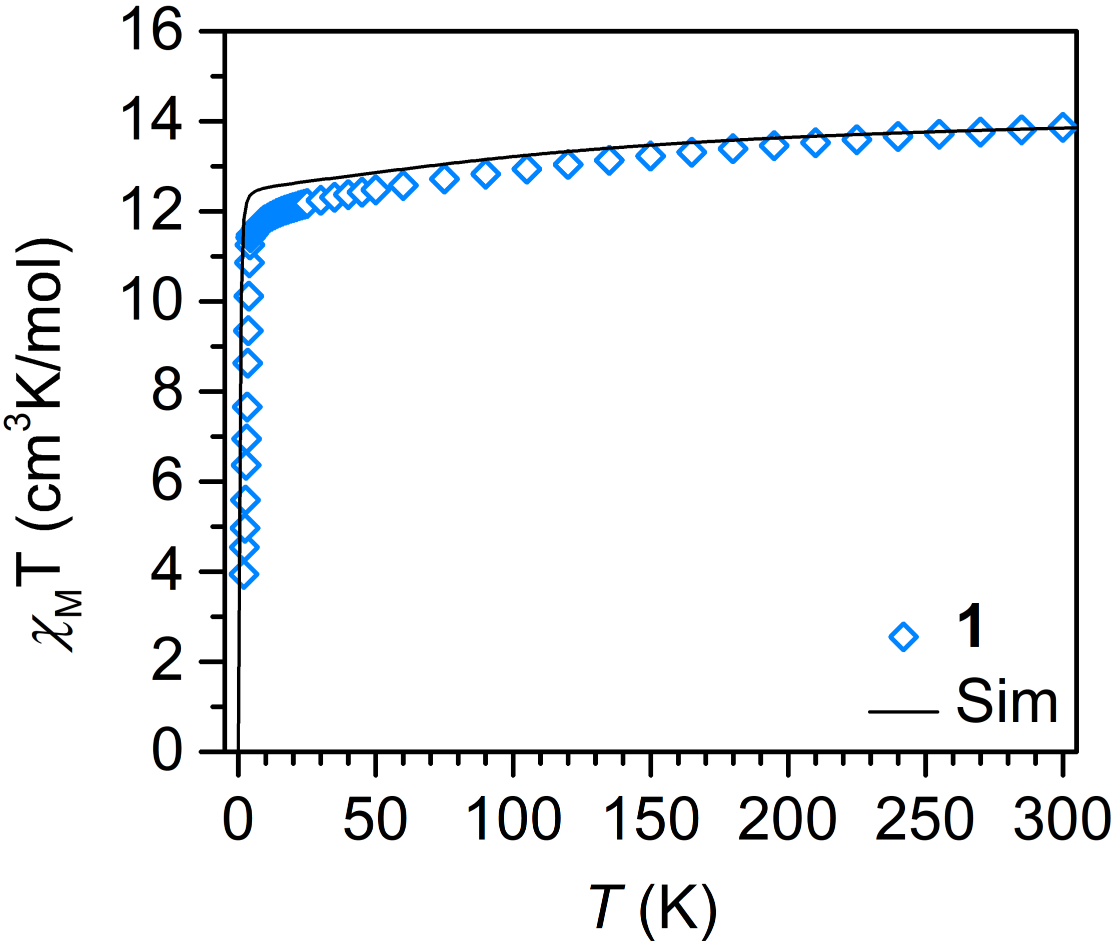

Figure S81. Variable-temperature dc magnetic susceptibility data of $(NHAr^*)_2DyCl$ (**1**), collected under a 0.1 T applied dc field (blue squares). Solid black line represents the calculated values for the $(NHAr^*)_2DyCl$ (**1**).

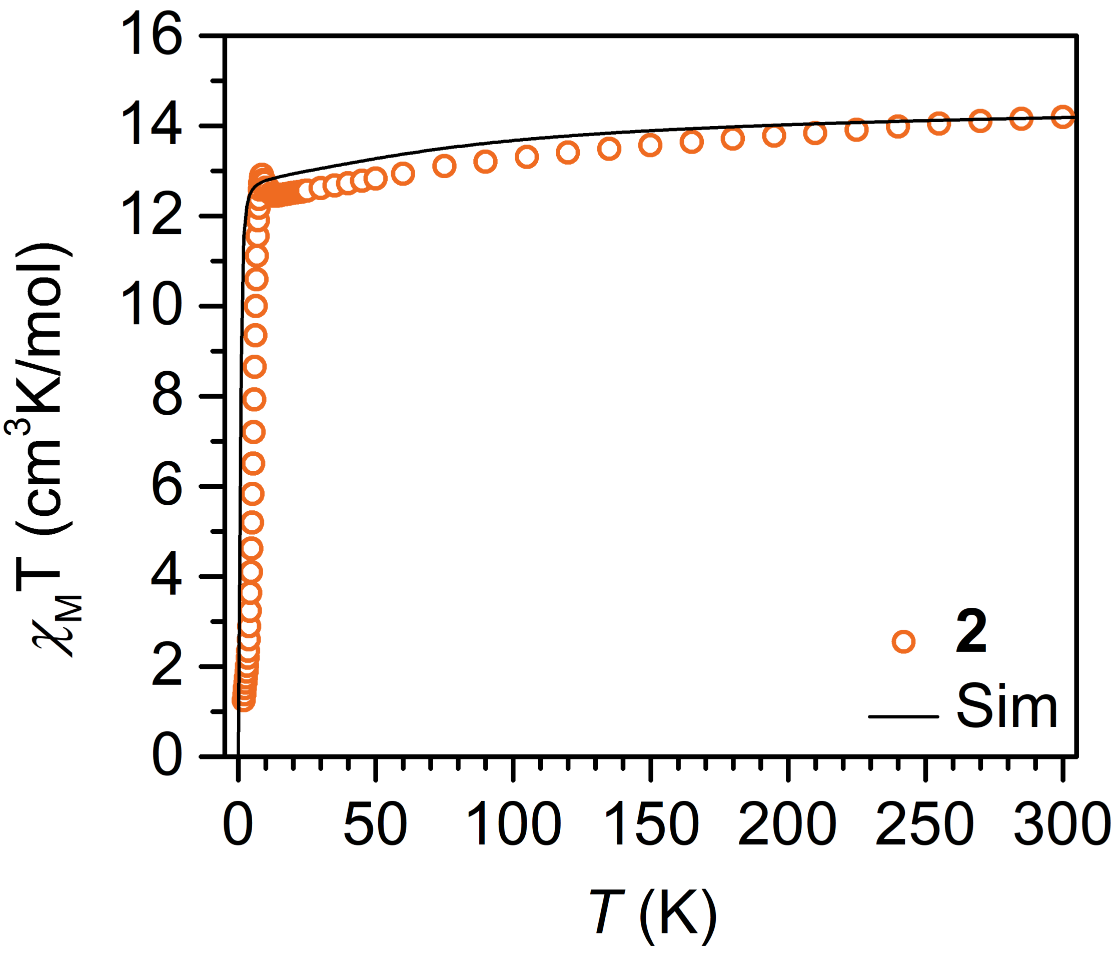

Figure S82. Variable-temperature dc magnetic susceptibility data of $[(NHAr^*)_2Dy][BArF_{24}]$ (**2**), collected under a 0.1 T applied dc field (orange circles). Solid black line represents the calculated values for the $[(NHAr^*)_2Dy]^+$ cation in **2**.

9 TDDFT Calculations

UV-vis transitions of $(NHAr^*)_2DyCl$ (**1**) and of the $[(NHAr^*)_2Dy]^+$ cation in **2** were calculated using the time-dependent density functional theory approach as implemented in the ORCA 5.0.4 program.[20,21] TDDFT calculations were carried out for 100 roots using the PBE0 functional[38–40] with the RIJCOSX acceleration.[41,42] Auxiliary basis sets were constructed using the autoaux feature.[27] The def2-TZVP basis set[43] was used for C, N and H atoms of the first coordination sphere, while the def2-SVP basis set[43] was used for the peripheral C and H atoms. For the Dy^{III} the 4*f*-in-core potential Stuttgart-Cologne

pseudopotential ECP55MWB[44,45] and associated ECP55MWB-II basis set[44,46,47] were used. Tight convergence criteria and a finer integration grid (defgrid3) were used.

Table S13. TDDFT calculated transitions for $(NHAr^*)_2DyCl$ (**1**). Energy levels are shown as obtained from TDDFT calculations without further level shifts. HOMO is 286a, LUMO is 287. Cut-off value for printing of canonical donor and acceptor orbitals: 0.1.

$\tilde{\nu}$ (cm^{-1})	λ (nm)	Fosc	Donor MO	Acceptor MO	Weight
24118.4	395.8	0.018	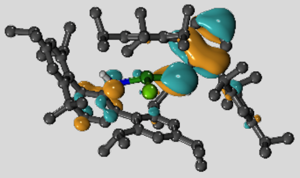 285a	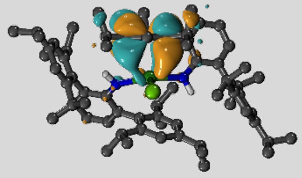 287a	0.63
			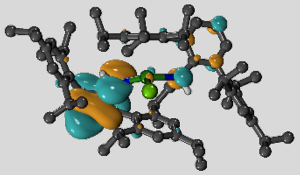 286a	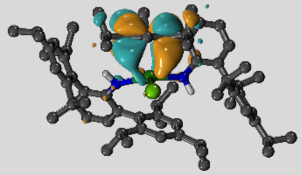 287a	0.32
32941.0	303.6	0.099	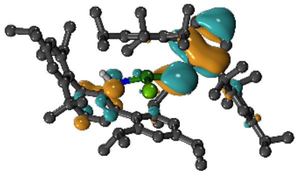 285a	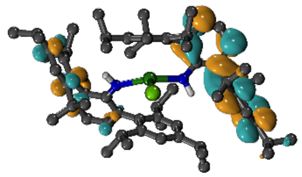 290a	0.56
			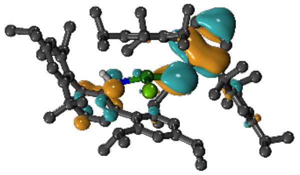 285a	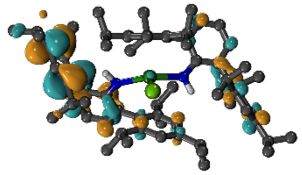 291a	0.12
			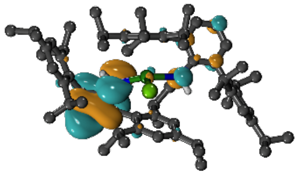 286a	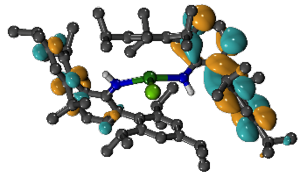 290a	0.16

39526.0	253.0	0.122	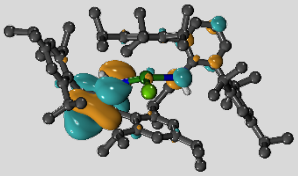 286a	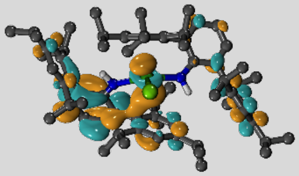 297a	0.21
			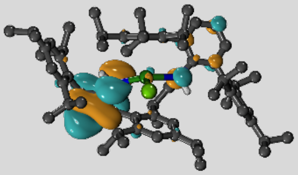 286a	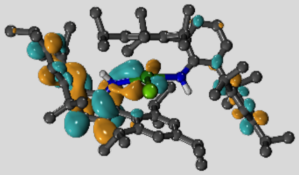 298a	0.33

Table S14. TDDFT calculated transitions for $[(NHAr^*)_2Dy][BArF_{24}]$ (**2**). Energy levels are shown as obtained from TDDFT calculations without further level shifts. HOMO is 277a, LUMO is 279. Cut-off value for printing of canonical donor and acceptor orbitals: 0.1.

$\tilde{\nu}$ (cm^{-1})	λ (nm)	Fosc	Donor MO	Acceptor MO	Weight
20822.4	480.3	0.084	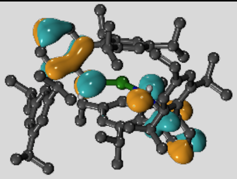 277a	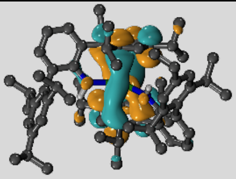 278a	0.95
26908.1	371.6	0.051	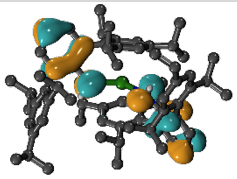 276a	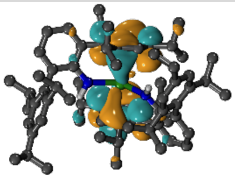 280a	0.96
35982.0	277.9	0.095	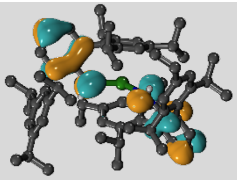 277a	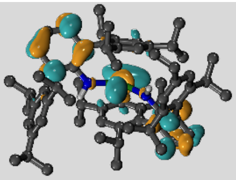 282a	0.66

			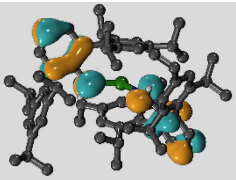<br>277a	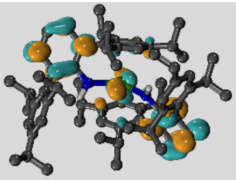<br>284a	0.10
36845.2	271.4	0.107	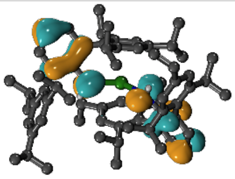<br>276a	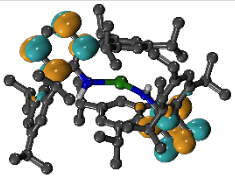<br>283a	0.15
			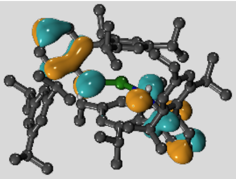<br>277a	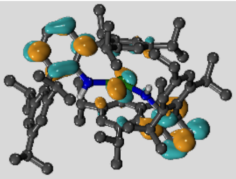<br>284a	0.56

10 Further Information

Author Contributions

SD wrote the manuscript with the input from all authors. FB conducted SQUID measurements and CASSCF calculations. FB and SD performed all magnetic characterization and interpretation. FB and SD designed and generated the Table of Contents Graphic. RJ synthesized the complexes and conducted SCXRD and spectroscopy measurements. ALO and SD led the project and provided resources and support.

11 References

(1) Clegg, W.; Conway, B.; Kennedy, A. R.; Klett, J.; Mulvey, R. E.; Russo, L. Synthesis and Structures of [(Trimethylsilyl)methyl]sodium and -potassium with Bi- and Tridentate N-Donor Ligands. *Eur. J. Inorg. Chem.* **2011**, *2011* (5), 601–760. https://doi.org/10.1002/ejic.201000983

(2) Górski, K.; Mech-Piskorz, J.; Leśniewska, B.; Pietraszkiewicz, O.; Pietraszkiewicz, M. Synthesis and Reactivity of 5-Heterotruxenes Containing Sulfur or Nitrogen as the Heteroatom. *J. Org. Chem.* **2019**, *84* (18), 11553–11561. https://doi.org/10.1021/acs.joc.9b01397

(3) Gavenonis, J.; Tilley, T. D. Synthesis and Reactivity of Alkyl, Hydride, and Silyl Derivatives of the (Terphenyl)imido Fragments Cp*(Ar^{Mes}N)Ta (Cp* = η^5-C_5Me_5; Ar^{Mes} = 2,6-(2,4,6-$Me_3C_6H_2)_2C_6H_3$) and Cp*(Ar^{Trip}N)Ta (Ar^{Trip} = 2,6-(2,4,6-$^iPr_3C_6H_2)_2C_6H_3$). *Organometallics* **2004**, *23* (1), 31–43. https://doi.org/10.1021/om030567z

(4) Dolomanov, O. V.; Bourhis, L. J.; Gildea, R. J.; Howard, J. A. K.; Puschmann, H. OLEX2: A Complete Structure Solution, Refinement and Analysis Program. *J. Appl. Crystallogr.* **2009**, *42* (2), 339–341. https://doi.org/10.1107/S0021889808042726

(5) Sheldrick, G. M. SHELXT - Integrated Space-Group and Crystal-Structure Determination. *Acta Cryst.* **2015**, *A71* (1), 3–8. https://doi.org/10.1107/S2053273314026370

(6) Bain, G. A.; Berry, J. F. Diamagnetic Corrections and Pascal's Constants. *J. Chem. Edu.* **2008**, *85* (4), 532–536. https://doi.org/10.1021/ed085p532

(7) Reta, D.; Chilton, N. F. Uncertainty Estimates for Magnetic Relaxation Times and Magnetic Relaxation Parameters. *Phys. Chem. Chem. Phys.* **2019**, *21* (42), 23567–23575. https://doi.org/10.1039/c9cp04301b

(8) Blackmore, W. J. A.; Gransbury, G. K.; Evans, P.; Mills, D. P.; Chilton, N. F. Characterisation of Magnetic Relaxation on Extremely Long Timescales. *Phys. Chem. Chem. Phys.* **2023**, *25* (25), 16735–16744. https://doi.org/10.1039/d3cp01278f

(9) Liu, S. S.; Yan, B.; Meng, Z. S.; Gao, C.; Wang, B. W.; Gao, S. Two Half-Sandwich Organometallic Single-Ion Magnets with Toluene Coordinated to the Dy(III) Ion: The [(C_7H_8)Dy($AlCl_4)_3$] and [(C_7H_8)Dy($AlBr_4)_3$] Complexes. *Inorg. Chem. Commun.* **2017**, *86* (December 2017), 312–314. https://doi.org/10.1016/j.inoche.2017.05.006

(10) Liu, S. S.; Ziller, J. W.; Zhang, Y. Q.; Wang, B. W.; Evans, W. J.; Gao, S. A Half-Sandwich Organometallic Single-Ion Magnet with Hexamethylbenzene Coordinated to the Dy(III) Ion. *Chem. Commun.* **2014**, *50* (77), 11418–11420. https://doi.org/10.1039/c4cc04262j

(11) Sun, R.; Wang, C.; Wang, B. W.; Wang, Z. M.; Chen, Y. F.; Tamm, M.; Gao, S. Low-Coordinate Bis(Imidazolin-2-Iminato) Dysprosium(III) Single-Molecule Magnets. *Inorg. Chem. Front.* **2022**, *10* (2), 485–492. https://doi.org/10.1039/d2qi02180c

(12) Harriman, K. L. M.; Murillo, J.; Suturina, E. A.; Fortier, S.; Murugesu, M. Relaxation Dynamics in See-Saw Shaped Dy(III) Single-Molecule Magnets. *Inorg. Chem. Front.* **2020**, *7* (24), 4805–4812. https://doi.org/10.1039/d0qi01007c

(13) Delano, F.; Demir, S. Guanidinate Rare-Earth Tetraphenylborate Complexes and Their Prospects in Single-Molecule Magnetism. *Cryst. Growth Des.* **2023**, *23* (5), 3134–3143. https://doi.org/10.1021/acs.cgd.2c01258

(14) Yang, K.; Sun, R.; Zhao, J.; Deng, C.; Wang, B.; Gao, S.; Huang, W. A Combined Synthetic, Magnetic, and Theoretical Study on Enhancing Ligand-Field Axiality for Dy(III) Single-Molecule Magnets Supported by Ferrocene Diamide Ligands. *Inorg. Chem.* **2023**, *62* (25), 9892–9903. https://doi.org/10.1021/acs.inorgchem.3c00896
(15) Harriman, K. L. M.; Brosmer, J. L.; Ungur, L.; Diaconescu, P. L.; Murugesu, M. Pursuit of Record Breaking Energy Barriers: A Study of Magnetic Axiality in Diamide Ligated Dy^{III} Single-Molecule Magnets. *J. Am. Chem. Soc.* **2017**, *139* (4), 1420–1423. https://doi.org/10.1021/jacs.6b12374
(16) Emerson-King, J.; Gransbury, G. K.; Whitehead, G. F. S.; Vitorica-Yrezabal, I. J.; Rouzières, M.; Clérac, R.; Chilton, N. F.; Mills, D. P. Isolation of a Bent Dysprosium Bis(Amide) Single-Molecule Magnet. *J. Am. Chem. Soc.* **2024**, *146* (5), 3331–3342. https://doi.org/10.1021/jacs.3c12427
(17) Angeli, C.; Cimiraglia, R.; Evangelisti, S.; Leininger, T.; Malrieu, J. P. Introduction of N-Electron Valence States for Multireference Perturbation Theory. *J. Chem. Phys.* **2001**, *114* (23), 10252. https://doi.org/10.1063/1.1361246
(18) Angeli, C.; Cimiraglia, R.; Malrieu, J. P. N-Electron Valence State Perturbation Theory: A Fast Implementation of the Strongly Contracted Variant. *Chem. Phys. Lett.* **2001**, *350* (3–4), 297–305. https://doi.org/10.1016/S0009-2614(01)01303-3
(19) Kollmar, C.; Sivalingam, K.; Helmich-Paris, B.; Angeli, C.; Neese, F. A Perturbation-Based Super-CI Approach for the Orbital Optimization of a CASSCF Wave Function. *J. Comput. Chem.* **2019**, *40* (14), 1463–1470. https://doi.org/10.1002/jcc.25801
(20) Neese, F. The ORCA Program System. *Wiley Interdiscip. Rev. Comput. Mol. Sci.* **2012**, *2* (1), 73–78. https://doi.org/10.1002/wcms.81
(21) Neese, F. Software Update: The ORCA Program System—Version 5.0. *Wiley Interdiscip. Rev. Comput. Mol. Sci.* **2022**, *12* (5), 1–15. https://doi.org/10.1002/wcms.1606
(22) Douglas, M.; Kroll, N. M. Quantum Electrodynamical Corrections to the Fine Structure of Helium. *Ann. Phys.* **1974**, *82* (1), 89–155. https://doi.org/10.1016/0003-4916(74)90333-9
(23) Hess, B. A. Applicability of the No-Pair Equation with Free-Particle Projection Operators to Atomic and Molecular Structure Calculations. *Phys. Rev. A* **1985**, *32* (2), 756–763. https://doi.org/10.1103/PhysRevA.32.756
(24) Weigend, F.; Ahlrichs, R. Balanced Basis Sets of Split Valence, Triple Zeta Valence and Quadruple Zeta Valence Quality for H to Rn: Design and Assessment of Accuracy. *Phys. Chem. Chem. Phys.* **2005**, *7* (18), 3297–3305. https://doi.org/10.1039/b508541a
(25) Rolfes, J. D.; Neese, F.; Pantazis, D. A. All-Electron Scalar Relativistic Basis Sets for the Elements Rb–Xe. *J. Comput. Chem.* **2020**, *41* (20), 1842–1849. https://doi.org/10.1002/jcc.26355
(26) Aravena, D.; Neese, F.; Pantazis, D. A. Improved Segmented All-Electron Relativistically Contracted Basis Sets for the Lanthanides. *J. Chem. Theory Comput.* **2016**, *12* (3), 1148–1156. https://doi.org/10.1021/acs.jctc.5b01048
(27) Stoychev, G. L.; Auer, A. A.; Neese, F. Automatic Generation of Auxiliary Basis Sets. *J. Chem. Theory Comput.* **2017**, *13* (2), 554–562. https://doi.org/10.1021/acs.jctc.6b01041

(28) Staroverov, V. N.; Scuseria, G. E.; Tao, J.; Perdew, J. P. Comparative Assessment of a New Nonempirical Density Functional: Molecules and Hydrogen-Bonded Complexes. *J. Chem. Phys.* **2003**, *119* (23), 12129–12137. https://doi.org/10.1063/1.1626543
(29) Staroverov, V. N.; Scuseria, G. E.; Tao, J.; Perdew, J. P. Erratum: Comparative Assessment of a New Nonempirical Density Functional: Molecules and Hydrogen-Bonded Complexes(Journal of Chemical Physics (**2003**) *119* (12129)). *J. Chem. Phys.* **2004**, *121* (22), 11507. https://pubs.aip.org/aip/jcp/article/121/22/11507/534770/Erratum-Comparative-assessment-of-a-new
(30) Angeli, C.; Cimiraglia, R.; Malrieu, J. P. N-Electron Valence State Perturbation Theory: A Spinless Formulation and an Efficient Implementation of the Strongly Contracted and of the Partially Contracted Variants. *J. Chem. Phys.* **2002**, *117* (20), 9138–9153. https://doi.org/10.1063/1.1515317
(31) Guo, Y.; Sivalingam, K.; Kollmar, C.; Neese, F. Approximations of Density Matrices in N-Electron Valence State Second-Order Perturbation Theory (NEVPT2). II. The Full Rank NEVPT2 (FR-NEVPT2) Formulation. *J. Chem. Phys.* **2021**, *154* (21), 214113. https://doi.org/10.1063/5.0051218
(32) Kollmar, C.; Sivalingam, K.; Guo, Y.; Neese, F. An Efficient Implementation of the NEVPT2 and CASPT2 Methods Avoiding Higher-Order Density Matrices. *J. Chem. Phys.* **2021**, *155* (23), 234104. https://doi.org/10.1063/5.0072129
(33) Neese, F. Efficient and Accurate Approximations to the Molecular Spin-Orbit Coupling Operator and Their Use in Molecular *g*-Tensor Calculations. *J. Chem. Phys.* **2005**, *122* (3), 034107. https://doi.org/10.1063/1.1829047
(34) Atanasov, M.; Aravena, D.; Suturina, E.; Bill, E.; Maganas, D.; Neese, F. First Principles Approach to the Electronic Structure, Magnetic Anisotropy and Spin Relaxation in Mononuclear 3d-Transition Metal Single Molecule Magnets. *Coord. Chem. Rev.* **2015**, *289–290* (1), 177–214. https://doi.org/10.1016/j.ccr.2014.10.015
(35) Neese, F.; Petrenko, T.; Ganyushin, D.; Olbrich, G. Advanced Aspects of Ab Initio Theoretical Optical Spectroscopy of Transition Metal Complexes: Multiplets, Spin-Orbit Coupling and Resonance Raman Intensities. *Coord. Chem. Rev.* **2007**, *251* (3–4), 288–327. https://doi.org/10.1016/j.ccr.2006.05.019
(36) Chibotaru, L. F.; Ungur, L. Ab Initio Calculation of Anisotropic Magnetic Properties of Complexes. I. Unique Definition of Pseudospin Hamiltonians and Their Derivation. *J. Chem. Phys.* **2012**, *137* (6), 064112. https://doi.org/10.1063/1.4739763
(37) Goodwin, C. A. P.; Ortu, F.; Reta, D.; Chilton, N. F.; Mills, D. P. Molecular Magnetic Hysteresis at 60 Kelvin in Dysprosocenium. *Nature* **2017**, *548* (7668), 439–442. https://doi.org/10.1038/nature23447
(38) Perdew, J. P.; Burke, K.; Ernzerhof, M. Generalized Gradient Approximation Made Simple. *Phys. Rev. Lett.* **1996**, *77* (18), 3865–3868. https://doi.org/10.1103/PhysRevLett.77.3865
(39) Perdew, J. P.; Burke, K.; Ernzerhof, M. ERRATA Generalized Gradient Approximation Made Simple. *Phys. Rev. Lett.* **1997**, *78* (7), 1396–1396. https://doi.org/10.1103/PhysRevLett.78.1396

(40) Adamo, C.; Barone, V. Toward Reliable Density Functional Methods without Adjustable Parameters: The PBE0 Model. *J. Chem. Phys.* **1999**, *110* (13), 6158–6170. https://doi.org/10.1063/1.478522
(41) Neese, F.; Wennmohs, F.; Hansen, A.; Becker, U. Efficient, Approximate and Parallel Hartree–Fock and Hybrid DFT Calculations. A 'Chain-of-Spheres' Algorithm for the Hartree–Fock Exchange (RIJCOSX). *Chem. Phys.* **2009**, *356* (1-3), 98. https://doi.org/10.1016/j.chemphys.2008.10.036
(42) Izsák, R.; Neese, F. An Overlap Fitted Chain of Spheres Exchange Method. *J. Chem. Phys.* **2011**, *135* (14), 144105. https://doi.org/10.1063/1.3646921
(43) Weigend, F.; Ahlrichs, R. Balanced Basis Sets of Split Valence, Triple Zeta Valence and Quadruple Zeta Valence Quality for H to Rn: Design an Assessment of Accuracy. *Phys. Chem. Chem. Phys.* **2005**, *7* (18), 3297. https://doi.org/10.1039/B508541A
(44) Dolg, M.; Stoll, H.; Savin, A.; Preuss, H. Energy-Adjusted Pseudopotentials for the Rare Earth Elements. *Theor. Chim. Acta* **1989**, *75*, 173–194. https://doi.org/10.1007/BF00528565
(45) Dolg, M.; Stoll, H.; Preuss, H. A Combination of Quasirelativistic Pseudopotential and Ligand Field Calculations for Lanthanoid Compounds. *Theor. Chim. Acta* **1993**, *85*, 441–450. https://doi.org/10.1007/BF01112983
(46) Yang, J.; Dolg, M. Valence Basis Sets for Lanthanide 4f-in-Core Pseudopotentials Adapted for Crystal Orbital *Ab Initio* Calculations. *Theor. Chem. Acc.* **2005**, *113*, 212–224. https://doi.org/10.1007/s00214-005-0629-0
(47) Weigand, A.; Cao, X.; Yang, J.; Dolg, M. Quasirelativistic F-in-Core Pseudopotentials and Core-Polarization Potentials for Trivalent Actinides and Lanthanides: Molecular Test for Trifluorides. *Theor. Chem. Acc.* **2009**, *126*, 117–127. https://doi.org/10.1007/s00214-009-0584-2

12 Optimized Geometries

$(NHAr^*)_2DyCl$ (1).

Dy	2.16016491381165	1.94622904800293	5.91788436721669
Cl	4.66653116827460	1.74671400631934	5.86906599623117
N	1.40973651531609	0.12666573085889	4.81084347353788
H	1.62912888431553	-0.78653910807090	5.20544221803725
N	1.67493908571612	3.22447903667761	7.68535557539106
H	1.89378489826054	4.20628831100282	7.52498633261401
C	0.67577887725866	-0.00907206928144	3.66133607064016
C	0.97213753316807	3.11238565316212	8.86053537133230
C	0.82020361061427	2.44832560715394	3.52816001323404
C	2.17051730065455	2.77912965716826	3.15821724708755
C	2.78985279402636	3.87194853199560	3.76451342115571
H	3.82065333837221	4.08869089146447	3.51604414444086
C	2.12581507644301	4.68613339776464	4.68453749432598
C	0.79378356259737	4.38727280187456	4.98591311808910
H	0.27167029047433	5.01704347434859	5.69664231646484

C	0.12700167692910	3.27978196608570	4.43309461013300
C	1.19410243380386	0.60147410445740	8.69482859511833
C	2.59027341281642	0.35460020551162	8.82690032555017
H	-0.93186989870474	2.58200590170219	6.92296250306542
C	0.24137765569116	1.17309935369040	3.00812839951554
C	0.36137725645811	-1.25478977469750	3.02890695139041
C	0.60339910629768	1.82475254036673	9.33764395630518
C	0.60154967613031	4.26103430387268	9.63782820977268
C	2.87240069390896	2.01632841547518	2.05699015044873
C	2.76914872528931	5.91893122214523	5.30576037335881
C	-1.32007754646764	3.01646684622078	4.80832520995765
C	0.35904058111758	-0.38500423008777	8.10887388796160
C	3.10477532347715	-0.84797047452755	8.33734197919347
C	3.51539053080813	1.30756377164514	9.59634286424189
C	-1.59862360460490	3.18651878369035	6.30042443166864
C	-0.59492368466247	1.14375393077463	1.90504446400252
C	-0.46319168993122	-1.23587830656615	1.89702285467115
C	0.92998598765391	-2.56595807468116	3.47336176417784
C	-0.21030668085735	1.71534499596686	10.45777226424164
C	-0.18081432694761	4.08928468079201	10.76755984657735
C	1.10872645494121	5.61293959885138	9.22214406172673
H	2.45048438582054	1.00633530875860	2.03411858802593
C	2.53437779396209	2.69591219124665	0.72083580704357
C	4.39586869566477	1.91415473386635	2.20949742352471
H	2.36031055230330	6.02703912760643	6.32037219545322
C	2.35165477365877	7.15852040812693	4.50305335239599
C	4.28768420374363	5.83058383877837	5.40065526721331
H	-1.54175871421950	1.98151358047637	4.52032276209004
C	-2.22712367349783	3.93501769767378	3.98357312190526
C	0.94833718530867	-1.56876974208595	7.64401529771969
C	-1.15227980068557	-0.25757789784496	8.00814826136153
H	4.16456924596401	-1.03257754972008	8.43856324626232
C	2.29813876979362	-1.83503013660315	7.76785533570456
H	3.32971570763273	2.31203345283303	9.20510132792222
C	5.00595122039626	1.00307993753154	9.42792536467112
C	3.20124304458744	1.29560019436682	11.10747514552730
H	-2.62984392699566	2.88364581878085	6.51998282231839
H	-1.48971311635334	4.22667503673700	6.62207613016113
H	-0.91828637593644	2.08177380150270	1.45426342313638
C	-0.97924538621708	-0.06646168052106	1.35488306053531
H	-0.68605417202698	-2.18563712976970	1.41379289599227
C	2.32650494544610	-2.79302888971563	3.37074218643860
C	0.09392257789216	-3.61949353022738	3.89957164672326
H	-0.48369833717140	0.72093050958170	10.80948353373948
C	-0.62758849552244	2.83092294146510	11.16784430651009
H	-0.44257381772098	4.96285534247211	11.36327426646650

C	2.48176226258116	5.90704594946389	9.32932035107118
C	0.23399011003989	6.60922980436069	8.70597554497362
H	1.45353046319921	2.72560107917676	0.54246550228915
H	2.99901590226071	2.15008061584844	-0.10994501046416
H	2.91376254085961	3.72624444831430	0.70437667359313
H	4.88265831321873	2.88870873215980	2.06992342370284
H	4.79255150631255	1.24672662529863	1.43547237003036
H	4.68728314273788	1.52750456146839	3.19047144601363
H	1.26183425284076	7.27655185373124	4.47834281738254
H	2.70907187119426	7.08762920338620	3.46736421167152
H	2.77994186112338	8.06431893722404	4.94791321109031
H	4.75779390567932	5.90305748146202	4.41095336696119
H	4.62070150442525	4.89445575043187	5.86247062872213
H	4.67346761455395	6.65959038651055	6.00364875758495
H	-2.08277533321545	3.78634446350684	2.90744259330852
H	-2.02575835280042	4.99059665852386	4.20919750903983
H	-3.28171254553065	3.73760988595199	4.21406001239909
H	0.31207731919670	-2.32438778635427	7.19217742646507
H	-1.43792888020143	0.73156013277523	8.38319429506229
C	-1.65812611060974	-0.39719665484778	6.56914550613970
C	-1.81783671473143	-1.31977460735630	8.90482689836419
C	2.83535397646930	-3.18204927636685	7.32768379271273
H	5.31390262049557	0.99467618172467	8.37729949726791
H	5.27389214420905	0.03998523543374	9.88351182269892
H	5.59373852228115	1.77085710154984	9.94444650793558
H	3.37058349936693	0.29266113056074	11.52247883734502
H	2.17262904379862	1.59048485363261	11.33002612537005
H	3.87325252820780	1.98866362292724	11.63017432797639
H	-1.62845163616125	-0.10244362177079	0.48237809573181
C	2.83334229122727	-4.06074804317870	3.72740643733760
C	3.27245083583495	-1.75909441193199	2.78788471229837
C	0.65979488342712	-4.83987809569929	4.25349914949414
C	-1.42999141494315	-3.50814532643307	3.94834359532272
H	-1.25069974964211	2.72258244461439	12.05325940966101
C	2.96151634765076	7.14958189097684	8.88894633126372
C	3.46714622489846	4.93742481754154	9.96861817521776
C	0.76800932402430	7.80541114369223	8.26836017338623
C	-1.28039593232168	6.43093370765160	8.70676104487405
H	-1.44850355066938	-1.39228825610948	6.16616899004266
H	-1.18219288282887	0.32420599661378	5.90014207477466
H	-2.74491689131257	-0.24526566789740	6.53176399083211
H	-2.91057293291633	-1.21468931170259	8.88108723892024
H	-1.49050559739360	-1.23143775168425	9.94731041568684
H	-1.57077309201517	-2.33243088727392	8.56302672056145
H	2.38915818115830	-3.40945698918962	6.35022997255117
C	4.36195314823755	-3.22043503539193	7.17708731236556

C	2.40010034912763	-4.27481021879831	8.30946961697526
H	3.90574637971948	-4.22250183115872	3.63350314555747
C	2.03477583514735	-5.08739542997415	4.17282496735273
H	2.76362107353468	-0.79214920507518	2.82948048814630
C	3.52925330838010	-2.07832176667917	1.33266008524785
C	4.57078490102623	-1.61054331464447	3.56434044199443
H	0.00613245381595	-5.64071463479046	4.60102677723549
H	-1.70212999935050	-2.45442270697949	3.81583207961926
C	-2.01664111393138	-3.99225741744382	5.28388293443932
C	-2.06448335785816	-4.32379084631059	2.81382318023115
H	4.02571523460266	7.35677539278668	8.98765617241777
C	2.12536025411900	8.10430513562694	8.32342169125457
H	2.93836639772153	3.99407114405589	10.13424931832198
C	3.90231498073293	5.45491496087313	11.34003380656511
C	4.69122834126410	4.65679371381852	9.09380373763253
H	0.09528771782385	8.56072367672723	7.85824149241456
H	-1.49774463385725	5.36231727663347	8.82405848295636
C	-1.92978368196787	6.92153709334745	7.39934245824995
C	-1.88326452357736	7.18331236892179	9.89409685915168
H	4.67396236733146	-4.18679045808641	6.76559932665176
H	4.86144401297356	-3.10591760615012	8.14816053957410
H	4.73223009467329	-2.43521504256094	6.50987045284320
H	2.82246061358880	-4.09132456324074	9.30635448238155
H	2.74652888102070	-5.25990847181367	7.97081350591283
H	1.30911231564573	-4.31802662930818	8.41192885236282
C	2.56806157107231	-6.45514366594252	4.57475387775936
H	2.59291984230486	-2.12990829177139	0.76301977030364
H	4.03902667734899	-3.04544757436437	1.22066365052440
H	4.16595488487138	-1.31357340024794	0.86665100038182
H	5.14637457380998	-2.54500560508360	3.59585271429095
H	4.38740647407009	-1.28444440306396	4.59322930189900
H	5.21175032152064	-0.85633977750005	3.09346171644900
H	-1.90367525294836	-5.07734597466907	5.40371334779108
H	-3.09042685248349	-3.76980550071470	5.33033531329488
H	-1.53541108503095	-3.51358234210563	6.14359624650897
H	-3.15770423814350	-4.21920208899357	2.82426808034067
H	-1.82737486481849	-5.38990096217196	2.92620088963468
H	-1.70537309055032	-4.00979564993406	1.82725382142121
C	2.61014816332405	9.45004688533889	7.83093166605107
H	4.56889652942840	4.73420638955374	11.83154540898400
H	3.03929290678736	5.62057610171749	11.99647519156071
H	4.44456346411573	6.40653722297149	11.25397759303858
H	5.25014186436718	5.57556169429522	8.87197096564213
H	4.41559836518459	4.18583523946897	8.14436899709952
H	5.37962280316854	3.97863179164985	9.61090588506464
H	-1.45259847168178	6.48459356468226	6.51432792062672

H	-1.87044350092026	8.01293390008888	7.30260470401485
H	-2.99366792982925	6.65260907437804	7.37780159385753
H	-1.46126173535571	6.84481909679574	10.84661821403516
H	-2.97169141194672	7.04156082896960	9.93736309839277
H	-1.68619016603081	8.26025854316069	9.80922879702439
H	2.15025633076525	-6.66286119391937	5.57400738841205
C	2.03842005338531	-7.54053424983420	3.63778246929396
C	4.07252170430362	-6.54705852509350	4.68748504702556
C	4.11260068260444	9.51240448679660	7.55825112554384
H	2.09704505217109	9.63413168301105	6.87275563044750
C	2.18556826537792	10.56601282953770	8.78238162681995
H	2.35405425398015	-8.53636271858583	3.97610769343410
H	2.42138093486236	-7.39287394324000	2.61899977892577
H	0.94370417385679	-7.53579303794073	3.58352701880649
H	4.36547129951832	-7.53724411811674	5.05845241509318
H	4.48234858276046	-5.79955387870177	5.37630277109375
H	4.56173406644564	-6.40838828266885	3.71346600908251
H	4.37564888315501	10.47629359734647	7.10505956021725
H	4.43819176316308	8.71963654137471	6.87513132768262
H	4.69540024123184	9.41999725590285	8.48423289645581
H	1.10083922238623	10.56873627156147	8.94183803530285
H	2.47179901927288	11.54939078842182	8.38643886163208
H	2.66443568492394	10.44355570809164	9.76308176449895

$[(NHAr^*)_2Dy]^+$ cation in 2.

Dy	10.27897775601606	9.84804977881412	7.37638409864284
N	12.17127871528691	10.93024283438317	6.94500401385645
N	8.38604420855262	10.93025672035283	7.80713140943268
H	12.24130235130901	11.51626706257010	6.11563810568721
H	8.31624279124467	11.51610556365500	8.63661963416943
C	13.40719329901292	10.91970944332518	7.58808813931811
C	7.15012955236244	10.91973010510689	7.16404731315553
C	12.22452643524644	9.67799561959665	9.34022265676611
C	8.33278800617770	9.67800392068812	5.41191587538259
C	11.31029339753766	10.59899942809398	9.91369012640762
C	9.24702727691485	10.59900011314993	4.83844611259418
C	10.03695564143548	10.15765417397279	10.26199159886361
C	10.52036204071717	10.15764536719139	4.49014572914461
H	9.33821096788532	10.85402843568675	10.71221426525603
H	11.21922123309860	10.85414307851972	4.04029471670641
C	9.61952775203500	8.84701533886177	10.06203779612103
C	10.93778105216813	8.84700420108847	4.69010278911704
C	10.53392769902080	7.93176155846944	9.51198034274154

C	10.02337491172787	7.93175798222224	5.24015252161194
H	10.24230470025643	6.89503165884527	9.40052502803465
H	10.31511714935935	6.89510709451144	5.35203100783749
C	11.85429252934076	8.32140667775452	9.18439517679766
C	8.70301272445291	8.32141285890628	5.56774672493676
C	13.51007835623832	10.21736060946340	8.81241268357673
C	14.54026050023526	11.61895500375465	7.09987772050105
C	7.04910958168858	10.21737892485300	5.94090476506511
C	6.01893693099965	11.61898454530998	7.65342624212533
C	11.72724342189228	12.02297843380657	10.25109373268204
C	8.83194673856082	12.02298109957951	4.50220921802824
C	8.25029648684108	8.44145062585919	10.58831648887484
C	12.30887941337964	8.44142890539158	4.16500534544307
C	12.86489543906415	7.27248272377150	8.75920066394810
C	7.69427255115852	7.27249680648532	5.99411409916482
C	14.70040269801887	10.20623747229998	9.53262075481039
C	15.71669220878526	11.57144193169496	7.83899399455164
C	14.40436630165134	12.48095394665395	5.87312859809850
C	5.85813524686613	10.20626205517782	5.22006663082378
C	4.84186498210858	11.57147760958500	6.91369000259227
C	6.15418704150836	12.48098561809283	8.87955313708270
H	12.60898779217663	12.25973383726754	9.64809928235945
C	12.14258766224123	12.04057884198262	11.72578887785228
C	10.64797560893303	13.06305172556744	9.95979913369472
H	7.95004009139746	12.26020850922246	5.10484963863539
C	8.41597269107451	12.04058065795493	3.02689394727250
C	9.91058167182512	13.06304781233865	4.79289114111009
H	7.67470975812708	9.37006270211867	10.65998728729641
C	8.43769739111561	7.88176174232242	11.99814208006370
C	7.47135829036918	7.47775219297019	9.72166424654065
H	12.88396394723265	9.37033039301151	4.09343293437428
C	12.12082479337245	7.88173778600343	2.75456105966932
C	13.08716117252961	7.47772735486275	5.03103989676680
H	13.57635266444307	7.76042867706833	8.08026972943557
C	13.65671032552259	6.79991156288784	9.99081638915396
C	12.24104310606806	6.06081397886417	8.06815931089098
H	6.98242790143099	7.76008716711942	6.67293167035502
C	6.90181453554063	6.79992795612819	4.76187946891319
C	8.31746676699793	6.06082555067202	6.68453837004763
H	14.74770105451187	9.70276560056663	10.49663258520290
C	15.81806025963945	10.87005307696264	9.04080833115455
H	16.57949722193139	12.13125381328795	7.48138255785985
C	13.86909246001659	13.78334592659691	6.01051591530856
C	14.75819573690969	12.01507115606272	4.58521614474466
H	5.81133532962633	9.70268390813741	4.25606620846059
C	4.73984226560028	10.87008645431155	5.71125732089386

H	3.97918114241743	12.13144170960542	7.27142081804943
C	6.68882978263424	13.78337363632143	8.74154250361839
C	5.79971452670938	12.01510842515238	10.16684666153403
H	12.49709235764575	13.03931514554698	12.00816057777038
H	12.95121822071531	11.32817867544699	11.92473477500420
H	11.29661515437926	11.78751213076164	12.37818612886351
H	11.02562401564565	14.06313229889312	10.19895274038886
H	9.74207519473766	12.91536195919809	10.55884219196167
H	10.36610381899595	13.05854251340929	8.90089559737089
H	8.06179337767289	13.03943024494433	2.74455408992033
H	7.60705887876582	11.32839304648906	2.82843720797034
H	9.26177948994352	11.78705280905609	2.37447924542839
H	9.53250649134990	14.06289185713042	4.55346064695533
H	10.81654198408423	12.91564452563395	4.19384627192364
H	10.19265949505344	13.05912471410142	5.85175564494578
H	7.46574213780024	7.65234885304712	12.45083457114646
H	8.95561239871298	8.59200306833239	12.65348756532958
H	9.02510449579973	6.95452126950722	11.97723924372444
H	6.48644305493787	7.29373360369428	10.16648150442976
H	7.96788878659199	6.50344425618913	9.63503929018756
H	7.30947980133380	7.88243425365386	8.71811444951510
H	13.09257035765122	7.65202281617700	2.30155041950483
H	11.60292557602191	8.59226546930569	2.09954480244056
H	11.53311389119724	6.95471630866657	2.77578280514692
H	14.07206615084918	7.29357463535388	4.58622170893659
H	12.59049655001175	6.50345282261041	5.11739936234986
H	13.24924729945184	7.88188356727881	6.03478577237835
H	14.17793765015174	7.62503681538856	10.48312662330262
H	14.40627314696202	6.05787825994756	9.69104050250003
H	12.98897460864898	6.33020366093667	10.72426848929868
H	13.02796025890826	5.43049436263293	7.63914887812157
H	11.56118698022323	6.34409353399261	7.25785661978175
H	11.68301959444577	5.43477123457678	8.77577661427305
H	6.38034381590876	7.62523106383154	4.27016870117292
H	6.15257779558153	6.05754647647001	5.06157346171106
H	7.56971580983869	6.33080729821266	4.02822052365617
H	7.53028006097747	5.43089793990459	7.11361220435784
H	8.99766938646058	6.34346918694144	7.49480019741577
H	8.87505839448013	5.43447820606999	5.97683807672713
H	16.75254484103238	10.86690243247313	9.59711789186227
C	13.66490508594375	14.56875522079206	4.88100782515265
C	13.56834054997798	14.35526865457816	7.39506528893398
C	14.53050453384819	12.83399056578448	3.48541488841896
C	15.42099565292332	10.64543868551788	4.40039584372907
H	3.80541090103395	10.86709829957492	5.15492859868485
C	6.89229230520389	14.56878435843125	9.87222855061935

C	6.98885541344758	14.35529089280560	7.35817161491460
C	6.02668110869937	12.83402903087617	11.26781578824038
C	5.13617518702154	10.64548090349763	10.35284026631646
H	13.24805342819866	15.56706989280072	4.99751611965402
C	13.97295356637572	14.10288259389836	3.60028929234495
H	13.35054884072067	13.51078272433493	8.06052148608632
C	14.80594032170994	15.07557514636127	7.94238872793432
C	12.36726008518966	15.29864275183145	7.42250464583530
H	14.79517169336019	12.47709771689138	2.49059886463342
H	14.98317792952176	9.96663267617707	5.14716848776423
C	15.22441887076480	10.04552761983681	3.00526047150417
C	16.92980880235105	10.73483393206575	4.68032607214441
H	7.30877817651839	15.56727647832772	9.75557852173862
C	6.58619074773840	14.10291699271036	11.15231849591842
H	7.20696083770350	13.51091570660671	6.69260242485041
C	5.75321059811233	15.07560439164955	6.81021663518597
C	8.19190234422700	15.29865678344221	7.33010018582077
H	5.76152234884727	12.47730891316661	12.26248729390145
H	5.57429353418717	9.96662898988359	9.60614437942797
C	5.33469798484676	10.04557196694568	11.74735738846129
C	3.62931273105375	10.73488565115363	10.07229006270336
C	13.65589081576734	14.93233213250708	2.36180615322922
H	15.07721165301549	15.92042255837393	7.29607394366787
H	15.67142070930635	14.40673527789830	8.00010940089944
H	14.61469988832510	15.46899265540336	8.94953216197654
H	12.56346509141611	16.22641597662434	6.87038564818274
H	12.13928016543493	15.58983488532239	8.45503554222410
H	11.47163004442801	14.83569578576317	6.99364640605639
H	15.83899926378366	10.56652686570417	2.26006943845559
H	14.18553386224782	10.09375102340186	2.65946363520250
H	15.54220631340651	8.99540610403888	2.99735864101741
H	17.40370045669853	11.44321464592426	3.98892205457299
H	17.40637370609472	9.75569103046158	4.54008373083838
H	17.13942614334887	11.06566882274387	5.70052235600345
C	6.90261918659616	14.93236746600233	12.39017949056949
H	5.48170308698273	15.92100397890688	7.45576608317211
H	4.88716919769301	14.40746605466668	6.75207443615667
H	5.94521660823928	15.46852563863642	5.80302061201160
H	7.99631134061474	16.22663601927777	7.88205475285404
H	8.41953513967353	15.58906963807103	6.29731272990908
H	9.08706111902906	14.83487680858649	7.75894743912694
H	4.72052658028569	10.56576527137944	12.49348540636338
H	6.37390997393193	10.09462507358120	12.09195222685772
H	5.01774783849401	8.99518822719490	11.75536628573540
H	3.15453573804014	11.44339665236473	10.76303123639688
H	3.15212222819682	9.75596026863785	10.21226588311600

H	3.42025097904257	11.06551981706904	9.05190309834653
H	14.17097434566971	14.46124631380536	1.51208326945554
C	14.11784185040567	16.37258069821700	2.46600570732979
C	12.15024772484396	14.90589687814112	2.08305626342293
H	6.38729930478887	14.46103513153262	13.23967333190982
C	6.43993774263948	16.37261890541819	12.28714625993902
C	8.40753194061858	14.90592271871302	12.67009935975049
H	13.92998850496124	16.90489858053747	1.52520050452331
H	15.19083013431015	16.44337627799242	2.68279422369680
H	13.58137076907510	16.91376071361401	3.25608386817366
H	11.91327792795720	15.47072134438664	1.17210563118401
H	11.59456115490925	15.36055854788881	2.91337880179727
H	11.78042527512439	13.88172262126597	1.94807867616585
H	6.62731159997561	16.90400647353946	13.22855812192513
H	5.36696808851543	16.44299713207525	12.07015898318503
H	6.97632775227633	16.91488244887699	11.49773759940565
H	8.64383208322526	15.47035304818836	13.58147105625011
H	8.96408637309373	15.36090020145848	11.84049609857080
H	8.77732935447010	13.88171694714439	12.80497706046871
